# Circulating insulin-like growth factors and risks of overall, aggressive and early-onset prostate cancer: a collaborative analysis of 20 prospective studies and Mendelian randomization analysis

**DOI:** 10.1093/ije/dyac124

**Published:** 2022-06-21

**Authors:** Eleanor L Watts, Aurora Perez-Cornago, Georgina K Fensom, Karl Smith-Byrne, Urwah Noor, Colm D Andrews, Marc J Gunter, Michael V Holmes, Richard M Martin, Konstantinos K Tsilidis, Demetrius Albanes, Aurelio Barricarte, H Bas Bueno-de-Mesquita, Barbara A Cohn, Melanie Deschasaux-Tanguy, Niki L Dimou, Luigi Ferrucci, Leon Flicker, Neal D Freedman, Graham G Giles, Edward L Giovannucci, Christopher A Haiman, Graham J Hankey, Jeffrey M P Holly, Jiaqi Huang, Wen-Yi Huang, Lauren M Hurwitz, Rudolf Kaaks, Tatsuhiko Kubo, Loic Le Marchand, Robert J MacInnis, Satu Männistö, E Jeffrey Metter, Kazuya Mikami, Lorelei A Mucci, Anja Olsen, Kotaro Ozasa, Domenico Palli, Kathryn L Penney, Elizabeth A Platz, Michael N Pollak, Monique J Roobol, Catherine A Schaefer, Jeannette M Schenk, Pär Stattin, Akiko Tamakoshi, Elin Thysell, Chiaojung Jillian Tsai, Mathilde Touvier, Stephen K Van Den Eeden, Elisabete Weiderpass, Stephanie J Weinstein, Lynne R Wilkens, Bu B Yeap, The PRACTICAL Consortium, CRUK, BPC3, CAPS, PEGASUS, Rosalind A Eeles, Christopher A Haiman, Zsofia Kote-Jarai, Fredrick R Schumacher, Sara Benlloch, Ali Amin Al Olama, Kenneth R Muir, Sonja I Berndt, David V Conti, Fredrik Wiklund, Stephen Chanock, Ying Wang, Catherine M Tangen, Jyotsna Batra, Judith A Clements, Naomi E Allen, Timothy J Key, Ruth C Travis

**Affiliations:** Cancer Epidemiology Unit, Nuffield Department of Population Health, University of Oxford, Oxford, UK; Cancer Epidemiology Unit, Nuffield Department of Population Health, University of Oxford, Oxford, UK; Cancer Epidemiology Unit, Nuffield Department of Population Health, University of Oxford, Oxford, UK; Genomic Epidemiology Branch, International Agency for Research on Cancer, Lyon, France; Cancer Epidemiology Unit, Nuffield Department of Population Health, University of Oxford, Oxford, UK; Cancer Epidemiology Unit, Nuffield Department of Population Health, University of Oxford, Oxford, UK; Section of Nutrition and Metabolism, International Agency for Research on Cancer, Lyon, France; Clinical Trial Service Unit and Epidemiological Studies Unit (CTSU), Nuffield Department of Population Health, University of Oxford, Oxford, UK; Medical Research Council Population Health Research Unit, University of Oxford, Oxford, UK; Department of Population Health Sciences, Bristol Medical School, University of Bristol, Bristol, UK; MRC Integrative Epidemiology Unit (IEU), Bristol Medical School, University of Bristol, Bristol, UK; National Institute for Health Research (NIHR) Bristol Biomedical Research Centre, University Hospitals Bristol NHS Foundation Trust and Weston NHS Foundation Trust and University of Bristol, Bristol, UK; Department of Epidemiology and Biostatistics, School of Public Health, Imperial College London, London, UK; Department of Hygiene and Epidemiology, University of Ioannina School of Medicine, Ioannina, Greece; Division of Cancer Epidemiology and Genetics, National Cancer Institute, National Institutes of Health, Bethesda, MD, USA; Group of Epidemiology of Cancer and Other Chronic Diseases, Navarra Public Health Institute, Pamplona, Spain; Group of Epidemiology of Cancer and Other Chronic Diseases, Navarra Institute for Health Research (IdiSNA), Pamplona, Spain; CIBER Epidemiology and Public Health CIBERESP, Madrid, Spain; Centre for Nutrition, Prevention and Health Services, National Institute for Public Health and the Environment (RIVM), Utrecht, The Netherlands; Child Health and Development Studies, Public Health Institute, Berkeley, CA, USA; Sorbonne Paris Nord University, Nutritional Epidemiology Research Team, Epidemiology and Statistics Research Center, University of Paris, Bobigny, France; Section of Nutrition and Metabolism, International Agency for Research on Cancer, Lyon, France; National Institute on Aging, Baltimore, MD, USA; WA Centre for Health & Ageing, Medical School, University of Western Australia, Perth, WA, Australia; Western Australian Centre for Health and Ageing, University of Western Australia, Perth, WA, Australia; Division of Cancer Epidemiology and Genetics, National Cancer Institute, National Institutes of Health, Bethesda, MD, USA; Cancer Epidemiology Division, Cancer Council Victoria, Melbourne, VIC, Australia; Centre for Epidemiology and Biostatistics, Melbourne School of Population and Global Health, University of Melbourne, Melbourne, VIC, Australia; Precision Medicine, School of Clinical Sciences at Monash Health, Monash University, Melbourne, VIC, Australia; Department of Epidemiology, Harvard T.H. Chan School of Public Health, Boston, MA, USA; Channing Division of Network Medicine, Brigham and Women's Hospital and Harvard Medical School, Boston, MA, USA; Department of Nutrition, Harvard T.H. Chan School of Public Health, Boston, MA, USA; Department of Preventive Medicine, Center for Genetic Epidemiology, Keck School of Medicine, University of Southern California/Norris Comprehensive Cancer Center, Los Angeles, CA, USA; WA Centre for Health & Ageing, Medical School, University of Western Australia, Perth, WA, Australia; IGFs & Metabolic Endocrinology Group, Translational Health Sciences, Bristol Medical School, University of Bristol, Bristol, UK; Division of Cancer Epidemiology and Genetics, National Cancer Institute, National Institutes of Health, Bethesda, MD, USA; National Clinical Research Center for Metabolic Diseases, Key Laboratory of Diabetes Immunology, Ministry of Education, and Department of Metabolism and Endocrinology, Second Xiangya Hospital of Central South University, Changsha, Hunan, China; Division of Cancer Epidemiology and Genetics, National Cancer Institute, National Institutes of Health, Bethesda, MD, USA; Division of Cancer Epidemiology and Genetics, National Cancer Institute, National Institutes of Health, Bethesda, MD, USA; Division of Cancer Epidemiology, German Cancer Research Center (DKFZ), Heidelberg, Germany; Department of Public Health and Health Policy, Graduate School of Biomedical and Health Sciences, Hiroshima University, Hiroshima, Japan; University of Hawaii, Cancer Center, Honolulu, HI, USA; Centre for Epidemiology and Biostatistics, Melbourne School of Population and Global Health, University of Melbourne, Melbourne, VIC, Australia; Precision Medicine, School of Clinical Sciences at Monash Health, Monash University, Melbourne, VIC, Australia; Department of Public Health and Welfare, Finnish Institute for Health and Welfare, Helsinki, Finland; Department of Neurology, College of Medicine, University of Tennessee Health Science Center, Memphis, TN, USA; Departmemt of Urology, Japanese Red Cross Kyoto Daiichi Hospital, Kyoto, Japan; Department of Epidemiology, Harvard T.H. Chan School of Public Health, Boston, MA, USA; Department of Public Health, Aarhus University, Aarhus, Denmark; Danish Cancer Society, Research Center, Copenhagen, Denmark; Departmemt of Epidemiology, Radiation Effects Research Foundation, Hiroshima, Japan; Cancer Risk Factors and Life-Style Epidemiology Unit, Institute for Cancer Research, Prevention and Clinical Network, Florence, Italy; Department of Epidemiology, Harvard T.H. Chan School of Public Health, Boston, MA, USA; Channing Division of Network Medicine, Brigham and Women's Hospital and Harvard Medical School, Boston, MA, USA; Department of Epidemiology, Johns Hopkins Bloomberg School of Public Health, Baltimore, MD, USA; Departments of Medicine and Oncology, McGill University, Montreal, QC, Canada; Department of Urology, Erasmus University Medical Center, Rotterdam, The Netherlands; Division of Research, Kaiser Permanente Northern California, Oakland, CA, USA; Cancer Prevention Program, Public Health Sciences Division, Fred Hutchinson Cancer Research Center, Seattle, WA, USA; Department of Surgical Sciences, Uppsala University, Uppsala, Sweden; Department of Public Health, Faculty of Medicine, Hokkaido University, Sapporo, Japan; Department of Medical Biosciences, Umeå University, Umeå, Sweden; Department of Radiation Oncology, Memorial Sloan Kettering Cancer Center, New York, NY, USA; Sorbonne Paris Nord University, Nutritional Epidemiology Research Team, Epidemiology and Statistics Research Center, University of Paris, Bobigny, France; Division of Research, Kaiser Permanente Northern California, Oakland, CA, USA; Department of Urology, University of California San Francisco, San Francisco, CA, USA; Director’s Office, International Agency for Research on Cancer, World Health Organization, Lyon, France; Division of Cancer Epidemiology and Genetics, National Cancer Institute, National Institutes of Health, Bethesda, MD, USA; University of Hawaii, Cancer Center, Honolulu, HI, USA; WA Centre for Health & Ageing, Medical School, University of Western Australia, Perth, WA, Australia; Department of Endocrinology and Diabetes, Fiona Stanley Hospital, Perth, WA, Australia; Clinical Trial Service Unit and Epidemiological Studies Unit (CTSU), Nuffield Department of Population Health, University of Oxford, Oxford, UK; UK Biobank Ltd, Stockport, UK; Cancer Epidemiology Unit, Nuffield Department of Population Health, University of Oxford, Oxford, UK; Cancer Epidemiology Unit, Nuffield Department of Population Health, University of Oxford, Oxford, UK

**Keywords:** Insulin-like growth factor-I, prostate cancer, aggressive prostate cancer, prospective analysis, Mendelian randomization, international consortia

## Abstract

**Background:**

Previous studies had limited power to assess the associations of circulating insulin-like growth factors (IGFs) and IGF-binding proteins (IGFBPs) with clinically relevant prostate cancer as a primary endpoint, and the association of genetically predicted IGF-I with aggressive prostate cancer is not known. We aimed to investigate the associations of IGF-I, IGF-II, IGFBP-1, IGFBP-2 and IGFBP-3 concentrations with overall, aggressive and early-onset prostate cancer.

**Methods:**

Prospective analysis of biomarkers using the Endogenous Hormones, Nutritional Biomarkers and Prostate Cancer Collaborative Group dataset (up to 20 studies, 17 009 prostate cancer cases, including 2332 aggressive cases). Odds ratios (OR) and 95% confidence intervals (CI) for prostate cancer were estimated using conditional logistic regression. For IGF-I, two-sample Mendelian randomization (MR) analysis was undertaken using instruments identified using UK Biobank (158 444 men) and outcome data from PRACTICAL (up to 85 554 cases, including 15 167 aggressive cases). Additionally, we used colocalization to rule out confounding by linkage disequilibrium.

**Results:**

In observational analyses, IGF-I was positively associated with risks of overall (OR per 1 SD = 1.09: 95% CI 1.07, 1.11), aggressive (1.09: 1.03, 1.16) and possibly early-onset disease (1.11: 1.00, 1.24); associations were similar in MR analyses (OR per 1 SD = 1.07: 1.00, 1.15; 1.10: 1.01, 1.20; and 1.13; 0.98, 1.30, respectively). Colocalization also indicated a shared signal for IGF-I and prostate cancer (PP4: 99%). Men with higher IGF-II (1.06: 1.02, 1.11) and IGFBP-3 (1.08: 1.04, 1.11) had higher risks of overall prostate cancer, whereas higher IGFBP-1 was associated with a lower risk (0.95: 0.91, 0.99); these associations were attenuated following adjustment for IGF-I.

**Conclusions:**

These findings support the role of IGF-I in the development of prostate cancer, including for aggressive disease.

Key Messages

- We used observational and genetic data from international consortia to investigate the associations of circulating insulin-like growth factors (IGF-I, IGF-II) and their binding proteins (IGFBP-1,-2,-3) with overall, aggressive and early-onset prostate cancer.
- Our findings support the role of IGF-I in the development of prostate cancer, including aggressive disease.
- Our results suggest the need for more research on the modifiable determinants of IGF-I, and whether interventions to lower IGF-I might reduce the risk of prostate cancer.

## Introduction

Prostate cancer is the second most common cancer in men worldwide and a leading cause of cancer death.^1^ Insulin-like growth factors (IGFs) are important growth-promoting peptides that act through the IGF-I receptor.^2^^,^^3^ IGF-I and IGF-II are mainly produced by the liver and circulate in the bloodstream, but they are also produced in local tissues where they function in a paracrine/autocrine manner.^3^ The majority of both of these growth factors circulate bound to IGF proteins (IGFBPs),^2^^,^^4^ which extend the half-life of the IGFs and modulate IGF signalling.^2^^,^^4^ Higher IGF-I signalling increases cell survival and decreases apoptosis, increasing the probability of carcinogenesis.^4^^,^^5^ Circulating IGF-I concentrations are positively associated with risks of several cancers, particularly prostate, breast and colorectal cancer.^6^^,^^7^

The Endogenous Hormones, Nutritional Biomarkers and Prostate Cancer Collaborative Group (EHNBPCCG) is a pooled individual participant nested case-control dataset of prospective studies of hormonal and nutritional factors and prostate cancer risk, which previously reported positive associations of IGF-I, IGF-II, IGFBP-2 and IGFBP-3 with overall prostate cancer risk and an inverse association with IGFBP-1.^8^ However, in this previous study it was unclear whether IGF-II or the IGFBPs are associated with prostate cancer independently of IGF-I, and the analyses of associations with aggressive disease subtypes were underpowered to provide strong evidence of an effect.^8^ The EHNBPCCG dataset has since been expanded to include more than double the number of prostate cancer cases (up to 17 000 prostate cancer cases, including 2300 aggressive cases).

In blood-based observational analyses it is difficult to rule out the possibility of biases including residual confounding or reverse causality. Mendelian randomization (MR) uses germline genetic variants as proxies of putative risk factors and estimates their associations with disease risk. These germline genetic variants are randomly allocated and fixed at conception, and therefore MR is less likely to be affected by these biases and so is potentially a more robust method for causal inference.^9^ In order to appraise causality for IGF-I, we carried out two-sample MR analyses using instruments identified from UK Biobank and genetic data from the PRACTICAL consortium.^10–12^ Using these genetic datasets, we also ran colocalization analyses to investigate whether the *IGF1* gene region and prostate cancer share the same genetic signal to exclude the possibility of confounding by linkage disequilibrium.^13^

Using these two international consortia and UK Biobank, we aimed to assess the associations of circulating IGF-I with overall, aggressive and early-onset prostate cancer risk, using observational and genetic methods. The analysis of very large datasets can provide more robust risk estimates, and the integration of evidence from these different epidemiological approaches can strengthen the basis for causal inference.^14^ We additionally report observational associations of IGF-II and IGFBPs-1,-2,-3 with overall, aggressive and early-onset subtypes.

## Methods

### Endogenous hormones, nutritional biomarkers and Prostate Cancer Collaborative Group

#### Data collection and study designs

Individual participant data were available from up to 20 prospective studies with IGF-I (17 009 cases), IGF-II (4466 cases), IGFBP-1 (4491 cases), IGFBP-2 (3776 cases) and IGFBP-3 (9113 cases) measurements. Participating studies are listed in Supplementary Table S1 and further details of data collection and processing are provided in the Supplementary material. Matching criteria are shown in Supplementary Table S2. Assay details and hormone measurement data are provided in Supplementary Table S3.

#### Data processing and outcomes

Disease definitions were as defined by the PRACTICAL consortium.^10^^,^^11^ Aggressive prostate cancer was categorized as ‘yes’ for any of the following: disease metastases at diagnosis (M1), Gleason score 8+ (or equivalent), prostate cancer death (defined as death from prostate cancer) or prostate-specific antigen (PSA) >100 ng/mL. Early-onset prostate cancer was defined as a diagnosis aged ≤55 years. Further details of the disease characterization can be found in the Supplementary Methods.

#### Statistical analysis

Conditional logistic regression was used to estimate prostate cancer risk by circulating concentrations of IGF-I, IGF-II, IGFBP-1, IGFBP-2 and IGFBP-3. Analyses were conditioned on the study-specific matching variables and adjusted for age at blood collection, body mass index (BMI), height, smoking status, alcohol consumption, racial or ethnic group, education, married/cohabiting and diabetes status. Biomarkers were standardized by study and entered into the model as continuous variables, so each increment represents 1 study-specific SD increase in biomarker concentration. For categorical analyses, biomarkers were categorized into study-specific fifths with cut-points determined in controls.^15^ Further details are available in the Supplementary Methods.

#### Further analyses

We examined heterogeneity in the associations of each biomarker with prostate cancer by participant characteristics, with subgroups defined a priori based on the availability of data and previous analyses using this dataset^8^^,^^16^; heterogeneity in the associations by study was also examined (Supplementary Methods). We additionally investigated unadjusted matched associations, associations in tenths, and estimates per 80th percentile increase. Associations were also examined following mutual adjustment for other biomarkers (IGF-I, IGF-II, IGFBP-1,-2,-3, free and total testosterone and sex hormone-binding globulin [SHBG]), and we tested for interactions between these biomarkers; further details are available in the Supplementary Methods. Stratified analyses and associations in tenths were not investigated for early-onset disease due to the limited number of cases.

### Mendelian randomization analysis

#### Genetic instruments for hormone concentrations

Single nucleotide polymorphisms (SNPs) associated with circulating IGF-I concentrations were identified from a publicly available genome-wide association study (GWAS) based on 158 444 male UK Biobank participants of White British ancestry (*P *<5 x 10^–8^ significance threshold).^17^ We pruned SNPs by a linkage disequilibrium threshold of r^2^<0.001, based on the lowest *P*-value.

#### Genetic associations with prostate cancer

SNP associations for prostate cancer were obtained from the PRACTICAL and GAME-ON/ELLIPSE consortia,^10^^,^^11^ which currently do not include UK Biobank data. Individual studies included in these consortia are detailed in Conti *et al.*^12^ and Schumacher *et al.*^10^ Associations with overall prostate cancer risk were generated from 85 554 prostate cancer cases and 91 972 controls,^12^ with aggressive disease from 15 167 cases and 58 308 controls and with early-onset disease from 6988 cases and 44 256 controls,^10^ all of White European ancestry.

#### Statistical analysis

The MR estimation for hormones was conducted using the inverse-variance weighted (IVW) method.^18^ We additionally calculated the I^2^ statistic to assess measurement error in SNP-exposure associations,^19^ the F statistic to assess instrument strength,^20^^,^^21^ Cochran’s Q statistic to test for heterogeneity between the MR estimates for each SNP^22^ and PhenoScanner was used to assess pleiotropy of the genetic instruments.^23^ As sensitivity analyses, we used the MR residual sum and outlier (MR-PRESSO), MR robust adjusted profile score (MR-RAPS) and leave-one-out analyses to investigate the role of SNP outliers.^24^ To assess pleiotropy, we used the weighted median, MR-Egger and the MR-Egger intercept.^25^ We also used the contamination mixture method, which assumes a normal distribution of valid instruments around the true causal value, and invalid instruments are normally distributed around zero in order to account for potentially pleiotropic variants.^26^ To rule out reverse causality, analyses were repeated after applying Steiger filtering which excludes variants with larger effects on prostate cancer risk than on IGF-I.^27^

The associations of the IGF-I *cis*-SNP, defined as the lead SNP on the biomarker gene coding region identified from the exposure datasets, with prostate cancer were investigated using the Wald ratio. This *cis*-SNP is less likely than *trans*-SNPs to be affected by horizontal pleiotropy.^28^

### Colocalization analysis

Colocalization was used to investigate whether the associations of variation in the *IGF1* gene region with both circulating IGF-I concentration and prostate cancer risk, share the same genetic signal or whether the associations identified by our MR analysis may be confounded by linkage disequilibrium.^13^ Analyses were conducted for a 75-kb region surrounding the lead IGF-I *cis-*SNP (rs5742653) using the UK Biobank and PRACTICAL datasets.^12^^,^^17^ Colocalization was assessed using three approaches: conventional colocalization,^13^ which tests for the presence of a single shared genetic signal; as well as the sum of single effects (SuSiE) regression framework^29^; and conditional iterative colocalization.^30^ The latter two methods allow for the possibility of multiple independent (but partially correlated) causal variants in proximity.^31^ We created colocalization plots using LocusCompareR^32^ and a z-z locus plot.^33^ We considered a posterior probability of a shared causal variant (PP4) of >0.7 as being consistent with evidence of colocalization between IGF-I and prostate cancer.^13^ Further details of the colocalization analysis are available in the Supplementary Methods.

Details of statistical software and packages used are available in the Supplementary Methods. All tests of significance were two-sided, and *P*-values <0.05 were considered statistically significant.

## Results

### Study and participant characteristics in the observational analyses

A total of 20 studies, contributing up to 17 009 cases and 37 243 controls, were included in this analysis. Prostate cancer was classified as aggressive in 2332 cases and early-onset disease in 607 cases. Study participants were 91.3% of White ethnicity (Table 1). Men who were diagnosed with overall prostate cancer were taller and had a lower BMI than their matched controls (Table 1).

**Table 1:** Characteristics of prostate cancer cases and controls in the EHNBPCCG participants

	Controls	Cases		
		Overall	Aggressive^a^	Early-onset^b^
*N*	37 243	17 009	2332	607
Age (years), mean (SD)	61.4 (7.7)	60.7 (8.0)	61.2 (7.9)	47.1 (5.3)
Height (cm), mean (SD)	174.9 (7.0)	175.3 (7.1)	175.2 (7.3)	177.3 (6.9)
BMI (kg/m^2^), mean (SD)	27.4 (4.1)	26.8 (3.6)	26.9 (3.9)	26.3 (3.6)
PSA at blood collection (ng/mL), mean (IQR)	0.9 (1.2)	2.4 (3.3)	2.9 (5.7)	1.9 (2.8)
Time from blood collection to diagnosis, mean (SD)	–	6.7 (5.4)	8.0 (6.3)	5.6 (5.0)
Age at diagnosis, mean (SD)	–	67.5 (6.5)	67.3 (6.2)	52.7 (2.4)
Racial/ethnic group, *N* (%)				
White	33 988 (91.3)	15 617 (91.8)	2217 (95.1)	532 (87.6)
Black	1145 (3.1)	505 (3.0)	53 (2.3)	30 (4.9)
East Asian	336 (0.9)	146 (0.9)	8 (0.3)	3 (0.5)
Other	707 (1.9)	266 (1.6)	22 (0.9)	11 (1.8)
Not known	1067 (2.9)	475 (2.8)	32 (1.4)	31 (5.1)
Smoking status, *N* (%)				
Never	14 985 (40.2)	6791 (39.9)	804 (34.5)	305 (50.2)
Ex	16 511 (44.3)	7300 (42.9)	1000 (42.9)	170 (28.0)
Current	5203 (14.0)	2533 (14.9)	491 (21.1)	127 (20.9)
Not known	544 (1.5)	385 (2.3)	37 (1.6)	5 (0.8)
Alcohol consumption (g ethanol/day), *N* (%)				
Non-drinker	2851 (7.7)	1806 (10.6)	264 (11.3)	52 (8.6)
<10	9073 (24.4)	4535 (26.7)	649 (27.8)	162 (26.7)
10 +	21 385 (57.4)	9171 (53.9)	1284 (55.1)	346 (57.0)
Not known	3934 (10.6)	1497 (8.8)	135 (5.8)	47 (7.7)
Diabetes status, *N* (%)				
Yes	2921 (7.8)	864 (5.1)	127 (5.4)	12 (2.0)
No	31 707 (85.1)	14 847 (87.3)	2052 (88.0)	533 (87.8)
Not known	2615 (7.0)	1298 (7.6)	153 (6.6)	62 (10.2)
Married/cohabiting, *N* (%)				
Yes	9478 (25.4)	6810 (40.0)	1157 (49.6)	235 (38.7)
No	1407 (3.8)	922 (5.4)	149 (6.4)	40 (6.6)
Not known	26 358 (70.8)	9277 (54.5)	1026 (44.0)	332 (54.7)

BMI, body mass index; EHNBPCCG, Endogenous Hormones, Nutritional Biomarkers and Prostate Cancer Collaborative Group; IQR, interquartile range; PSA, prostate-specific antigen; SD, standard deviation.

a Aggressive disease was defined as Gleason Score 8+, death from prostate cancer, metastatic disease or PSA >100 ng/mL.

b Onset defined as diagnosed aged ≤55 years.

Prostate cancer characteristics by study are displayed in Supplementary Table S4. Mean age at blood collection for each study ranged from 33.8 to 76.8 years (overall mean = 61.2 years, SD = 7.8 years). Cases were diagnosed on average 6.7 years (SD = 5.4) after blood collection, and the average age at diagnosis was 67.5 years (SD = 6.5) (Table 1). Aggressive disease was diagnosed on average 8.0 years after blood collection (SD = 6.3) (Table 1). Partial correlations between biomarkers ranged from r = −0.004 (PSA and IGF-II) to r = 0.54 (IGF-II and IGFBP-2) (Supplementary Table S5).

### IGF-I

In observational analyses, higher IGF-I was related to dose-dependent elevated risks of overall (OR per 1 SD increment = 1.09: 95% CI 1.07, 1.11; *P *<0.0001) and aggressive prostate cancer (1.09: 1.03, 1.16; *P *=* *0.01), and there was a suggestive association with early-onset disease (1.11: 1.00, 1.24; *P *=* *0.05).

**Figure 1:**
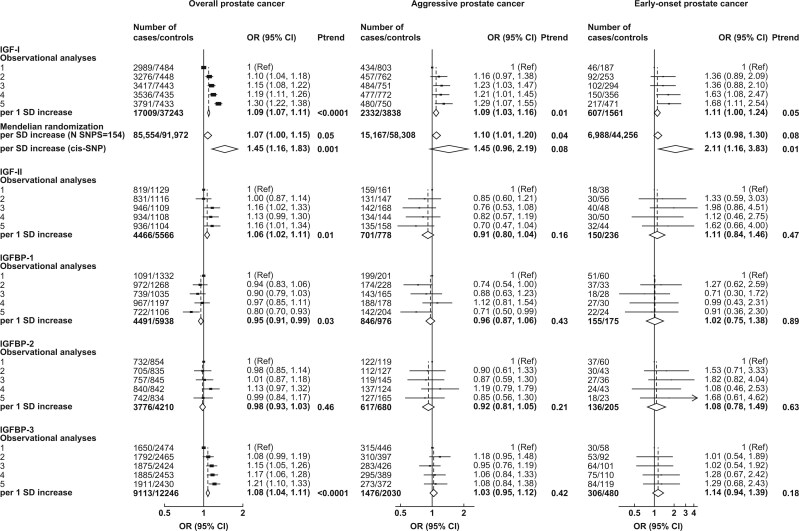
Risks of overall, aggressive* and early-onset† prostate cancer by study-specific fifths of biomarker concentrations (observational only) and 1 SD increment (observational and Mendelian randomization). Estimates are from logistic regression conditioned on the matching variables and adjusted for age, BMI, height, alcohol intake, smoking status, marital status, education status, racial/ethnic group and diabetes status. The position of each square indicates the magnitude of the odds ratio, and the area of the square is proportional to the inverse of the variance of the log odds ratio. The length of the horizontal line through the square indicates the 95% confidence interval. MR risk estimates are estimated using the inverse-variance weighted method for the full instrument methods and the Wald ratio in the *cis*-SNP analyses. *P*_trend_ represents 1-SD increase in biomarker concentration. *Aggressive cancer defined as Gleason grade 8+, or prostate cancer death, or metastases or PSA >100 ng/mL. †Early-onset defined as diagnosed ≤55 years. BMI, body mass index; CI, confidence interval; IGF, insulin-like growth factor; IGFBP, insulin-like growth factor-binding protein; OR, odds ratio; PSA, prostate-specific antigen; SD, standard deviation; MR, Mendelian randomization; SNP, single nucleotide polymorphism

In MR analyses, higher IGF-I was associated with increased risks of overall and aggressive disease (OR per genetically predicted 1-SD increment = 1.07: 1.00, 1.15; *P *=* *0.05; and 1.10: 1.01, 1.20; *P *=* *0.04, respectively) and was positively related to risk of early-onset disease (1.13: 0.98, 1.30; *P *=* *0.08) (Figure 1). The MR sensitivity analyses were generally directionally consistent with IGF-I, although the confidence intervals were wider (Table 2).

**Table 2:** Mendelian randomization estimates between genetically predicted circulating IGF-I concentrations and overall, aggressive and early-onset prostate cancer

			Overall prostate cancer		Aggressive prostate cancer^a^		Early-onset prostate cancer^b^	
			(85 554 cases, 91 972 controls)		(15 167 cases, 58 308 controls)		(6988 cases, 44 256 controls)	
	Variance explained	*N* SNPs	OR per 1-SD increment (95% CI)	*P*-value	OR per 1-SD increment (95% CI)	*P*-value	OR per 1-SD increment (95% CI)	*P*-value
IGF-I (SD = 5.4 nmol/L)								
Inverse-variance weighted	8.7%	154	1.07 (1.00, 1.15)	0.05	1.10 (1.01, 1.20)	0.04	1.13 (0.98, 1.30)	0.08
Weighted median			1.01 (0.95, 1.08)	0.71	1.03 (0.91, 1.16)	0.63	1.07 (0.90, 1.29)	0.44
MR-Egger			1.00 (0.85, 1.17)	0.99	1.01 (0.83, 1.24)	0.90	0.98 (0.71, 1.35)	0.89
MR-Egger intercept				0.73		0.38		0.31
MR-RAPS			1.04 (0.98, 1.12)	0.22	1.11 (1.00, 1.22)	0.04	1.11 (0.96, 1.28)	0.16
MR-PRESSO			1.06 (1.00, 1.12)	0.05	1.08 (0.99, 1.18)	0.08	1.10 (0.97, 1.25)	0.13
Contamination mixture			1.01 (0.90, 1.06)	0.73	1.32 (1.17, 1.45)	0.0005	1.13 (0.96, 1.42)	0.16
*cis*-SNP (rs5742653)	0.2%	1	1.45 (1.16, 1.83)	0.001	1.45 (0.96, 2.19)	0.08	2.11 (1.16, 3.83)	0.01

SD estimates based on UK Biobank males.

CI, confidence interval; IGF-I, insulin-like growth factor-I; MR, Mendelian randomization; OR, odds ratio; PRESSO, pleiotropy residual sum and outlier; PSA, prostate-specific antigen; RAPS, robust adjusted profile score; SD, standard deviation; SNP, single nucleotide polymorphism.

a Aggressive disease was defined as Gleason Score 8+, death from prostate cancer, metastatic disease or PSA >100 ng/mL.

b Early-onset defined as diagnosed aged ≤55 years.

The associations with prostate cancer risk were also directionally consistent when IGF-I was proxied by the *cis-*SNP (rs5742653) (1.45: 1.16, 1.83; P = 0.001; 1.45: 0.96–2.19; *P *=* *0.08; and 2.11: 1.16, 3.83; *P *=* *0.01, for overall, aggressive and early-onset disease, respectively) (Figure 1). Both SuSiE and conditional iterative analyses indicated multiple independent shared causal variants for IGF-I and overall prostate cancer (maximum PP4 >0.99 using SuSiE and PP4 = 0.72 using conditional iterative regression) (Supplementary Table S6 and Supplementary Figures S1 and S2).

### IGF-II and IGFBPs-1–3

In observational analyses, men with higher circulating IGF-II and IGFBP-3 had an elevated risk of overall prostate cancer (OR per 1-SD increment = 1.06: 95% CI 1.02, 1.11; *P *=* *0.01; and 1.08: 1.04, 1.11; *P *<0.0001, respectively). IGFBP-1 was inversely associated with overall prostate cancer (0.95: 0.91, 0.99; *P *=* *0.03), and IGFBP-2 was not associated with prostate cancer risk (0.98: 0.93, 1.03; *P *=* *0.46) (Figure 1). These biomarkers were not associated with aggressive or early-onset disease (Figure 1).

#### Further analyses—observational analysis

**Figure 2:**
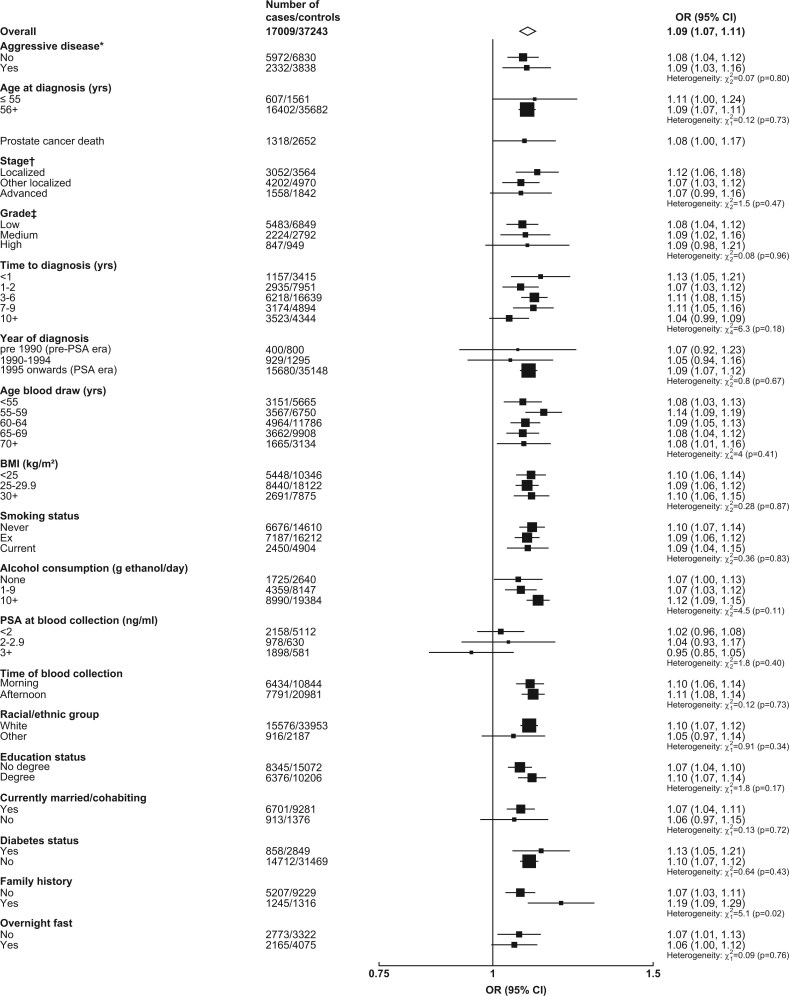
Odds ratio (95% CIs) for overall prostate cancer per study-specific 1-SD increment of IGF-I concentration by subgroup in the EHNBPCCG. Estimates are from logistic regression conditioned on the matching variables and adjusted for age, BMI, height, alcohol intake, smoking status, marital status, education status, racial/ethnic group and diabetes status. The position of each square indicates the magnitude of the odds ratio, and the area of the square is proportional to the inverse of the variance of the log odds ratio. The length of the horizontal line through the square indicates the 95% confidence interval. Tests for heterogeneity for case-defined factors were obtained by fitting separate models for each subgroup and assuming independence of the ORs using a method analogous to a meta-analysis. Tests for heterogeneity for non-case-defined factors were assessed with a χ^2^ test of interaction between subgroup and the binary variable. *Aggressive cancer defined as Gleason grade 8+, or prostate cancer death, or metastases or PSA >100 ng/mL. †Localized defined as TNM stage <T2 with no reported lymph node involvement or metastases or stage I; other localized stage if TNM stage T2 with no reported lymph node involvement or metastases, stage II, or equivalent; advanced stage if they were TNM stage T3 or T4 and/or N1+ and/or M1, stage III–IV or equivalent. ‡ Low grade defined as Gleason score was <7 or equivalent (i.e. extent of differentiation good, moderate); medium grade if Gleason score was 7 (i.e. poorly differentiated); high grade if the Gleason score was ≥8 or equivalent (i.e. undifferentiated). BMI, body mass index; CI, confidence interval; EHNBPCCG, Endogenous Hormones, Nutritional Biomarkers and Prostate Cancer Collaborative Group; IGF-I, insulin-like growth factor-I; OR, odds ratio; PSA, prostate-specific antigen; SD, standard deviation; TNM, tumour, node, metastases

**Figure 3:**
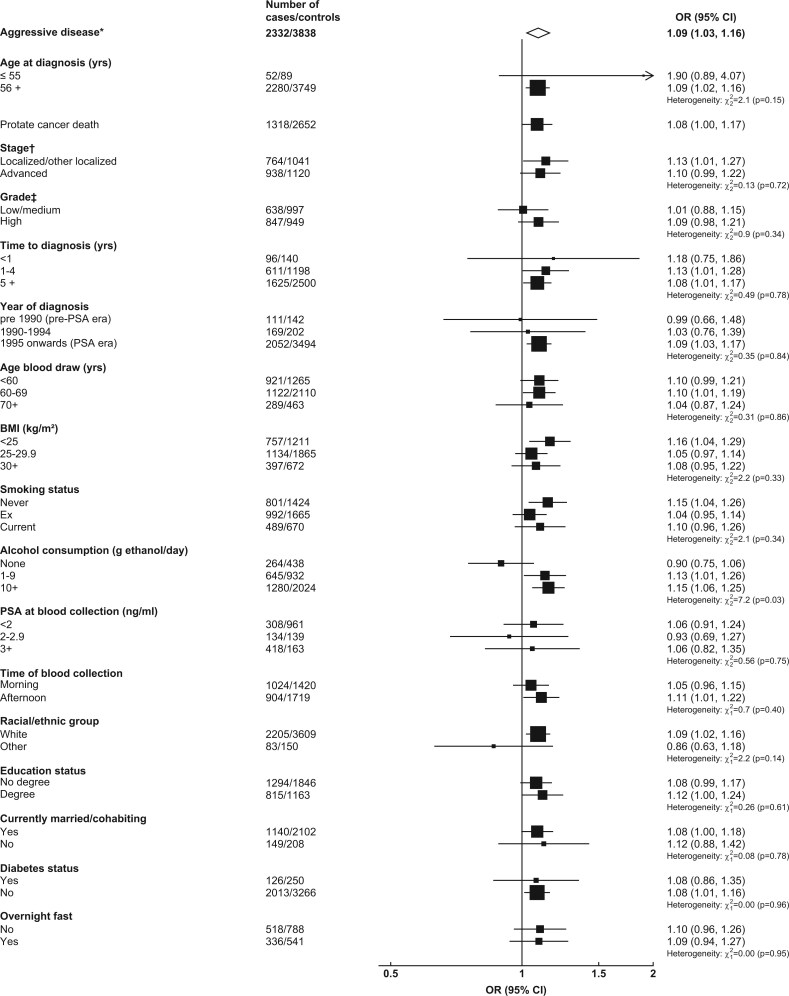
Odds ratio (95% CIs) for aggressive* prostate cancer per study-specific 1-SD increment of IGF-I concentration by subgroup in the EHNBPCCG. Estimates are from logistic regression conditioned on the matching variables and adjusted for age, BMI, height, alcohol intake, smoking status, marital status, education status, racial/ethnic group and diabetes status. The position of each square indicates the magnitude of the odds ratio, and the area of the square is proportional to the inverse of the variance of the log odds ratio. The length of the horizontal line through the square indicates the 95% confidence interval. Tests for heterogeneity for case-defined factors were obtained by fitting separate models for each subgroup and assuming independence of the ORs using a method analogous to a meta-analysis. Tests for heterogeneity for non-case-defined factors were assessed with a χ^2^ test of interaction between subgroup and the binary variable. *Aggressive cancer defined as Gleason grade 8+, or prostate cancer death, or metastases or PSA >100 ng/mL. †Localized/other localized defined as TNM stage <T2 with no reported lymph node involvement or metastases or stage I, or TNM stage T2 with no reported lymph node involvement or metastases, stage II, or equivalent; advanced stage if they were TNM stage T3 or T4 and/or N1+ and/or M1, stage III–IV or equivalent. ‡Low/medium grade defined as Gleason score was <8 or equivalent (i.e. extent of differentiation good, moderate, poor); high grade if the Gleason score was ≥8 or equivalent (i.e. undifferentiated). BMI, body mass index; CI, confidence interval; EHNBPCCG, Endogenous Hormones, Nutritional Biomarkers and Prostate Cancer Collaborative Group; IGF, insulin-like growth factor-I; OR, odds ratio; PSA, prostate-specific antigen; SD, standard deviation; TNM, tumour, node, metastases

Associations of IGF-I with overall and aggressive prostate cancer were generally consistent by subgroups and secondary outcomes (Figures 2 and 3). The OR for prostate cancer death was 1.08 for IGF-I (1.00, 1.17) (Figure 2). There was some evidence of larger magnitudes of associations with overall prostate cancer for men with a family history of prostate cancer (1.19: 1.09, 1.29) than for men without (1.07: 1.03, 1.11; *P*_het_ = 0.02) (Figure 2).

The associations of IGF-II and IGFBPs with prostate cancer risk were broadly similar by subgroups (Supplementary Figures S3–S10). There was evidence of heterogeneity in the association of IGFBP-2 with overall prostate cancer by BMI (*P*_het_ = 0.0007); for men whose BMI was <25 kg/m^2^ at baseline, IGFBP-2 was inversely associated with prostate cancer (0.89: 0.83, 0.96), and the OR for men with BMI 30+ was 1.19 (0.99, 1.42) (Supplementary Figure S7). IGFBP-2 was also inversely associated with aggressive disease risk for men whose BMI was <25 kg/m^2^ (0.78: 0.66, 0.94), but not for men who had a higher BMI (*P*_het_ = 0.01) (Supplementary Figure S8).

Associations with overall and aggressive prostate cancer by study are available in Supplementary Figures S11–S20. There was some evidence of heterogeneity by study in the associations of IGF-I with aggressive disease (*P*_het_ = 0.02) (Supplementary Figure S12), and IGF-II and IGFBP-2 with overall prostate cancer risk (*P*_het_ = 0.0001 and 0.02, respectively) (Supplementary Figures S13 and S17). Associations were broadly similar to the primary analyses in unadjusted matched analyses (Supplementary Figure S21), using study-specific tenths (Supplementary Figure S22) and per 80 percentile increase (Supplementary Table S7). Following mutual adjustment for IGF-I, the associations of IGF-II and IGFBP-1 with risk were attenuated to the null (Supplementary Table S8). For IGF-I and IGFBP-3, mutual adjustment slightly attenuated the associations with overall prostate cancer risk, but both these associations remained (Supplementary Table S8).

There was some evidence of interactions in the associations of IGF-II, IGFBP-1 and IGFBP-2 concentrations with prostate cancer risk by total testosterone concentrations; men with total testosterone concentrations above the study-specific median showed evidence of a positive relationship for IGF-II and an inverse association for IGFBP-1, whereas these associations were null for men with lower total testosterone concentrations (*P*_het_ = 0.03 and 0.02, respectively) (Supplementary Table S9). Only men with lower total testosterone concentrations had a positive association between IGFBP-2 and overall prostate cancer (*P*_het_ = 0.01). For aggressive disease, the OR for IGFBP-2 was 1.27 for men with lower total testosterone concentrations (1.00, 1.62), and in men with higher total testosterone there was an inverse relationship of IGFBP-2 with aggressive disease (0.75: 0.60, 0.93; *P*_het_ <0.01), although the number of aggressive cases was limited (*N *=* *443) (Supplementary Table S10).

#### Further analyses—mendelian randomization

There was no strong evidence of measurement error in the genetic instruments for IGF-I (I^2^ = 0.99) and all SNPs had an F statistic >10.^20^ There was significant heterogeneity in the MR estimates for the SNPs with overall prostate cancer, and for aggressive and early-onset disease (Cochran’s Q *P *<0.001). Full MR results are found in Supplementary Table S11. Forest plots of single-SNP results are available in Supplementary Figures S23–25, leave-one-out plots are available in Supplementary Figures S26–28 and MR scatterplots are available in Supplementary Figure S29. Outliers identified by MR-PRESSO are available in Supplementary Table S12. Following Steiger filtering, the results were slightly attenuated (Supplementary Table S13). Using PhenoScanner, 430 traits were identified as being linked to genetically predicted IGF-I, including height and measures of adiposity (Supplementary Figure S30). Higher concentrations of IGF-I instrumented by the *cis*-SNP (rs5742653) were associated with increased peak expiratory flow (*P *<5 x 10^–8^).

## Discussion

This is the first study that has applied both observational and genetic approaches using data from large international consortia to investigate the associations of IGF-I with prostate cancer risk. Our results support a role of circulating IGF-I in the development of prostate cancer, including aggressive disease. In observational analyses, IGF-II and IGFBPs-1 and -3 were also associated with overall prostate cancer risk, but these associations were attenuated following adjustment for IGF-I.

Genetic analyses may be more informative than observational analyses about the direct role of the exposure on the outcome. The weaker findings from genetic analyses from the multi-SNP (*cis* and *trans*) instrument, compared with the *cis*-SNP may be related to associations of some of the *trans-*SNPs with other components of the IGF signalling pathway such as the IGFBPs.^34^ For the lead *cis*-SNP MR we observed larger magnitude effects, which likely indicates stronger biological plausibility of a direct role for IGF-I and a reduced role of horizontal pleiotropy,^35^ and may also be due to the possible role of local *IGF1* expression in the prostate tissue. Moreover, colocalization analyses showed strong evidence of a shared genetic cause at the *IGF1* gene for IGF-I concentrations and risk for prostate cancer, indicating that our findings are unlikely to be due to confounding by linkage disequilibrium.

In our observational analyses, IGF-II, IGFBP-1 and IGFBP-3 were positively associated with overall prostate cancer, but we were underpowered to detect associations with aggressive or early-onset disease. Following further adjustment for IGF-I, the associations with overall disease were attenuated although IGFBP-3 remained significantly associated with overall prostate cancer. These results suggest that the observed associations may be at least partially due to the correlations of these biomarkers with IGF-I. Analogous genetic approaches such as multivariable MR may be useful in exploring the direct and indirect effects of these biomarkers on prostate cancer risk.^36^

These analyses have several strengths. This is the largest collection of observational and genetic data on hormones and prostate cancer risk available, representing almost all the available data worldwide. This large sample size maximizes power to assess associations robustly and enabled us to investigate associations across subgroups. Further, by incorporating observational and genetic methods, we were able to use different lines of evidence for a more robust investigation towards causal inference.^14^

This study had a number of limitations. IGFs and IGFBPs are also produced locally as well as by the liver, which may affect prostate cancer risk independently of circulating concentrations.^2^^,^^4^ Consequently, the predictive value of circulating IGF-I as an indicator of intra-prostatic IGF signalling remains incompletely understood,^4^ and future research including measured intra-prostatic IGF-I and IGF-I receptor expression may help to clarify this. Our analyses relied on single biomarker measurements, and although these biomarkers have good reproducibility over a 1 to 5 year period (intraclass correlation coefficients 0.60–0.90 for IGF-I and IGFBP-1,-2,-3),^37–39^ this would be expected to lead to underestimates of risk in the observational analyses.^40^ Although associations were generally consistent by subgroup, the number of statistical tests in these analyses increased the possibility of false-positives. Assay methods used to measure the biomarkers varied by study, and some IGF biomarkers are more difficult to measure than others (for example, IGF-II); measurement error would be expected to be non-differential and therefore tend to bias associations towards the null. As in the standard approach for MR, effect estimates were calculated on the same scale as for the observational analyses, and this scaling‐up results in some imprecision with wide confidence intervals in the associations; the concordance of the directions of the associations is therefore particularly important. Wider confidence intervals in MR sensitivity analyses may relate to lower power for some of these methods.^41^

## Conclusion

In conclusion, the findings from these analyses using observational and genetic data from large-scale international consortia are supportive of a role of IGF-I in the aetiology of prostate cancer. For the first time we show evidence that IGF-I is important for aggressive, clinically relevant disease. These findings support the need for more research on the modifiable determinants of IGF-I, and on whether interventions to lower IGF-I might reduce the risk of prostate cancer.

## PRACTICAL, CRUK, BPC3, CAPS and PEGASUS consortia investigators

Principal Investigators from the PRACTICAL [http://practical.icr.ac.uk/], CRUK, BPC3, CAPS, PEGASUS consortia: Rosalind A Eeles,^1,2^ Christopher A Haiman,^3^ Zsofia Kote-Jarai,^1^ Fredrick R Schumacher,^4,5^ Sara Benlloch,^1,6^ Ali Amin Al Olama,^6,7^ Kenneth R Muir,^8^ Sonja I Berndt,^9^ David V Conti,^3^ Fredrik Wiklund,^10^ Stephen Chanock,^9^ Ying Wang,^11^ Catherine M Tangen,^12^ Jyotsna Batra,^13,14^ Judith A Clements,^13,14^ APCB BioResource (Australian Prostate Cancer BioResource),^15,14^ Henrik Grönberg,^10^ Nora Pashayan,^16,17^ Johanna Schleutker,^18,19^ Demetrius Albanes,^9^ Stephanie Weinstein,^9^ Alicja Wolk,^20^ Catharine M L West,^21^ Lorelei A Mucci,^22^ Géraldine Cancel-Tassin,^23,24^ Stella Koutros,^9^ Karina Dalsgaard Sørensen,^25,26^ Eli Marie Grindedal,^27^ David E Neal,^28,29,30^ Freddie C Hamdy,^31,32^ Jenny L Donovan,^33^ Ruth C Travis,^34^ Robert J Hamilton,^35,36^ Sue Ann Ingles,^37^ Barry S Rosenstein,^38^ Yong-Jie Lu,^39^ Graham G Giles,^40,41,42^ Robert J MacInnis,^40,41^ Adam S Kibel,^43^ Ana Vega,^44,45,46^ Manolis Kogevinas,^47,48,49,50^ Kathryn L Penney,^51^ Jong Y Park,^52^ Janet L Stanford,^53,54^ Cezary Cybulski,^55^ Børge G Nordestgaard,^56,57^ Sune F Nielsen,^56,57^ Hermann Brenner,^58,59,60^ Christiane Maier,^61^ Jeri Kim,^62^ Esther M John,^63^ Manuel R Teixeira,^64,65,66^ Susan L Neuhausen,^67^ Kim De Ruyck,^68^ Azad Razack,^69^ Lisa F Newcomb,^53,70^ Davor Lessel,^71^ Radka Kaneva,^72^ Nawaid Usmani,^73,74^ Frank Claessens,^75^ Paul A Townsend,^76,77^ Jose Esteban Castelao,^78^ Monique J Roobol,^79^ Florence Menegaux,^80^ Kay-Tee Khaw,^81^ Lisa Cannon-Albright,^82,83^ Hardev Pandha,^77^ Stephen N Thibodeau,^84^ David J Hunter,^85^ Peter Kraft,^86^ William J Blot^87,88^ and Elio Riboli^89^

^1^Institute of Cancer Research, London, UK

^2^Royal Marsden NHS Foundation Trust, London, UK

^3^Center for Genetic Epidemiology, Department of Preventive Medicine, Keck School of Medicine, University of Southern California/Norris Comprehensive Cancer Center, Los Angeles, CA, USA

^4^Department of Population and Quantitative Health Sciences, Case Western Reserve University, Cleveland, OH, USA

^5^Seidman Cancer Center, University Hospitals, Cleveland, OH, USA

^6^Centre for Cancer Genetic Epidemiology, Department of Public Health and Primary Care, University of Cambridge, Cambridge, UK

^7^University of Cambridge, Department of Clinical Neurosciences, Stroke Research Group, Cambridge, UK

^8^Division of Population Health, Health Services Research and Primary Care, University of Manchester, Manchester, UK

^9^Division of Cancer Epidemiology and Genetics, National Cancer Institute, Bethesda, MD, USA

^10^Department of Medical Epidemiology and Biostatistics, Karolinska Institute, Stockholm, Sweden

^11^Department of Population Science, American Cancer Society, Atlanta, GA, USA

^12^SWOG Statistical Center, Fred Hutchinson Cancer Research Center, Seattle, WA, USA

^13^Australian Prostate Cancer Research Centre-QLD, Institute of Health and Biomedical Innovation and School of Biomedical Sciences, Queensland University of Technology, Brisbane, QLD, Australia

^14^Translational Research Institute, Brisbane, QLD, Australia

^15^Australian Prostate Cancer Research Centre-QLD, Queensland University of Technology, Brisbane; Prostate Cancer Research Program, Monash University, Melbourne; Dame Roma Mitchell Cancer Centre, University of Adelaide, Adelaide; Chris O'Brien Lifehouse, Royal Prince Alfred Hospital, Camperdown: Australia

^16^Department of Applied Health Research, University College London, London, UK

^17^Centre for Cancer Genetic Epidemiology, Department of Oncology, University of Cambridge, Cambridge, UK

^18^Institute of Biomedicine, University of Turku, Finland

^19^Department of Medical Genetics, Turku University Hospital, Turku, Finland

^20^Department of Surgical Sciences, Uppsala University, Uppsala, Sweden

^21^Division of Cancer Sciences, University of Manchester, Manchester Academic Health Science Centre, Radiotherapy Related Research, Christie Hospital NHS Foundation Trust, Manchester, UK

^22^Department of Epidemiology, Harvard T.H. Chan School of Public Health, Boston, MA, USA

^23^CeRePP, Tenon Hospital, Paris, France

^24^Sorbonne Universite, GRC n°5, AP-HP, Tenon Hospital,Paris, France

^25^Department of Molecular Medicine, Aarhus University Hospital, Aarhus N, Denmark

^26^Department of Clinical Medicine, Aarhus University, Aarhus N, Denmark

^27^Department of Medical Genetics, Oslo University Hospital, Oslo, Norway

^28^Nuffield Department of Surgical Sciences, University of Oxford, John Radcliffe Hospital, Oxford, UK

^29^University of Cambridge, Department of Oncology, Addenbrooke's Hospital, Cambridge, UK

^30^Cancer Research UK, Cambridge Research Institute, Li Ka Shing Centre, Cambridge, UK

^31^Nuffield Department of Surgical Sciences, University of Oxford, Oxford, UK

^32^Faculty of Medical Science, University of Oxford, John Radcliffe Hospital, Oxford, UK

^33^Population Health Sciences, Bristol Medical School, University of Bristol, UK

^34^Cancer Epidemiology Unit, Nuffield Department of Population Health, University of Oxford, Oxford, UK

^35^Dept. of Surgical Oncology, Princess Margaret Cancer Centre, Toronto, ON, Canada

^36^Dept. of Surgery (Urology), University of Toronto, Toronto, ON, Canada

^37^Department of Preventive Medicine, Keck School of Medicine, University of Southern California/Norris Comprehensive Cancer Center, Los Angeles, CA, USA

^38^Department of Radiation Oncology and Department of Genetics and Genomic Sciences, Icahn School of Medicine at Mount Sinai, New York, NY, USA

^39^Centre for Cancer Biomarker and Biotherapeutics, Barts Cancer Institute, Queen Mary University of London, John Vane Science Centre, London, UK

^40^Cancer Epidemiology Division, Cancer Council Victoria, Melbourne, VIC, Australia

^41^Centre for Epidemiology and Biostatistics, Melbourne School of Population and Global Health, University of Melbourne, Parkville, VIC, Australia

^42^Precision Medicine, School of Clinical Sciences at Monash Health, Monash University, Clayton, VIC, Australia

^43^Division of Urologic Surgery, Brigham and Womens Hospital, Boston, MA, USA

^44^Fundación Pública Galega Medicina Xenómica, Santiago de Compostela, Spain

^45^Instituto de Investigación Sanitaria de Santiago de Compostela, Santiago De Compostela, Spain

^46^Centro de Investigación en Red de Enfermedades Raras, Madrid, Spain

^47^ISGlobal, Barcelona, Spain

^48^IMIM (Hospital del Mar Medical Research Institute), Barcelona, Spain

^49^Universitat Pompeu Fabra , Barcelona, Spain

^50^CIBER Epidemiología y Salud Pública, Madrid, Spain

^51^Channing Division of Network Medicine, Department of Medicine, Brigham and Women's Hospital/Harvard Medical School, Boston, MA, USA

^52^Department of Cancer Epidemiology, Moffitt Cancer Center, Tampa, FL, USA

^53^Division of Public Health Sciences, Fred Hutchinson Cancer Research Center, Seattle, WA, USA

^54^Department of Epidemiology, School of Public Health, University of Washington, Seattle, WA, USA

^55^International Hereditary Cancer Center, Department of Genetics and Pathology, Pomeranian Medical University, Szczecin, Poland

^56^Faculty of Health and Medical Sciences, University of Copenhagen, Copenhagen, Denmark

^57^Department of Clinical Biochemistry, Herlev and Gentofte Hospital, Copenhagen University Hospital, Herlev, Copenhagen, Denmark

^58^Division of Clinical Epidemiology and Aging Research, German Cancer Research Center, Heidelberg, Germany

^59^German Cancer Consortium, German Cancer Research Center Heidelberg, Germany

^60^Division of Preventive Oncology, German Cancer Research Center and National Center for Tumor Diseases, Heidelberg, Germany

^61^Humangenetik Tuebingen, Tuebingen, Germany

^62^University of Texas M.D. Anderson Cancer Center, Department of Genitourinary Medical Oncology, Houston, TX, USA

^63^Departments of Epidemiology & Population Health and of Medicine, Division of Oncology, Stanford Cancer Institute, Stanford University School of Medicine, Stanford, CA, USA

^64^Department of Genetics, Portuguese Oncology Institute of Porto, Porto, Portugal

^65^Biomedical Sciences Institute, University of Porto, Porto, Portugal

^66^Cancer Genetics Group, IPO-Porto Research Center, Portuguese Oncology Institute of Porto, Porto, Portugal

^67^Department of Population Sciences, Beckman Research Institute of City of Hope, Duarte, CA, USA

^8^Ghent University, Faculty of Medicine and Health Sciences, Basic Medical Sciences, , Ghent, Belgium

^69^Department of Surgery, Faculty of Medicine, University of Malaya, Kuala Lumpur, Malaysia

^70^Department of Urology, University of Washington, Seattle, WA, USA

^71^Institute of Human Genetics, University Medical Center Hamburg-Eppendorf, Hamburg, Germany

^72^Molecular Medicine Center, Department of Medical Chemistry and Biochemistry, Medical University of Sofia, Sofia, Bulgaria

^73^Department of Oncology, Cross Cancer Institute, University of Alberta, Edmonton, AB, Canada

^74^Division of Radiation Oncology, Cross Cancer Institute, Edmonton, AB, Canada

^75^Molecular Endocrinology Laboratory, Department of Cellular and Molecular Medicine, KU Leuven, Leuven, Belgium

^76^Division of Cancer Sciences, Manchester Cancer Research Centre, Faculty of Biology, Medicine and Health, Manchester Academic Health Science Centre, NIHR Manchester Biomedical Research Centre, Health Innovation Manchester, University of Manchester, Manchester, UK

^77^University of Surrey, Guildford, UK

^78^Genetic Oncology Unit, Complexo Hospitalario Universitario de Vigo, Instituto de Investigación Biomédica Galicia Sur, Vigo (Pontevedra), Spain

^79^Department of Urology, Erasmus University Medical Center, Rotterdam, The Netherlands

^80^Exposome and Heredity, Faculté de Médecine, Université Paris-Saclay, Villejuif, France

^81^Clinical Gerontology Unit, University of Cambridge, Cambridge, UK

^82^Division of Epidemiology, Department of Internal Medicine, University of Utah School of Medicine, Salt Lake City, UT, USA

^83^George E. Wahlen Department of Veterans Affairs Medical Center, Salt Lake City, UT, USA

^84^Department of Laboratory Medicine and Pathology, Mayo Clinic, Rochester, MN, USA

^85^Nuffield Department of Population Health, University of Oxford, Oxford, UK

^86^Program in Genetic Epidemiology and Statistical Genetics, Department of Epidemiology, Harvard School of Public Health, Boston, MA, USA

^87^Division of Epidemiology, Department of Medicine, Vanderbilt University Medical Center, Nashville, TN, USA

^88^International Epidemiology Institute, Rockville, MD, USA

^89^Department of Epidemiology and Biostatistics, School of Public Health, Imperial College London, London, UK.

Funding and acknowledgements information for the PRACTICAL consortium, CRUK, BPC3, CAPS and PEGASUS are in the Supplementary material, available as Supplementary data at *IJE* online.

## Ethics approval

Each individual study obtained ethical approval, therefore additional ethical approval for this study was not required.

## Supplementary Material

dyac124_Supplementary_Data

## Data Availability

Studies pooled by the EHNBPCCG are not owned by the writing group and so are not available from this consortium. Individual studies may be contacted to request access to their data. PRACTICAL consortium data are available upon request, see [http://practical.icr.ac.uk/blog/] for further details.
