# Correction to: Circulating insulin-like growth factors and risks of overall, aggressive and early-onset prostate cancer: a collaborative analysis of 20 prospective studies and Mendelian randomization analysis

**DOI:** 10.1093/ije/dyaf094

**Published:** 2025-06-09

This is a correction to: Eleanor L Watts et al., Circulating insulin-like growth factors and risks of overall, aggressive and early-onset prostate cancer: a collaborative analysis of 20 prospective studies and Mendelian randomization analysis, *International Journal of Epidemiology*, Volume 52, Issue 1, February 2023, Pages 71–86, https://doi.org/10.1093/ije/dyac124

In the originally published version of this manuscript, there was a typographic error in author Anja Olsen’s name. This error has been corrected.

