# Supplementary material for: Circulating insulin-like growth factors and risks of overall, aggressive and early-onset prostate cancer: a collaborative analysis of 20 prospective studies and Mendelian randomization analysis
Source: Int J Epidemiol. 2022 Jun 21;52(1):71–86. doi: 10.1093/ije/dyac124 (PMC9908067; doi:10.1093/ije/dyac124)
Supplement: dyac124_Supplementary_Data [file dyac124_supplementary_data.zip › dyac124_Supplementary_Data/ije-2021-10-1479-File006.docx]

**Circulating insulin-like growth factors and risks of overall, aggressive and early-onset prostate cancer: a collaborative analysis of 20 prospective studies and Mendelian randomization analysis – Supplementary Tables and Figures**

| **Table of contents** | **Page** |
| --- | --- |
| **Supplementary Methods** | 3-5 |
| **Supplementary Tables** |  |
| Supplementary Table S1: Sample populations, recruitment, and case ascertainment methods | 6-7 |
| Supplementary Table S2: Criteria used by individual studies to match case patients and control subjects | 8-9 |
| Supplementary Table S3: Assay methods and geometric mean hormone concentrations by study | 10-12 |
| Supplementary Table S4: Prostate cancer characteristics in participating EHNBPCCG studies | 13 |
| Supplementary Table S5: Partial correlation coefficients of circulating biomarkers in prostate cancer controls | 14 |
| Supplementary Table S6: Colocalization analysis of IGF-I and prostate cancer | 15 |
| Supplementary Table S7: Risks of overall, aggressive and early-onset prostate cancer per 80% tile increment in biomarker concentrations in the EHNBPCCG studies | 16 |
| Supplementary Table S8: Risks of overall, aggressive and early-onset prostate cancer per study-specific 1 SD increment in biomarker concentrations with mutual adjustment, among cases and their matched controls in EHNBPCCG studies | 17-18 |
| Supplementary Table S9: Risks of overall prostate cancer in relation to biomarkers, stratified by other study-specific median biomarker concentrations in EHNBPCCG studies | 19 |
| Supplementary Table S10: Risks of aggressive* prostate cancer in relation to biomarkers, stratified by other study-specific median biomarker concentrations in EHNBPCCG studies | 20 |
| Supplementary Table S11: Association of IGF-I SNPs used in 2-sample Mendelian randomization analyses with prostate cancer | 21-24 |
| Supplementary Table S12: Outlier SNPs identified by MR-PRESSO* | 25 |
| Supplementary Table S13: Mendelian randomization estimates for the associations of genetically predicted circulating IGF-I concentrations risks of overall, aggressive and early-onset prostate cancer after Steiger filtering | 26 |
| **Supplementary Figures** |  |
| Supplementary Figure S1: Colocalization of circulating IGF-I concentrations and overall prostate cancer risk | 27 |
| Supplementary Figure S2: Z-Z plot of IGF-I and overall prostate cancer risk | 28 |
| Supplementary Figure S3: Odds ratio (95% CIs) for overall prostate cancer per study-specific 1 SD increment of IGF-II concentration by subgroup | 29 |
| Supplementary Figure S4: Odds ratio (95% CIs) for aggressive* prostate cancer per study-specific 1 SD increment of IGF-II concentration by subgroup | 30 |
| Supplementary Figure S5: Odds ratio (95% CIs) for overall prostate cancer per study-specific 1 SD increment of IGFBP-1 concentration by subgroup | 31 |
| Supplementary Figure S6: Odds ratio (95% CIs) for aggressive* prostate cancer per study-specific 1 SD increment of IGFBP-1 concentration by subgroup | 32 |
| Supplementary Figure S7: Odds ratio (95% CIs) for overall prostate cancer per study-specific 1 SD increment of IGFBP-2 concentration by subgroup | 33 |
| Supplementary Figure S8: Odds ratio (95% CIs) for aggressive prostate cancer* per study-specific 1 SD increment of IGFBP-2 concentration by subgroup | 34 |
| Supplementary Figure S9: Odds ratio (95% CIs) for overall prostate cancer per study-specific 1 SD increment of IGFBP-3 concentration by subgroup | 35 |
| Supplementary Figure S10: Odds ratio (95% CIs) for aggressive prostate cancer* per study-specific 1 SD increment of IGFBP-3 concentration by subgroup | 36 |
| Supplementary Figure S11: Odds ratios (95% confidence intervals) for prostate cancer associated with a 1 SD increment in IGF-I by study | 37 |
| Supplementary Figure S12: Odds ratios (95% confidence intervals) for aggressive* prostate cancer associated with a 1 SD increment in IGF-I by study | 38 |
| Supplementary Figure S13: Odds ratios (95% confidence intervals) for overall prostate cancer associated with a 1 SD increment in IGF-II by study | 39 |
| Supplementary Figure S14: Odds ratios (95% confidence intervals) for aggressive* prostate cancer associated with a 1 SD increment in IGF-II by study | 40 |
| Supplementary Figure S15: Odds ratios (95% confidence intervals) for overall prostate cancer associated with a 1 SD increment in IGFBP-1 by study | 41 |
| Supplementary Figure S16: Odds ratios (95% confidence intervals) for aggressive* prostate cancer associated with a 1 SD increment in IGFBP-1 by study | 42 |
| Supplementary Figure S17: Odds ratios (95% confidence intervals) for overall prostate cancer associated with a 1 SD increment in IGFBP-2 by study | 43 |
| Supplementary Figure S18: Odds ratios (95% confidence intervals) for aggressive* prostate cancer associated with a 1 SD increment in IGFBP-2 by study | 44 |
| Supplementary Figure S19: Odds ratios (95% confidence intervals) for prostate cancer associated with a 1 SD increment in IGFBP-3 by study | 45 |
| Supplementary Figure S20: Odds ratios (95% confidence intervals) for aggressive* prostate cancer associated with a 1 SD increment in IGFBP-3 by study | 46 |
| Supplementary Figure S21: Risks of overall and aggressive* prostate cancer by study-specific fifths of biomarker concentrations and 1 SD increment in the unadjusted model | 47 |
| Supplementary Figure S22: Risks of aggressive* prostate cancer by study-specific tenths of biomarker concentrations and 90%tile increment | 48 |
| Supplementary Figure S23: Forest plot of single SNP associations with overall prostate cancer | 49 |
| Supplementary Figure S24: Forest plot of single SNP associations with aggressive* prostate cancer | 50 |
| Supplementary Figure S25: Forest plot of single SNP associations with early-onset* prostate cancer | 51 |
| Supplementary Figure S26: Leave-one-out plot of genetic associations with overall prostate cancer | 52 |
| Supplementary Figure S27: Leave-one-out plot of genetic associations with aggressive* prostate cancer | 53 |
| Supplementary Figure S28: Leave-one-out plot of genetic associations with early-onset* prostate cancer | 54 |
| Supplementary Figure S29: Scatterplot of genetic associations with IGF-I against genetic associations with: A) Overall prostate cancer; B) Aggressive prostate cancer; C) Early-onset prostate cancer | 55 |
| Supplementary Figure S30: Traits associated with genetically instrumented IGF-I | 56 |
|  |  |
| **References** | 57-59 |

**Supplementary methods**

1. *Endogenous Hormones, Nutritional Biomarkers and Prostate Cancer Collaborative Group*

Data collection

Principal investigators were invited to join this collaborative group if they had published or unpublished studies on prostate cancer risk and endogenous sex hormone and/or nutritional biomarker concentrations that had been determined from blood samples collected before diagnosis. Studies were identified by literature searches of computerised bibliographic systems, including PubMed, Web of Science, Cochrane Library, and CancerLit, and through discussions with colleagues(3).

Individual participant data were available from 20 prospective studies by dataset closure on 1^st^ December 2019. We included all prospective studies with IGF-I, IGF-II, IGFBP-1,-2,-3 measurements. In total, 17,009 cases and 37,243 controls, including 2,332 aggressive, and 607 early-onset prostate cancer cases were analysed from the following studies: Alpha-Tocopherol, Beta-Carotene Cancer Prevention Study (ATBC)(4), Baltimore Longitudinal Study of Aging (BLSA)(5), British United Provident Association Study (BUPA)(6), Child Health and Development Studies (CHDS)(7), Cardiovascular Health Study (CHS)(8), CLUE I (9), European Prospective Investigation into Cancer and Nutrition (EPIC)(10, 11), European Randomized Study of Screening for Prostate Cancer (ERSPC)(12), Health In Men Study (HIMS)(13, 14), Health Professionals Follow-up Study (HPFS)(15-17), Japan Collaborative Cohort Study (JACC)(18), Kaiser Permanente Medical Care Program (KPMCP)(19), Melbourne Collaborative Cohort Study (MCCS)(20), Multiethnic Cohort (MEC)(21), Northern Sweden Health and Disease Cohort (NSHDC)(22-24), Prostate Cancer Prevention Trial (PCPT)(25), Physicians’ Health Study (PHS)(26-28), Prostate, Lung, Colorectal and Ovarian Cancer Screening Trial (PLCO)(29-31), **SUpplémentation en VItamines et Minéraux AntioXydants** (SU.VI.Max)(32), and UK Biobank(33-35).

The characteristics of these studies in the collaborative analyses are found in their original publications and are summarised in Supplementary Table 1. Most of the studies are case-control studies nested within traditional prospective cohort studies, with some variation in the case mix of these studies according to the prevalence of prostate-specific antigen (PSA) testing within that population during follow-up. For example, there is a generally higher proportion of early stage and low-grade cases in studies from the USA, where there has been relatively high levels of PSA-testing since the mid-1990s, than in studies in European populations where PSA-testing has only more recently started to become common. Three studies (ERPSC, PCPT and PLCO) are observational investigations using data from trials that included organised screening for prostate cancer. In these trials, men with a raised PSA or abnormal digital rectal examination at recruitment-screening were excluded, and the eligible cases were diagnosed during subsequent follow-up (for ERSPC and PCPT the majority being diagnosed at the end of the study, 4 and 7 years after recruitment, respectively), with the majority of cases being detected either through PSA-screening (ERSPC and PLCO) or by routine end of study biopsy (PCPT).

Of the randomised trials, data were available for participants in both the intervention and placebo arm in ATBC and PHS. Assay data were only available in the screening arm of the PLCO and ERSPC trials, and the placebo arm in PCPT (as the intervention arm was designed to alter prostate hormone concentrations)(25, 36). Four studies were cohort or case-cohort analyses (BLSA, HIMS, MCCS and UK Biobank), in which hormone concentrations had been measured from stored serum from all, or a subset, of the cohort. To apply a consistent statistical approach across all studies, the cases from the case-cohort studies were matched to up to four participants who were free of prostate cancer at the age at diagnosis of the case on the basis of our minimal matching criteria (Supplementary Table 2). Some studies used density sample, meaning that an individual could appear more than once in a data file.

Principal investigators were asked to provide data on prostate cancer case or non-case status, and if applicable, a matched-set identifier, as well as participant and tumor characteristics, and prostate cancer mortality. Individual participant data were also contributed for participant characteristics including age, height, weight, smoking status, alcohol consumption, marital status, education achievement, racial/ethnic group, diabetes status, and PSA and other biomarker concentrations at blood collection (where available). Endogenous hormone concentrations requested included IGF-I, IGF-II, IGFBP-1, IGFBP-2, IGFBP-3, testosterone, SHBG, and a number of other selected hormones and nutritional biomarkers. Collaborators also provided information on assay and time and date of blood collection. Information was requested about prostate cancer included date of diagnosis and stage and grade of disease, as well as prostate cancer mortality. Men were excluded from the analyses if data were missing for date of birth, blood collection, or diagnosis (for cases) or if they were known to be receiving androgen therapy at blood collection.

Data Processing

Disease definitions were as defined by the PRACTICAL consortium(37, 38). Aggressive prostate cancer was categorised as “yes” for any of the following: disease metastases at diagnosis (M1), Gleason score 8+ (or equivalent), prostate cancer death (defined as death from prostate cancer), or prostate-specific antigen (PSA) >100 ng/mL.

Prostate cancer was defined as low grade if the Gleason score was <7 or equivalent (i.e., extent of differentiation good, moderate), medium grade if Gleason score was 7 (i.e. poorly differentiated), and high grade if the Gleason score was ≥8 or equivalent (i.e., undifferentiated), or grade “unknown” otherwise.

Cases were defined as being early stage if they were tumor–node–metastasis (TNM) stage <T2 with no reported lymph node involvement or metastases or stage I; other localized stage if they were TNM stage T2 with no reported lymph node involvement or metastases, stage II, or equivalent (i.e., a tumor that does not extend beyond the prostate capsule); advanced stage if they were TNM stage T3 or T4 and/or N1+ and/or M1, stage III–IV, or equivalent (i.e., a tumor extending beyond the prostate capsule and/or lymph node involvement and/or distant metastases); or stage unknown.

Early-onset prostate cancer was defined as aged ≤55 years at diagnosis. Disease subtype definitions were the same for observational and genetic analyses.

Statistical analyses

Conditional logistic regression was used to calculate prostate cancer risk by concentrations of IGF-I, IGF-II, IGFBP-1, IGFBP-2, and IGFBP-3. Analyses were conditioned on the study-specific matching variables and adjusted for age at blood collection (continuous), BMI (<25, 25-27.4, 27.5-29.9, 30+ kg/m^2^, unknown (1.9%)), height (<171, 171-175, 176-180, 180+ cm, unknown (1.9%)), smoking status (never, current, previous, unknown (1.7%)), usual alcohol consumption (none, 1-9, 10-19, 20-39, 40+ g/day, unknown (10.1%)), racial/ethnic group (white, black, East Asian, other, unknown (2.8%)), education status (<secondary/high school, secondary/high school, university+, unknown (17.9%)), married/cohabiting (yes, no, unknown (65.4%)), diabetes status (yes, no, unknown (7.2%)).

To account for any systematic differences between the studies in assay methods and blood sample types, biomarkers were standardised by study and entered into the model as a continuous variable, so each increment represents a 1 study-specific standard deviation increase in biomarker concentration. In categorical analyses, biomarkers were categorised into study-specific fifths with cut-points determined in the controls(39).

Further analyses

Tests for heterogeneity for case-defined factors were obtained by fitting separate models for each subgroup and assuming independence of the ORs using a method analogous to a meta-analysis. Tests for heterogeneity for non-case defined factors were assessed with a χ^2^-test of interaction between subgroup and the continuous exposure variable. For associations with overall prostate cancer diagnosis, subgroup categories were defined as follows: aggressive disease (yes, no), age at diagnosis (≤55, 56+ years), prostate cancer death (yes), stage (localized, other localized, advanced), grade (low, medium, high), time to diagnosis (<1, 1-2, 3-6, 7-9, 10+ years), year of diagnosis (pre 1990, 1990-1994, 1995 onwards), age at blood draw (≤55, 55-59, 60-64, 65-69, 70+ years), BMI (<25, 25-29.9, 30+ kg/m^2^), smoking status (never, ex, current), alcohol consumption (none, 1-9, 10+ g ethanol/day), PSA at blood collection (<2, 2-2.9, 3+ ng/mL), time of blood collection (morning, afternoon), racial/ethnic group (white, other), education status (no degree, degree), currently married/cohabiting (yes, no), diabetes status (yes, no), overnight fast (no, yes).

For aggressive prostate cancer diagnosis, subgroups were: age at diagnosis (≤55, 56+ years), prostate cancer death (yes), stage (localized/other localized, advanced), grade (low/medium, high), time to diagnosis (<1, 1-4, 5+ years), year of diagnosis (pre 1990, 1990-1994, 1995 onwards), age at blood draw (<60, 60-69, 70+ years), BMI (<25, 25-29.9, 30+ kg/m^2^), smoking status (never, ex, current), alcohol consumption (none, 1-9; 10+ g ethanol/day), PSA at blood collection (<2, 2-2.9, 3+ ng/mL), time of blood collection (morning, afternoon), racial/ethnic group (white, other), education status (no degree, degree), currently married/cohabiting (yes, no), diabetes status (yes, no), overnight fast (no, yes). Subgroups were defined *a priori* based on the availability of data and previous analyses using this dataset(40, 41).

For each biomarker, heterogeneity in linear trends of the biomarkers and aggressive and overall prostate cancer between studies was assessed by comparing the χ^2^ values for models with and without a (study) × (linear trend) interaction term. This was tested across between all studies as well as in studies which included organised screening in their study design (ERPSC, PCPT and PLCO).

We also investigated unadjusted matched associations, associations in study-specific tenths. For comparison with previous analyses using the EHNBPCCG, estimates for trend were also defined using the study-specific fifths of the biomarker concentrations scored as 0, 0.25, 0.5, 0.75, and 1 and entered into the model as a continuous variable, therefore a unit increase in this variable can be taken to represent an 80^th^ percentile increase in the biomarker study-specific concentration.

Associations with prostate cancer were also examined following mutual adjustment for the other analytes (IGF-I, IGF-II, IGFBP-1, IGFBP-2, IGFBP-3, free and total testosterone and sex-hormone-binding globulin), which were standardised by study (continuous). We additionally tested for interaction between the biomarkers by study-specific median concentrations, using a χ^2^-test of interaction. Stratified analyses and associations in tenths were not investigated for early-onset disease due to the limited number of cases.

1. *Mendelian randomization analysis*

Genetic instruments for hormone concentrations

Single nucleotide polymorphisms (SNPs) associated with circulating IGF-I were identified from a publicly available GWAS from 158,444 male UK Biobank participants of white British ancestry (*P*<5 x 10^-8^ significance threshold)(42). For this GWAS, investigators adjusted for 20 principal components, age and age^2^(42). UK Biobank genotyping details are reported elsewhere(43). To ensure SNPs were independent, we pruned SNPs by a linkage disequilibrium (LD) threshold of r^2^<0.001, based on the lowest p-value.

Genetic associations with prostate cancer

SNP associations for prostate cancer were obtained from fixed-effects meta-analyses based on individuals of white European ancestry in the PRACTICAL (Prostate Cancer Association Group to Investigate Cancer-Associated Alterations in the Genome) and GAME-ON/ELLIPSE consortia (Genetic Associations and Mechanisms in Oncology, Elucidating Loci Involved in Prostate Cancer Susceptibility)(37, 38), these consortia do not currently include UK Biobank data. Individual studies included in these consortia are detailed in Conti *et al*(44) and Schumacher *et al*(37). Associations with overall prostate cancer risk were generated from 85,554 prostate cancer cases and 91,972 controls(44) (accessed via dbGaP with project ID: 31553), with aggressive from 15,167 cases and 58,308 controls, and with early-onset disease from 6,988 cases and 44,256 controls(37, 38).

Statistical analysis

We used a 2-sample MR approach to estimate IGF-I associations with overall, aggressive and early-onset prostate cancer risk, using UK Biobank as our source of genetic instruments for IGF-I and PRACTICAL for genetic outcome analyses. For the 2-sample harmonization process, SNPs which did not have corresponding alleles and palindromic SNPs with minor allele frequency >0.42 were removed, where palindromic SNPs were inferable, strands were aligned(45).

The MR estimation for IGF-I was conducted using the multiplicative random effects inverse-variance weighted (IVW) method(46). We additionally calculated Cochran’s Q statistic for between SNP-heterogeneity effects, the I^2^ statistic to assess measurement error in SNP-exposure associations(47) and the F-statistic to assess instrument strength(48, 49), Cochran’s Q statistic for heterogeneity between the MR estimates for each SNP(50), and PhenoScanner was used to assess pleiotropy of the genetic instruments(1, 2). As sensitivity analyses, we used the MR residual sum and outlier (MR-PRESSO), MR robust adjusted profile score (MR-RAPS) and leave-one out analyses to investigate the role of SNP outliers(51). To assess pleiotropy, we used the weighted median, MR-Egger and the MR-Egger intercept(52-54). We also used the contamination mixture method, which assumes a normal distribution of valid instruments around the true causal value, and invalid instruments are normally distributed around zero in order to account for potentially pleiotropic variants(55). To rule-out reverse causality, analyses were repeated after applying Steiger filtering which excludes variants with larger effects on prostate cancer risk than on IGF-I(54).

The associations of the IGF-I *cis*-SNP, defined as the lead SNP on the biomarker gene coding region identified from the exposure datasets, with prostate cancer were investigated using the Wald ratio. This *cis*-SNP is less likely to be affected by horizontal pleiotropy than *trans-*­SNPs(56).

1. *Colocalization analysis*

Colocalization was used to investigate whether the associations of variation in the *IGF1* gene region with both circulating IGF-I concentration and prostate cancer risk share the same genetic signal or whether the associations identified by our MR analysis may be confounded by linkage disequilibrium(57). This technique tests five hypotheses within this region:

1. H0: no causal variants from either trait
2. H1: only a causal association with the exposure
3. H2: only a causal association with the outcome
4. H3: distinct causal SNPs for the outcome and the exposure
5. H4: a shared causal variant

Posterior probabilities were estimated using the UK Biobank and PRACTICAL datasets(42, 44), using the *coloc* R package(57). Analyses were conducted for a 75-kb region centered on the lead IGF-I *cis-*SNP (rs5742653). Colocalization was assessed using three approaches: conventional colocalization(57), which tests for the presence of a single shared genetic association signal, as well as the Sum of Single Effects (SuSiE) regression framework(58) and conditional iterative colocalization(59), the latter two methods allow for the possibility of multiple independent (but partially correlated) causal variants in proximity(60). We used the priors (p1=1E-3, p2=1E-4, p12=1E-5), which approximately equates to an 80% prior belief that there is only a signal in the IGF-I GWAS and a 0.001% prior belief in favour of colocalization. We created colocalization plots using LocusCompareR(61) and a z-z locus plot(62). We considered a posterior probability (PP4) of >0.7 as being consistent with evidence of colocalization between IGF-I and overall prostate cancer(57).

Statistical software

Observational analyses were performed using Stata version 14.1 (Stata Corporation, College Station, TX, USA). MR analyses were performed using the *TwoSampleMR* R package (version 0.4.2)(45) and figures were plotted in R version 3.6.3. All tests of significance were two-sided, and P-values <0.05 were considered statistically significant.

| **Supplementary Table S1: Sample populations, recruitment, and case ascertainment methods** | | | | | |
| --- | --- | --- | --- | --- | --- |
| Study | Sample population | Location | Recruitment period | Age at blood collection (years) | Prostate cancer ascertainment method |
| ATBC | Randomised trial of α-tocopherol and β-carotene among smokers | Finland | 1985-1988 | 50-69 | Cancer registry linkage, central review of medical records and specimens |
| BLSA | Prospective cohort study of the physiology of aging | USA | 1958-onward | 30-84 | Self-report with medical record review |
| BUPA | Prospective cohort study of health plan members | UK | 1975-1982 | 35-64 | Cancer registry and death registry linkage |
| CHDS | Prospective cohort study | USA | 1959-1966 | 20-55 | Cancer registry linkage |
| CHS | Prospective cohort study | USA | 1989-1993 | 65-89 | Cancer registry linkage or self-report with hospital discharge confirmation |
| CLUE | Prospective cohort study | USA | 1974-onward | 44-87 | Cancer registry linkage |
| EPIC | Prospective cohort study | Europe | 1991-2001 | 43-76 | Cancer registry linkage; health insurance record linkage; self-report with medical record review |
| ERPSC | Population-based randomized trial of PSA screening | Netherlands | 1991-2000 | 54-69 | Diagnosis as part of trial protocol |
| HIMS | Population-based cohort study | Australia | 1996-1999 (Blood collection 2001-2004) | 71-87 | Cancer registry linkage |
| HPFS | Cohort study of male dentists, optometrists, osteopathic physicians, podiatrists, pharmacists, and veterinarians | USA | 1986 | 46-87 | Self-report with medical record review |
| JACC | Prospective cohort study | Japan | 1988-1990 | 57-85 | Cancer registry linkage |
| KPMCP | Prospective cohort study of health plan members | USA | 1964-1970 | 60-85 | Cancer registry linkage |
| MCCS | Prospective cohort study | Australia | 1990-1994 | 40-72 | Cancer registry linkage |
| MEC | Prospective cohort study | USA | 1993-1996 (Blood collection 2001-2006) | 48-85 | Cancer registry linkage |
| NSHDC | Combination of a population-based intervention study to decrease cardiovascular disease and a population-based monitoring study of cardiovascular disease | Sweden | 1985-onward | 39-61 | Cancer registry linkage |
|  |  |  |  |  |  |
|  |  |  |  |  |  |
| PCPT | Randomised, placebo-controlled trial of finasteride and prostate cancer | USA | 1994-1997 | 55-83 | Diagnosed as part of trial protocol. Annual digital rectal examinations and PSA measurements. Biopsy if abnormal DRE or reported PSA level > 4.0 ng per. End-of-study prostate biopsy |
| PHS | Randomised trial of aspirin and β-carotene among physicians | USA | 1982-onward | 41-78 | Self-report with medical record review |
|  |  |  |  |  |  |
|  |  |  |  |  |  |
| PLCO | Randomised controlled multicenter trial for early detection of cancer of the prostate, lung, colorectum and ovary | USA | 1993-2001 | 55-74 | Medical and pathology record review after screening and self-report with medical record review |
| SU.VI.MAX | Population-based, double-blind, placebo controlled, randomized trial of supplementation with antioxidant vitamins and minerals (vitamin C, a-tocopherol, b-carotene, selenium,  and zinc) | France | 1994-1995 | 42-61 | Self-report with medical record review |
|  |  |  |  |  |  |
| UK Biobank | Prospective cohort study | UK | 2006-2010 | 40-69 | Cancer registry linkage |

Abbreviations: ATBC=The Alpha-Tocopherol, Beta-Carotene Cancer Prevention Study; BLSA= The Baltimore Longitudinal Study of Aging; BUPA=British United Provident Association; CHDS=Child Health and Development Studies; CHS=Cardiovascular Health Study; CLUE=Give Us a Clue to Cancer and Heart Disease; DRE= digital rectal exam; EPIC=European Prospective Investigation into Cancer and Nutrition; ERSPC=European Randomized study of Screening for Prostate Cancer; HIMS=Health In Men Study; HPFS*=* Health Professionals Follow-up Study; KPMCP=Kaiser Permanente Medical Care Program; JACC=Japan Collaborative Cohort Study; MCCS=Melbourne Collaborative Cohort Study; MEC= Multiethnic Cohort Study of Diet and Cancer; NSHDC=Northern Sweden Health and Disease Cohort; PCPT= Prostate Cancer Prevention Trial; PHS=Physicians' Health Study; PLCO= Prostate, Lung, Colorectal and Ovarian Cancer Screening Trial; PSA=prostate-specific antigen; SU.VI.MAX=Supplémentation en Vitamines et Minéraux Antioxydants

| **Supplementary Table S2: Criteria used by individual studies to match case patients and control subjects** | | | | | |
| --- | --- | --- | --- | --- | --- |
| Study | Case:control ratio | Age at recruitment | Date of recruitment | Time of blood draw | Other matching criteria |
| ATBC |  |  |  |  | Random selection of cases diagnosed with prostate cancer at least 5 years from blood draw. Controls were randomly selected from members of the cohort. |
| BLSA* | 1:1 | ±6 mo |  |  | Follow-up time |
| BUPA | 1:3 | ±1 y |  |  | Duration of storage of the serum sample (±1 year). Controls required to be alive and without a cancer notification at time of case selection |
| CHDS | 1:2 | ±1 y |  |  | Ethnicity |
| CHS | 1:1 | ±3 y | ±1 y (entry and blood draw) |  | Ethnicity. Frequency matched and control participants survived to same age as case patient. |
| CLUE | 1:2 | ±1 y | ±3 weeks |  | Ethnicity |
| EPIC phases 1-4 and EPIC Denmark | 1:1 except for Umea center (1:2) | ±6 mo |  | ±1 h | Recruitment center, time between blood draw and last food or drink consumption, follow-up time |
| EPIC- Norfolk | 1:2 | ±3 y | ±3 mo |  | Follow-up time |
| ERPSC | 1:1 | ±1 y |  |  | PSA level at first visit (<2, 2-3, 3-4 µg/L), postal code |
| HIMS* | 1:4 | ±1 y |  |  | ±1 y date of blood collection  Fasting status, diabetes, controls must be 'alive and at risk' beyond the case's date of diagnosis |
| HPFS | 1:1 | Year of birth ±1 y | Exact year | Midnight–9 am; 9 am–12 pm; 12 pm–4 pm; and 4 pm–midnight | PSA test before blood draw, season. control subjects had at least one PSA test after the date of blood draw |
| JACC | 1:3 | As close as possible |  |  | Recruitment area |
| KPMCP | 1:3 | Same age |  |  |  |
| MCCS* | 1:3 | ±5 y | ±2 y |  | Assay batch, country of birth |
| MEC | 1:2 | ±1 y | ±6 mo | ±2 h | Geographic site, ethnicity, fasting status (<6, 6-7, 8-9, 10+ hours) |
| NSHDC | 1:2 | ±6 mo | ±2 mo |  | County of residency |
| PCPT | 1:1 | As close as possible | As close as possible |  | PCPT treatment arm (placebo only). All non-whites controls were sampled and then backfilled with whites to achieve frequency matching on age and family history. Controls were required to have completed end of study biopsy procedure. |
| PHS | 1:2 | ±1 y |  |  | Had not had a total or partial prostatectomy and smoking status |
| PLCO | 1:1 | ±5 y | Exact year |  | Follow-up time |
| SU.VI.MAX | 1:4 | Same age |  |  | All men randomized in the study were eligible |
| UK Biobank* | 1:4 | ±18 mo | ±18 mo | ±2 h | No more than 2 hours difference in time since last meal, ethnicity |

*Used a case–cohort design that was subsequently converted into nested case–control design.

Abbreviations: ATBC=The Alpha-Tocopherol, Beta-Carotene Cancer Prevention Study; BLSA= The Baltimore Longitudinal Study of Aging; BUPA=British United Provident Association; CHDS=Child Health and Development Studies; CHS=Cardiovascular Health Study; CLUE=Give Us a Clue to Cancer and Heart Disease; EPIC=European Prospective Investigation into Cancer and Nutrition; ERSPC=European Randomized study of Screening for Prostate Cancer; HIMS=Health In Men Study; HPFS*=* Health Professionals Follow-up Study; KPMCP=Kaiser Permanente Medical Care Program; JACC= Japan Collaborative Cohort Study; MCCS=Melbourne Collaborative Cohort Study; MEC=Multiethnic Cohort Study of Diet and Cancer; NSHDC=Northern Sweden Health and Disease Cohort; PCPT= Prostate Cancer Prevention Trial; PHS=Physicians' Health Study; PLCO= Prostate, Lung, Colorectal and Ovarian Cancer Screening Trial; PSA=prostate-specific antigen; SU.VI.MAX=Supplémentation en Vitamines et Minéraux Antioxydants.

**Supplementary Table S3: Assay methods and geometric mean biomarker concentrations by study**

|  |  |  |  | IGF-I (nmol/L) | | |  | IGF-II (nmol/L) | | |
| --- | --- | --- | --- | --- | --- | --- | --- | --- | --- | --- |
| Study | Sample |  |  | Method | CV % | Mean (SD) |  | Method | CV % | Mean (SD) |
| ATBC | Serum | Case |  | ELISA | 6.6^§^ | 19(6.9) |  |  |  |  |
|  |  | Control |  |  |  | 19(6.7) |  |  |  |  |
| BLSA | Serum | Case |  | E RIA | 4.6-20^§^ | 19(5.9) |  | E RIA | 4.9-30^§^ | 33(21) |
|  |  | Control |  |  |  | 19(6.5) |  |  |  | 45(15) |
| BUPA | Serum | Case |  | ELISA | N/A | 17(7.5) |  | ELISA | N/A | 96(24) |
|  |  | Control |  |  |  | 17(7.2) |  |  |  | 93(23) |
| CHDS | Not published | Case |  | Not published | N/A | 31(13) |  |  |  |  |
|  |  | Control |  |  |  | 32(13) |  |  |  |  |
| CHS | EDTA plasma | Case |  | IRMA | 3.0-12.3^§^ | 21(12) |  |  |  |  |
|  |  | Control |  |  |  | 21(10) |  |  |  |  |
| CLUE | Serum | Case |  | ELISA | N/A | 16(6) |  | Not published | N/A | 67(17) |
|  |  | Control |  |  |  | 16(5.3) |  |  |  | 70(20) |
| EPIC Phase 1 | Serum | Case |  | ELISA | 3.0-13.7^§^ | 24(8.7) |  |  |  |  |
|  |  | Control |  |  |  | 23(9.6) |  |  |  |  |
| EPIC Phase 2 | Serum | Case |  | ELISA (IDS-iSYS Immuno-diagnostic Systems Ltd, Swedish samples) | 3.2-4.4^§^ | 21(5.4) |  | RIA | 7.8-12.2^§^ | 121(37) |
|  |  | Control |  |  |  | 20(5.2) |  |  |  | 118(38) |
| EPIC Phase 3/4 | Serum | Case |  | ECIA | 2.5-2.8^‡^ | 19(4.8) |  | ELISA | 3.0-5.45^§^ | 62(13) |
|  |  | Control |  |  |  | 19(4.8) |  |  |  | 61(13) |
| EPIC Denmark | Serum | Case |  | ELISA | 3.3-3.9^‡^ | 19(4.6) |  |  |  |  |
|  |  | Control |  |  |  | 18(4.4) |  |  |  |  |
| ERPSC | Serum | Case |  | IRMA | 3.4† | 18(6.9) |  |  |  |  |
|  |  | Control |  |  |  | 18(7) |  |  |  |  |
| HIMS | Plasma | Case |  | ELISA | 8.6-12.2‡ | 18(7) |  |  |  |  |
|  |  | Control |  |  |  | 18(7.5) |  |  |  |  |
| HPFS I | Plasma | Case |  | ELISA | <10% (batch 1998 to 2000, CV=13%)† | 25(7.6) |  |  |  |  |
|  |  | Control |  |  |  | 23(7.5) |  |  |  |  |
| HPFS II | Plasma | Case |  | ELISA |  | 29(8) |  |  |  |  |
|  |  | Control |  |  |  | 28(7.9) |  |  |  |  |
| JACC | Serum | Case |  | IRMA | 2.1-3.5† | 16(8.8) |  | IRMA | 2.7-4.4† | 73(16) |
|  |  | Control |  |  |  | 15(7.5) |  |  |  | 70(16) |
| KPMCP | Serum | Case |  | E RIA | - | 21(7.6) |  |  |  |  |
|  |  | Control |  |  |  | 22(6.8) |  |  |  |  |
| MCCS | Plasma | Case |  | ELISA | 11.1^†^ | 23(8.3) |  |  |  |  |
|  |  | Control |  |  |  | 23(8.8) |  |  |  |  |
| MEC | Serum | Case |  | ELISA | 2.1† | 25(8.2) |  | ELISA | 1.8† | 122(32) |
|  |  | Control |  |  |  | 24(7.3) |  |  |  | 119(32) |
| NSHDC | Plasma | Case |  | IRMA | 8.6-13.8^§^ | 29(10) |  |  |  |  |
|  |  | Control |  |  |  | 27(10) |  |  |  |  |
| PCPT | Serum | Case |  | ELISA | 5.3-7.1† | 28(8.7) |  | ELISA | 4.2-5.0† | 235(58) |
|  |  | Control |  |  |  | 27(8.2) |  |  |  | 231(58) |
| PHS | Plasma | Case |  | ELISA | 4.9-6.5† | 25(9.6) |  | ELISA | N/A | 69(16) |
|  |  | Control |  |  |  | 24(8.9) |  |  |  | 69(16) |
| PLCO | Serum | Case |  | ELISA | 9^§^ | 28(11) |  |  |  |  |
|  |  | Control |  |  |  | 27(11) |  |  |  |  |
| SU.VI.MAX | Plasma | Case |  | Chemiluminescence | 5.3* | 20(5.9) |  | IRMA | 6.8* | 146(29) |
|  |  | Control |  |  |  | 20(6.3) |  |  |  | 143(28) |
| UK Biobank | Serum | Case |  | ECIA | 5.3-6.2^§^ | 22(5.2) |  |  |  |  |
|  |  | Control |  |  |  | 21(5.4) |  |  |  |  |

Abbreviations: ATBC=The Alpha-Tocopherol, Beta-Carotene Cancer Prevention Study; BLSA= The Baltimore Longitudinal Study of Aging; BUPA=British United Provident Association; CARET =The Carotene and Retinol Efficacy Trial; CHDS=Child Health and Development Studies; CHS=Cardiovascular Health Study; CLUE=Give Us a Clue to Cancer and Heart Disease; CV=coefficient of variation; E RIA=extraction radioimmunoassay; ECIA= electrochemiluminescence immunoassay; ELISA=enzyme-linked immunosorbent assay; EPIC=European Prospective Investigation into Cancer and Nutrition; ERSPC=European Randomized study of Screening for Prostate Cancer; FMC= Finnish Mobile Clinic Health Examination Survey*;* HHS= Helsinki Heart Study; HIMS=Health In Men Study; HPFS*=* Health Professionals Follow-up Study; IA=immunoassay; IMF=immunofluorometry; IRMA= immunoradiometric assay; KPMCP=Kaiser Permanente Medical Care Program; JACC= Japan Collaborative Cohort Study; JPHC= Japan Public Health Center-based Prospective Study; JHCS= Japan-Hawaii Cancer Study; LC-MS/MS= Liquid chromatography-tandem mass spectrometry; MCCS=Melbourne Collaborative Cohort Study; MEC= Multiethnic Cohort Study of Diet and Cancer; MMAS=Massachusetts Male Aging Study; NBSBWG=Nordic Biological Specimen Biobank Working Group; NE RIA=non-extraction radioimmunoassay; NSHDC=Northern Sweden Health and Disease Cohort; PCPT= Prostate Cancer Prevention Trial; PHS=Physicians' Health Study; PLCO= Prostate, Lung, Colorectal and Ovarian Cancer Screening Trial; PSA=prostate-specific antigen; RBS=Rancho Bernardo Study; SD=standard deviation; SU.VI.MAX=Supplémentation en Vitamines et Minéraux Antioxydants.

* Not specified

† Intra-assay

‡ Inter-assay

§ Intra-and inter-assay range

**Supplementary Table S3: Assay methods and geometric mean biomarker concentrations by study (continued)**

|  |  |  |  | IGFBP-1 (nmol/L) | | |  | IGFBP-2 (nmol/L) | | |  | IGFBP-3 (nmol/L) | | |
| --- | --- | --- | --- | --- | --- | --- | --- | --- | --- | --- | --- | --- | --- | --- |
| Study | Sample |  |  | Method | CV % | Mean (SD) |  | Method | CV % | Mean (SD) |  | Method | CV % | Mean (SD) |
| ATBC | Serum | Case |  |  |  |  |  |  |  |  |  | ELISA | 7.3† | 88(26) |
|  |  | Control |  |  |  |  |  |  |  |  |  |  |  | 82(22) |
| BLSA | Serum | Case |  |  |  |  |  |  |  |  |  | NE RIA | 5.1-17^§^ | 104(56) |
|  |  | Control |  |  |  |  |  |  |  |  |  |  |  | 98(26) |
| BUPA | Serum | Case |  |  |  |  |  |  |  |  |  | ELISA | N/A | 103(27) |
|  |  | Control |  |  |  |  |  |  |  |  |  |  |  | 100(27) |
| CHS | EDTA plasma | Case |  |  |  |  |  |  |  |  |  | IRMA | 2.1-7.1^§^ | 109(32) |
|  |  | Control |  |  |  |  |  |  |  |  |  |  |  | 112(30) |
| CLUE | Serum | Case |  | Not published | N/A | 0.29(0.36) |  |  |  |  |  | ELISA | N/A | 36(10) |
|  |  | Control |  |  |  | 0.28(0.32) |  |  |  |  |  |  |  | 36(9) |
| EPIC Phase 1 | Serum | Case |  |  |  |  |  |  |  |  |  | ELISA | 5.3-9.4^§^ | 133(27) |
|  |  | Control |  |  |  |  |  |  |  |  |  |  |  | 132(28) |
| EPIC Phase 2 | Serum | Case |  | ELISA | 5.3-5.6^§^ | 0.42(0.65) |  | ELISA | 5-7.1^§^ | 13(7.4) |  | ELISA | 2.2-3.5^‡^ | 137(26) |
|  |  | Control |  |  |  | 0.44(0.55) |  |  |  | 13(8.1) |  |  |  | 135(25) |
| EPIC Phase 3/4 | Serum | Case |  | ELISA | 2.4-7.4^§^ | 0.07(0.06) |  | ELISA | 2.1-5.8^§^ | 3.3(2.2) |  | ELISA | 2.2-2.6‡ | 135(25) |
|  |  | Control |  |  |  | 0.07(0.06) |  |  |  | 3.5(2.4) |  |  |  | 135(24) |
| ERSPC | Serum | Case |  |  |  |  |  |  |  |  |  | IRMA | 3.9† | 126(24) |
|  |  | Control |  |  |  |  |  |  |  |  |  |  |  | 128(28) |
| HIMS | Plasma | Case |  | ELISA | 5.2-8.6‡ | 0.99(0.69) |  |  |  |  |  | ELISA | 4.4-16.8‡ | 133(32) |
|  |  | Control |  |  |  | 1.0(0.79) |  |  |  |  |  |  |  | 132(31) |
| HPFS I | Plasma | Case |  |  |  |  |  |  |  |  |  | ELISA | <10† | 122(34) |
|  |  | Control |  |  |  |  |  |  |  |  |  |  |  | 118(35) |
| HPFS II | Plasma | Case |  | Not published | N/A | 0.89(0.76) |  |  |  |  |  | Not published | N/A | 134(30) |
|  |  | Control |  |  |  | 0.94(0.82) |  |  |  |  |  |  |  | 131(31) |
| JACC | Serum | Case |  |  |  |  |  |  |  |  |  | IRMA | 3.1-4.2† | 98(30) |
|  |  | Control |  |  |  |  |  |  |  |  |  |  |  | 94(28) |
| MCCS | Plasma | Case |  |  |  |  |  |  |  |  |  | ELISA | 9.5† | 108(26) |
|  |  | Control |  |  |  |  |  |  |  |  |  |  |  | 107(26) |
| MEC | Serum | Case |  | ELISA | 2.2† | 1.0(0.86) |  |  |  |  |  | ELISA | 2.5† | 142(35) |
|  |  | Control |  |  |  | 1.0(0.83) |  |  |  |  |  |  |  | 138(37) |
| NSHDC | Plasma | Case |  | IRMA | 8.6-13.8^§^ | 1.5(0.94) |  | RIA | 2.5† | 21(12) |  | IRMA | 3.6-6.9^§^ | 85(19) |
|  |  | Control |  |  |  | 1.7(1.10) |  |  |  | 21(14) |  |  |  | 83(19) |
| PCPT | Serum | Case |  |  |  |  |  | ELISA | 5.5-8.9^§^ | 18(10) |  | ELISA | 4.2-4.8^§^ | 143(34) |
|  |  | Control |  |  |  |  |  |  |  | 16(9.9) |  |  |  | 141(34) |
| PHS | Plasma | Case |  | ELISA | 4.9-6.5† | 0.31(0.34) |  |  |  |  |  | ELISA | 7.0-9.0† | 111(27) |
|  |  | Control |  |  |  | 0.34(0.38) |  |  |  |  |  |  |  | 111(28) |
| PLCO | Serum | Case |  |  |  |  |  |  |  |  |  | ELISA | 9† | 160(38) |
|  |  | Control |  |  |  |  |  |  |  |  |  |  |  | 160(39) |
| SU.VI.MAX | Plasma | Case |  |  |  |  |  | RIA | 8.6* | 7.7(4.4) |  | Chemiluminescence | 6.3* | 142(26) |
|  |  | Control |  |  |  |  |  |  |  | 8(4.5) |  |  |  | 146(31) |

**Supplementary Table S4: Prostate cancer characteristics in participating EHNBPCCG studies**

^*^Aggressive disease was defined as Gleason Score 8+, death from prostate cancer, metastatic disease, or PSA>100. Non-aggressive disease is defined as Gleason score <8 and non-metastatic disease.

†Stage of disease was defined as follows: localized if TNM was T2 or lower with no reported lymph node involvement or metastases, stage II or lower, or equivalent (ie, a tumor that does not extend beyond the prostate capsule); advanced if TNM stage was T3 or T4 and/or N1+ and/or M1, stage III or IV, equivalent (ie, a tumor extending beyond the prostate capsule and/or lymph node involvement and/or distant metastases), or unknown.

‡Histological grade was categorised as low-intermediate grade (Gleason sum <8 or cases coded as well, moderately, or poorly differentiated), high grade (Gleason sum 8+ or cases coded as undifferentiated), or unknown.

^a^Percentage value is for those with known disease characteristics.

Abbreviations: ATBC=The Alpha-Tocopherol, Beta-Carotene Cancer Prevention Study; BLSA= The Baltimore Longitudinal Study of Aging; BUPA=British United Provident Association; CHDS=Child Health and Development Studies; CHS=Cardiovascular Health Study; CLUE=Give Us a Clue to Cancer and Heart Disease; EPIC=European Prospective Investigation into Cancer and Nutrition; ERSPC=European Randomized study of Screening for Prostate Cancer; HIMS=Health In Men Study; HPFS=Health Professionals Follow-up Study; KPMCP=Kaiser Permanente Medical Care Program; JACC= Japan Collaborative Cohort Study; MCCS=Melbourne Collaborative Cohort Study; MEC=Multiethnic Cohort Study of Diet and Cancer; NSHDC=Northern Sweden Health and Disease Cohort; PCPT= Prostate Cancer Prevention Trial; PHS=Physicians' Health Study; PLCO= Prostate, Lung, Colorectal and Ovarian Cancer Screening Trial; PSA=prostate-specific antigen; SU.VI.MAX=Supplémentation en Vitamines et Minéraux Antioxydants

| Study | Years blood collection to diagnosis, % | |  | Age at diagnosis, % | |  | Year of diagnosis, % | |  | Aggressive disease*, % | | |  | Disease stage†, % | | |  | Disease grade‡, % | | |  | Prostate cancer death, % |
| --- | --- | --- | --- | --- | --- | --- | --- | --- | --- | --- | --- | --- | --- | --- | --- | --- | --- | --- | --- | --- | --- | --- |
|  | <5 years | 5+ years |  | ≤55 years | >55 years |  | pre-1995 | 1995 onwards |  | No | Yes | Unknown^a^ |  | Localized | Advanced | Unknown^a^ |  | Low | High | Unknown^a^ |  | Yes |
| ATBC | 0 (0) | 100 (100) |  | 0 (0) | 100 (100) |  | 9 (9) | 91 (91) |  | 66 (67) | 32 (33) | 2 (2) |  | 69 (70) | 30 (30) | 1 (1) |  | 97 (100) | 0 (0) | 3 (3) |  | 30 (30) |
| BLSA | 9 (13) | 63 (88) |  | 2 (3) | 70 (97) |  | 63 (88) | 9 (13) |  | 23 (68) | 11 (32) | 38 (53) |  | 26 (72) | 10 (28) | 36 (50) |  | 45 (83) | 9 (17) | 18 (25) |  | 0 (0) |
| BUPA | 10 (7) | 131 (93) |  | 11 (8) | 130 (92) |  | 125 (89) | 16 (11) |  | 0 (0) | 1 (100) | 140 (99) |  | N/A | N/A | 141 (100) |  | N/A | N/A | 141 (100) |  | 16 (11) |
| CHDS | 0 (0) | 321 (100) |  | 21 (7) | 300 (93) |  | 141 (44) | 180 (56) |  | 248 (91) | 26 (9) | 47 (15) |  | 228 (80) | 57 (20) | 36 (11) |  | 298 (99) | 4 (1) | 19 (6) |  | 32 (10) |
| CHS | 143 (82) | 31 (18) |  | 0 (0) | 174 (100) |  | 108 (62) | 66 (38) |  | 102 (90) | 11 (10) | 61 (35) |  | 89 (76) | 28 (24) | 57 (33) |  | 141 (99) | 1 (1) | 32 (18) |  | 0 (0) |
| CLUE | 3 (10) | 27 (90) |  | 0 (0) | 30 (100) |  | 30 (100) | 0 (0) |  | 12 (52) | 11 (48) | 7 (23) |  | 15 (79) | 4 (21) | 11 (37) |  | 23 (92) | 2 (8) | 5 (17) |  | 9 (30) |
| EPIC Denmark | 4 (0) | 1,440 (100) |  | 0 (0) | 1,444 (100) |  | 0 (0) | 1,444 (100) |  | 355 (51) | 342 (49) | 747 (52) |  | 736 (64) | 415 (36) | 293 (20) |  | 434 (74) | 156 (26) | 854 (59) |  | 151 (10) |
| EPIC phase 1 | 478 (77) | 143 (23) |  | 38 (6) | 583 (94) |  | 6 (1) | 615 (99) |  | 248 (57) | 185 (43) | 188 (30) |  | 295 (68) | 137 (32) | 189 (30) |  | 412 (89) | 52 (11) | 157 (25) |  | 142 (23) |
| EPIC phase 2 | 254 (23) | 858 (77) |  | 55 (5) | 1,057 (95) |  | 4 (0) | 1,108 (100) |  | 470 (60) | 314 (40) | 328 (29) |  | 662 (74) | 229 (26) | 221 (20) |  | 648 (85) | 111 (15) | 353 (32) |  | 230 (21) |
| EPIC phs 3/4 | 17 (1) | 1,708 (99) |  | 65 (4) | 1,660 (96) |  | 0 (0) | 1,725 (100) |  | 720 (70) | 314 (30) | 691 (40) |  | 860 (75) | 287 (25) | 578 (34) |  | 1,034 (87) | 158 (13) | 533 (31) |  | 125 (7) |
| ERSPC | 196 (99) | 1 (1) |  | 0 (0) | 197 (100) |  | 0 (0) | 197 (100) |  | 183 (94) | 11 (6) | 3 (2) |  | 186 (94) | 11 (6) | 0 (0) |  | 189 (97) | 5 (3) | 3 (2) |  | 8 (4) |
| HIMS | 209 (66) | 108 (34) |  | 0 (0) | 317 (100) |  | 0 (0) | 317 (100) |  | 0 (0) | 35 (100) | 282 (89) |  | N/A | N/A | 317 (100) |  | N/A | N/A | 317 (100) |  | 35 (11) |
| HPFS hormone | 564 (83) | 118 (17) |  | 46 (7) | 636 (93) |  | 85 (12) | 597 (88) |  | 503 (83) | 102 (17) | 77 (11) |  | 608 (95) | 32 (5) | 42 (6) |  | 552 (91) | 54 (9) | 76 (11) |  | 80 (12) |
| HPFS markers | 36 (6) | 593 (94) |  | 17 (3) | 612 (97) |  | 1 (0) | 628 (100) |  | 495 (88) | 69 (12) | 65 (10) |  | 552 (97) | 18 (3) | 59 (9) |  | 518 (92) | 48 (8) | 63 (10) |  | 39 (6) |
| JACC | 18 (46) | 21 (54) |  | 0 (0) | 39 (100) |  | 22 (56) | 17 (44) |  | 0 (0) | 2 (100) | 37 (95) |  | N/A | N/A | 39 (100) |  | N/A | N/A | 39 (100) |  | 0 (0) |
| KPMCP | 11 (24) | 34 (76) |  | 0 (0) | 45 (100) |  | 45 (100) | 0 (0) |  | 6 (50) | 6 (50) | 33 (73) |  | 13 (62) | 8 (38) | 24 (53) |  | 10 (100) | 0 (0) | 35 (78) |  | 0 (0) |
| MCCS | 228 (41) | 325 (59) |  | 27 (5) | 526 (95) |  | 84 (15) | 469 (85) |  | 413 (76) | 127 (24) | 13 (2) |  | 494 (90) | 52 (10) | 7 (1) |  | 466 (86) | 73 (14) | 14 (3) |  | 109 (19) |
| MEC | 361 (94) | 25 (6) |  | 4 (1) | 382 (99) |  | 0 (0) | 386 (100) |  | 0 (0) | 42 (100) | 344 (89) |  | N/A | N/A | 386 (100) |  | 366 (100) | 1 (0) | 19 (5) |  | 40 (10) |
| NSHDC | 145 (52) | 136 (48) |  | 23 (8) | 258 (92) |  | 29 (10) | 252 (90) |  | 61 (56) | 48 (44) | 172 (61) |  | 226 (81) | 53 (19) | 2 (1) |  | 69 (97) | 2 (3) | 210 (75) |  | 0 (0) |
| PCPT | 235 (23) | 797 (77) |  | 0 (0) | 1,032 (100) |  | 4 (0) | 1,028 (100) |  | 931 (95) | 52 (5) | 49 (5) |  | 989 (98) | 17 (2) | 26 (3) |  | 959 (95) | 48 (5) | 25 (2) |  | 4 (0) |
| PHS | 107 (14) | 650 (86) |  | 25 (3) | 732 (97) |  | 575 (76) | 182 (24) |  | 503 (71) | 206 (29) | 38 (5) |  | 609 (85) | 107 (15) | 41 (5) |  | 657 (90) | 74 (10) | 26 (3) |  | 174 (23) |
| PLCO | 648 (89) | 79 (11) |  | 0 (0) | 728 (100) |  | 0 (0) | 728 (100) |  | 522 (89) | 66 (11) | 0 (0) |  | 636 (89) | 79 (11) | 0 (0) |  | 681 (95) | 35 (5) | 1 (0) |  | 30 (4) |
| SU.VI.MAX | 28 (28) | 72 (72) |  | 19 (19) | 81 (81) |  | 0 (0) | 100 (100) |  | 0 (0) | 12 (100) | 88 (88) |  | N/A | N/A | 100 (100) |  | 84 (89) | 10 (11) | 6 (6) |  | 4 (4) |
| SU.VI.MAX-D | 0 (0) | 80 (100) |  | 3 (4) | 77 (96) |  | 0 (0) | 80 (100) |  | 0 (0) | 8 (100) | 72 (90) |  | N/A | N/A | 80 (100) |  | 64 (89) | 8 (11) | 8 (10) |  | 0 (0) |
| UK Biobank | 3,700 (67) | 1,828 (33) |  | 253 (5) | 5,275 (95) |  | 0 (0) | 5,528 (100) |  | 0 (0) | 186 (100) | 5,342 (97) |  | N/A | N/A | 5,528 (100) |  | N/A | N/A | 5,528 (100) |  | 311 (5) |

**Supplementary Table S5: Partial correlation coefficients of circulating biomarkers in prostate cancer controls***

|  | IGF-I | IGF-II | IGFBP-1 | IGFBP-2 | IGFBP-3 | Total testosterone | SHBG | Free testosterone | PSA |
| --- | --- | --- | --- | --- | --- | --- | --- | --- | --- |
| IGF-I | 1 |  |  |  |  |  |  |  |  |
| IGF-II | 0.42^b^ | 1 |  |  |  |  |  |  |  |
| IGFBP-1 | 0.14^b^ | 0.43^b^ | 1 |  |  |  |  |  |  |
| IGFBP-2 | 0.24^b^ | 0.54^b^ | 0.71^b^ | 1 |  |  |  |  |  |
| IGFBP-3 | 0.50^b^ | 0.45^b^ | -0.13^b^ | -0.16^b^ | 1 |  |  |  |  |
| Total testosterone | 0.02^a^ | -0.21^b^ | 0.20^b^ | 0.05^a^ | -0.09^b^ | 1 |  |  |  |
| SHBG | -0.20^b^ | -0.27^b^ | 0.03 | 0.03 | -0.21^b^ | 0.49^b^ | 1 |  |  |
| Free testosterone | 0.17^b^ | -0.04 | 0.21^b^ | 0.03 | 0.07^b^ | 0.77^b^ | -0.17^b^ | 1 |  |
| PSA | -0.03^a^ | -0.004 | 0.04^a^ | 0.06^b^ | 0.01 | 0.08^b^ | -0.04^b^ | 0.11^b^ | 1 |

* Biomarkers were log-transformed and adjusted for age (5-year groups) and BMI (<25, 25-27.4, 27.5-29.9, 30+ kg/m^2^, unknown)

^a^ P <0.05

^b^ P <0.001

Abbreviations: IGF=insulin-like growth factor; IGFBP=insulin-like growth factor binding protein; PSA=prostate-specific antigen; SHBG=sex hormone binding globulin

**Supplementary Table S6: Colocalization analysis of IGF-I and overall prostate cancer**

| Method | N SNPs | Hit 1 | Hit 2 | PP H0 | PP H1 | PP H2 | PP H3 | PP H4 | Hit 1 r^2^ with rs5742653 | Hit 1 D’ with rs5742653 | Hit 2 r^2^ with rs5742653 | Hit 2 D’ with rs5742653 |
| --- | --- | --- | --- | --- | --- | --- | --- | --- | --- | --- | --- | --- |
| Single variant | 483 | rs5742653 | - | 1.90E-68 | 0.33 | 2.50E-68 | 0.44 | 0.23 | - | - | - | - |
| SuSiE | 483 | rs4764697 | - | 9.48E-270 | 1.17E-41 | 8.10E-231 | 6.83E-08 | >0.99 | 0.53 | 0.98 | - | - |
| SuSiE | 483 | rs5742671 | - | 0.00E+00 | 6.09E-06 | 0.00E+00 | 2.16E-02 | 0.98 | 0.63 | 1.00 | - | - |
| Conditional iterative | 483 | rs5742671 | rs7956547 | 0.00E+00 | 1.63E-03 | 0.00E+00 | 0.27 | 0.72 | 0.63 | 1.00 | 0.34 | 0.60 |

Gene regions were centered on the lead IGF-I cis-SNP (rs5742653). Posterior probability (PP) refers to the PP of each of the five possible hypotheses: H0: no causal variants from either trait; H1: only a causal association with the exposure; H2: only a causal association with the outcome; H3: distinct causal SNPs for the outcome and the exposure; H4: a shared causal variant.

Abbreviations: SNP: single nucleotide repeat polymorphism; SuSiE: Sum of Single Effects

**Supplementary Table S7:** **Risks of overall, aggressive and early-onset prostate cancer per 80% tile increment in biomarker concentrations in the EHNBPCCG studies**

|  | Overall prostate cancer | | |  | Aggressive prostate cancer* | | |  | Early-onset prostate cancer† | | |
| --- | --- | --- | --- | --- | --- | --- | --- | --- | --- | --- | --- |
|  | Cases/Controls | OR per 80%tile increment (95% CI) | *P*_trend_ |  | Cases/Controls | OR per 80%tile increment (95% CI) | *P*_trend_ |  | Cases/Controls | OR per 80%tile increment (95% CI) | *P*_trend_ |
| IGF-I | 17009/37243 | 1.27 (1.20, 1.34) | <0.0001 |  | 2332/3838 | 1.24 (1.05, 1.47) | 0.01 |  | 607/1561 | 1.54 (1.10, 2.14) | 0.01 |
| IGF-II | 4466/5566 | 1.18 (1.04, 1.34) | 0.01 |  | 701/778 | 0.74 (0.51, 1.06) | 0.10 |  | 150/236 | 1.34 (0.59, 3.02) | 0.48 |
| IGFBP-1 | 4491/5938 | 0.86 (0.76, 0.98) | 0.02 |  | 846/976 | 0.93 (0.69, 1.25) | 0.63 |  | 155/175 | 0.81 (0.36, 1.81) | 0.61 |
| IGFBP-2 | 3776/4210 | 1.06 (0.91, 1.23) | 0.46 |  | 617/680 | 1.00 (0.68, 1.47) | 0.99 |  | 136/205 | 1.29 (0.55, 3.03) | 0.56 |
| IGFBP-3 | 9113/12246 | 1.20 (1.10, 1.31) | <0.0001 |  | 1476/2030 | 1.01 (0.81, 1.26) | 0.94 |  | 306/480 | 1.38 (0.81, 2.35) | 0.24 |

Estimates are from logistic regression conditioned on the matching variables and adjusted for age, BMI, height, alcohol intake, smoking status, marital status, education status, racial/ethnic group, and diabetes status. The categorical variables representing the study-specific fifths of the biomarker concentrations was replaced with a continuous variable that was scored as 0, 0.25, 0.5, 0.75, and 1; because the mid-points of the lowest and highest fifths are the 10^th^ and 90^th^ percentiles of the study-specific biomarker concentrations, a unit increase in this variable can be taken to represent an 80 percentile increase in the biomarker study-specific concentration.

*Aggressive cancer defined as Gleason grade 8+, or prostate cancer death, or metastases or PSA>100 ng/mL.

†Early-onset defined as diagnosed ≤55 years.

Abbreviations: BMI=body mass index; CI=confidence interval; IGF=insulin-like growth factor; IGFBP=insulin-like growth factor binding protein; OR=odds ratio; PSA=prostate-specific antigen.

**Supplementary Table S8: Risks of overall, aggressive and early-onset prostate cancer per study-specific 1 SD increment in biomarker concentrations with mutual adjustment, among cases and their matched controls in EHNBPCCG studies**

|  | Overall prostate cancer | | |  | Aggressive prostate cancer* | | |
| --- | --- | --- | --- | --- | --- | --- | --- |
|  | Cases / Controls | OR (95% CI) | *P*_trend_ |  | Cases/ Controls | OR (95% CI) | *P*_trend_ |
| **IGF-I alone** | **17009/37243** | **1.09 (1.07, 1.11)** | **<0.0001** |  | **2332/3838** | **1.09 (1.03, 1.16)** | **0.01** |
| Adjusted for IGF-II | 4383/5481 | 1.07 (1.02, 1.13) | 0.004 |  | 675/752 | 1.05 (0.92, 1.20) | 0.45 |
| Adjusted for IGFBP-1 | 4423/5870 | 1.10 (1.05, 1.15) | 0.0001 |  | 821/950 | 1.04 (0.92, 1.17) | 0.54 |
| Adjusted for IGFBP-2 | 3694/4135 | 1.10 (1.04, 1.15) | 0.0004 |  | 591/654 | 1.08 (0.95, 1.23) | 0.26 |
| Adjusted for IGFBP-3 | 9117/12247 | 1.05 (1.01, 1.09) | 0.02 |  | 1475/2028 | 1.07 (0.96, 1.18) | 0.21 |
| Adjusted for total testosterone | 12395/31209 | 1.09 (1.07, 1.12) | <0.0001 |  | 1384/2712 | 1.09 (1.01, 1.18) | 0.02 |
| Adjusted for SHBG | 12101/31301 | 1.07 (1.04, 1.09) | <0.0001 |  | 1388/2658 | 1.08 (1.00, 1.17) | 0.04 |
| Adjusted for free testosterone | 11953/31109 | 1.09 (1.06, 1.11) | <0.0001 |  | 1349/2607 | 1.09 (1.01, 1.18) | 0.02 |
| **IGF-II alone** | **4466/5566** | **1.06 (1.02, 1.11)** | **0.008** |  | **701/778** | **0.91 (0.80, 1.04)** | **0.16** |
| Adjusted for IGF-I | 4408/5537 | 1.03 (0.98, 1.08) | 0.32 |  | 675/752 | 0.88 (0.76, 1.01) | 0.06 |
| Adjusted for IGFBP-1 | 2981/3337 | 1.08 (1.02, 1.14) | 0.01 |  | 610/634 | 0.88 (0.76, 1.02) | 0.09 |
| Adjusted for IGFBP-2 | 3632/3932 | 1.05 (1.00, 1.11) | 0.05 |  | 588/623 | 0.92 (0.79, 1.07) | 0.28 |
| Adjusted for IGFBP-3 | 4188/5217 | 0.97 (0.90, 1.04) | 0.42 |  | 604/675 | 0.85 (0.68, 1.06) | 0.15 |
| Adjusted for total testosterone | 3680/4072 | 1.06 (1.01, 1.11) | 0.03 |  | 525/548 | 0.87 (0.75, 1.01) | 0.07 |
| Adjusted for SHBG | 3738/4114 | 1.04 (0.99, 1.09) | 0.15 |  | 541/563 | 0.86 (0.74, 1.00) | 0.06 |
| Adjusted for free testosterone | 3678/4069 | 1.06 (1.01, 1.12) | 0.02 |  | 524/546 | 0.87 (0.75, 1.01) | 0.07 |
| **IGFBP-1 alone** | **4491/5938** | **0.95 (0.91, 0.99)** | **0.03** |  | **846/976** | **0.96 (0.87, 0.87)** | **0.43** |
| Adjusted for IGF-I | 4440/5907 | 0.97 (0.93, 1.01) | 0.16 |  | 821/950 | 0.97 (0.88, 1.07) | 0.55 |
| Adjusted for IGF-II | 2979/3328 | 0.97 (0.92, 1.03) | 0.30 |  | 610/634 | 0.95 (0.85, 1.07) | 0.43 |
| Adjusted for IGFBP-2 | 2635/2774 | 0.98 (0.92, 1.05) | 0.56 |  | 552/578 | 0.98 (0.86, 1.12) | 0.77 |
| Adjusted for IGFBP-3 | 4298/5738 | 0.96 (0.92, 1.01) | 0.13 |  | 758/888 | 0.93 (0.83, 1.04) | 0.20 |
| Adjusted for total testosterone | 3019/4301 | 0.98 (0.93, 1.03) | 0.45 |  | 532/628 | 0.96 (0.83, 1.10) | 0.54 |
| Adjusted for SHBG | 3078/4344 | 1.00 (0.95, 1.06) | 0.99 |  | 548/645 | 0.94 (0.81, 1.07) | 0.34 |
| Adjusted for free testosterone | 3017/4297 | 0.97 (0.92, 1.03) | 0.29 |  | 531/626 | 0.96 (0.84, 1.10) | 0.57 |
| **IGFBP-2 alone** | **3776/4210** | **0.98 (0.93, 1.03)** | **0.46** |  | **617/680** | **0.92 (0.81, 1.05)** | **0.21** |
| Adjusted for IGF-I | 3719/4178 | 1.01 (0.95, 1.06) | 0.82 |  | 591/654 | 0.95 (0.83, 1.08) | 0.41 |
| Adjusted for IGF-II | 3633/3933 | 1.00 (0.94, 1.05) | 0.90 |  | 588/623 | 0.91 (0.79, 1.04) | 0.17 |
| Adjusted for IGFBP-1 | 2637/2771 | 0.95 (0.88, 1.02) | 0.15 |  | 552/578 | 0.90 (0.78, 1.05) | 0.17 |
| Adjusted for IGFBP-3 | 3576/4005 | 1.02 (0.96, 1.08) | 0.50 |  | 528/591 | 0.97 (0.84, 1.12) | 0.69 |
| Adjusted for total testosterone | 3172/3179 | 1.01 (0.95, 1.08) | 0.65 |  | 443/443 | 0.94 (0.80, 1.11) | 0.49 |
| Adjusted for SHBG | 3229/3222 | 1.05 (0.99, 1.12) | 0.12 |  | 459/459 | 0.96 (0.81, 1.13) | 0.64 |
| Adjusted for free testosterone | 3170/3177 | 1.00 (0.95, 1.07) | 0.89 |  | 442/442 | 0.96 (0.82, 1.12) | 0.59 |
| **IGFBP-3 alone** | **9113/12246** | **1.08 (1.04, 1.11)** | **<0.0001** |  | **1476/2030** | **1.03 (0.95, 1.12)** | **0.42** |
| Adjusted for IGF-I | 9110/12238 | 1.04 (1.00, 1.09) | 0.03 |  | 1475/2028 | 0.99 (0.89, 1.10) | 0.84 |
| Adjusted for IGF-II | 4157/5190 | 1.09 (1.02, 1.18) | 0.02 |  | 604/675 | 1.07 (0.87, 1.31) | 0.52 |
| Adjusted for IGFBP-1 | 4275/5725 | 1.08 (1.03, 1.13) | 0.0007 |  | 758/888 | 0.95 (0.84, 1.07) | 0.41 |
| Adjusted for IGFBP-2 | 3545/3985 | 1.06 (1.01, 1.12) | 0.02 |  | 528/591 | 0.98 (0.86, 1.13) | 0.81 |
| Adjusted for total testosterone | 6597/8591 | 1.07 (1.03, 1.11) | 0.0002 |  | 1048/1458 | 1.01 (0.92, 1.11) | 0.84 |
| Adjusted for SHBG | 6680/8658 | 1.05 (1.01, 1.09) | 0.008 |  | 1075/1489 | 1.01 (0.91, 1.11) | 0.92 |
| Adjusted for free testosterone | 6566/8563 | 1.07 (1.03, 1.11) | 0.0002 |  | 1035/1444 | 1.00 (0.91, 1.10) | 0.97 |

Estimates are from logistic regression conditioned on the matching variables and adjusted for age, BMI, height, alcohol intake, smoking status, marital status, education status, racial/ethnic group, and diabetes status. Estimates were mutually adjusted for biomarkers per study-specific 1 SD entered as a continuous variable.

*Aggressive cancer defined as Gleason grade 8+, or prostate cancer death, or metastases or PSA>100 ng/mL.

†Early-onset defined as diagnosed ≤55 years.

Abbreviations: BMI=body mass index; CI=confidence interval; IGF=insulin-like growth factor; IGFBP=insulin-like growth factor binding protein; OR=odds ratio; SD=standard deviation; SHBG=sex hormone-binding globulin.

**Supplementary Table S8: Risks of overall, aggressive and early-onset prostate cancer per study-specific 1 SD increment in biomarker concentrations with mutual adjustment, among cases and their matched controls in EHNBPCCG studies (continued)**

|  | Early-onset prostate cancer† | | |
| --- | --- | --- | --- |
|  | Cases/ Controls | OR (95% CI) | *P*_trend_ |
| **IGF-I alone** | **607/1561** | **1.11 (1.00, 1.24)** | **0.05** |
| Adjusted for IGF-II | 148/234 | 0.94 (0.72, 1.23) | 0.66 |
| Adjusted for IGFBP-1 | 153/173 | 0.90 (0.67, 1.22) | 0.51 |
| Adjusted for IGFBP-2 | 134/203 | 0.94 (0.68, 1.29) | 0.69 |
| Adjusted for IGFBP-3 | 306/479 | 0.95 (0.77, 1.16) | 0.61 |
| Adjusted for total testosterone | 477/1328 | 1.12 (1.00, 1.26) | 0.06 |
| Adjusted for SHBG | 472/1332 | 1.06 (0.94, 1.20) | 0.34 |
| Adjusted for free testosterone | 458/1322 | 1.09 (0.96, 1.23) | 0.18 |
| **IGF-II alone** | **150/236** | **1.11 (0.84, 1.46)** | **0.47** |
| Adjusted for IGF-I | 148/235 | 1.14 (0.86, 1.50) | 0.37 |
| Adjusted for IGFBP-1 | 115/119 | 1.04 (0.74, 1.46) | 0.83 |
| Adjusted for IGFBP-2 | 121/178 | 1.14 (0.83, 1.55) | 0.43 |
| Adjusted for IGFBP-3 | 139/222 | 1.53 (0.95, 2.46) | 0.08 |
| Adjusted for total testosterone | 99/108 | 1.02 (0.70, 1.48) | 0.94 |
| Adjusted for SHBG | 106/109 | 1.06 (0.74, 1.52) | 0.75 |
| Adjusted for free testosterone | 99/107 | 1.03 (0.71, 1.50) | 0.89 |
| **IGFBP-1 alone** | **155/175** | **1.02 (0.75, 1.38)** | **0.89** |
| Adjusted for IGF-I | 153/174 | 0.99 (0.72, 1.37) | 0.96 |
| Adjusted for IGF-II | 115/119 | 1.12 (0.77, 1.64) | 0.55 |
| Adjusted for IGFBP-2 | 117/129 | 0.92 (0.61, 1.39) | 0.70 |
| Adjusted for IGFBP-3 | 147/167 | 1.01 (0.73, 1.38) | 0.96 |
| Adjusted for total testosterone | 101/110 | 1.06 (0.71, 1.56) | 0.79 |
| Adjusted for SHBG | 108/111 | 1.03 (0.70, 1.52) | 0.88 |
| Adjusted for free testosterone | 101/109 | 1.07 (0.72, 1.58) | 0.74 |
| **IGFBP-2 alone** | **136/205** | **1.08 (0.78, 1.49)** | **0.63** |
| Adjusted for IGF-I | 134/204 | 1.06 (0.76, 1.47) | 0.73 |
| Adjusted for IGF-II | 121/178 | 1.13 (0.80, 1.58) | 0.49 |
| Adjusted for IGFBP-1 | 117/129 | 1.27 (0.81, 1.97) | 0.30 |
| Adjusted for IGFBP-3 | 128/197 | 1.03 (0.74, 1.44) | 0.86 |
| Adjusted for total testosterone | 86/91 | 1.29 (0.76, 2.18) | 0.34 |
| Adjusted for SHBG | 93/92 | 1.17 (0.69, 1.96) | 0.56 |
| Adjusted for free testosterone | 86/90 | 1.35 (0.80, 2.28) | 0.26 |
| **IGFBP-3 alone** | **306/480** | **1.14 (0.94, 1.39)** | **0.18** |
| Adjusted for IGF-I | 306/479 | 1.18 (0.93, 1.48) | 0.17 |
| Adjusted for IGF-II | 139/222 | 0.69 (0.42, 1.14) | 0.15 |
| Adjusted for IGFBP-1 | 147/167 | 0.97 (0.71, 1.31) | 0.82 |
| Adjusted for IGFBP-2 | 128/197 | 0.94 (0.69, 1.29) | 0.71 |
| Adjusted for total testosterone | 206/282 | 1.18 (0.92, 1.52) | 0.20 |
| Adjusted for SHBG | 218/285 | 1.18 (0.92, 1.51) | 0.20 |
| Adjusted for free testosterone | 206/280 | 1.13 (0.88, 1.46) | 0.33 |

Estimates are from logistic regression conditioned on the matching variables and adjusted for age, BMI, height, alcohol intake, smoking status, marital status, education status, racial/ethnic group, and diabetes status. Estimates were mutually adjusted for biomarkers per study-specific 1 SD entered as a continuous variable.

*Aggressive cancer defined as Gleason grade 8+, or prostate cancer death, or metastases or PSA>100 ng/mL.

†Early-onset defined as diagnosed ≤55 years.

Abbreviations: BMI=body mass index; CI=confidence interval; IGF=insulin-like growth factor; IGFBP=insulin-like growth factor binding protein; OR=odds ratio; SD=standard deviation; SHBG=sex hormone-binding globulin.

**Supplementary Table S9: Risks of overall prostate cancer in relation to biomarkers, stratified by other study-specific median biomarker concentrations in EHNBPCCG studies**

|  |  | IGF-I | | | IGF-II | | | IGFBP-1 | | | IGFBP-2 | | | IGFBP-3 | | |
| --- | --- | --- | --- | --- | --- | --- | --- | --- | --- | --- | --- | --- | --- | --- | --- | --- |
|  |  | Cases/ controls | OR (95 % CI) | *P*_het_ | Cases/ controls | OR (95 % CI) | *P*_het_ | Cases/ controls | OR (95 % CI) | *P*_het_ | Cases/ controls | OR (95 % CI) | *P*_het_ | Cases/ controls | OR (95 % CI) | *P*_het_ |
| IGF-I | <Median |  |  |  | 1974/2626 | 1.08 (1.01, 1.15) |  | 2011/2848 | 0.97 (0.91, 1.02) |  | 1669/2001 | 1.00 (0.94, 1.07) |  | 4270/6059 | 1.07 (1.02, 1.12) |  |
|  | Median+ |  |  |  | 2409/2855 | 1.02 (0.96, 1.08) | 0.2 | 2406/3012 | 0.95 (0.89, 1.02) | 0.72 | 2024/2124 | 0.99 (0.92, 1.08) | 0.89 | 4839/6174 | 1.05 (1.00, 1.10) | 0.61 |
| IGF-II | <Median | 2050/2718 | 1.12 (1.05, 1.20) |  |  |  |  | 1401/1657 | 0.99 (0.92, 1.06) |  | 1697/1962 | 0.99 (0.93, 1.06) |  | 1921/2540 | 1.05 (0.97, 1.15) |  |
|  | Median+ | 2333/2763 | 1.04 (0.98, 1.10) | 0.09 |  |  |  | 1578/1671 | 0.95 (0.87, 1.02) | 0.39 | 1935/1970 | 1.00 (0.93, 1.09) | 0.78 | 2236/2650 | 1.06 (0.99, 1.14) | 0.91 |
| IGFBP-1 | <Median | 2016/2689 | 1.09 (1.03, 1.16) |  | 1269/1397 | 1.04 (0.95, 1.12) |  | 2058/2716 |  |  | 1106/1138 | 1.07 (0.93, 1.23) |  | 1929/2618 | 1.08 (1.01, 1.15) |  |
|  | Median+ | 2401/3171 | 1.11 (1.04, 1.18) | 0.7 | 1710/1931 | 1.11 (1.03, 1.20) | 0.16 | 2433/3222 |  |  | 1528/1626 | 0.92 (0.85, 0.99) | 0.04 | 2340/3097 | 1.09 (1.03, 1.16) | 0.81 |
| IGFBP-2 | <Median | 1768/2078 | 1.10 (1.03, 1.17) |  | 1734/1966 | 1.01 (0.94, 1.08) |  | 1286/1381 | 0.94 (0.82, 1.08) |  |  |  |  | 1717/2032 | 1.04 (0.97, 1.11) |  |
|  | Median+ | 1925/2047 | 1.11 (1.03, 1.19) | 0.88 | 1898/1966 | 1.14 (1.05, 1.22) | 0.02 | 1348/1383 | 0.95 (0.89, 1.02) | 0.91 |  |  |  | 1827/1943 | 1.11 (1.03, 1.19) | 0.15 |
| IGFBP-3 | <Median | 4422/6090 | 1.08 (1.02, 1.14) |  | 2005/2567 | 1.05 (0.96, 1.14) |  | 2077/2854 | 0.96 (0.91, 1.02) |  | 1688/1947 | 0.98 (0.92, 1.05) |  |  |  |  |
|  | Median+ | 4687/6143 | 1.08 (1.03, 1.12) | 1.00 | 2152/2623 | 1.03 (0.95, 1.10) | 0.70 | 2192/2861 | 0.95 (0.89, 1.02) | 0.83 | 1856/2028 | 1.07 (0.98, 1.16) | 0.10 |  |  |  |
| Free testosterone | <Median | 5697/14541 | 1.10 (1.06, 1.14) |  | 1683/1969 | 1.05 (0.98, 1.13) |  | 1402/2075 | 1.00 (0.92, 1.07) |  | 1441/1569 | 0.98 (0.91, 1.06) |  | 3075/4175 | 1.08 (1.03, 1.14) |  |
|  | Median+ | 6154/14560 | 1.07 (1.04, 1.11) | 0.26 | 1953/2048 | 1.07 (1.00, 1.15) | 0.74 | 1577/2152 | 0.94 (0.87, 1.02) | 0.31 | 1686/1558 | 1.03 (0.95, 1.11) | 0.43 | 3393/4236 | 1.06 (1.01, 1.11) | 0.55 |
| Total testosterone | <Median | 6238/15400 | 1.08 (1.05, 1.11) |  | 1828/1975 | 1.01 (0.95, 1.08) |  | 1533/2094 | 1.05 (0.97, 1.14) |  | 1580/1569 | 1.12 (1.02, 1.24) |  | 3286/4226 | 1.05 (1.00, 1.10) |  |
|  | Median+ | 6071/15444 | 1.10 (1.07, 1.14) | 0.41 | 1812/2047 | 1.12 (1.04, 1.21) | 0.03 | 1450/2139 | 0.93 (0.86, 1.00) | 0.02 | 1551/1562 | 0.96 (0.90, 1.04) | 0.01 | 3229/4234 | 1.09 (1.04, 1.15) | 0.24 |
| SHBG | <Median | 6329/14774 | 1.07 (1.04, 1.10) |  | 1924/2019 | 1.05 (0.98, 1.12) |  | 1612/2153 | 0.99 (0.91, 1.09) |  | 1652/1589 | 1.11 (0.99, 1.23) |  | 3430/4292 | 1.06 (1.01, 1.11) |  |
|  | Median+ | 5709/14730 | 1.08 (1.05, 1.12) | 0.62 | 1778/2065 | 1.06 (0.98, 1.14) | 0.85 | 1434/2147 | 0.98 (0.92, 1.05) | 0.83 | 1540/1603 | 1.00 (0.93, 1.07) | 0.09 | 3191/4295 | 1.06 (1.01, 1.12) | 0.90 |

Estimates are from logistic regression conditioned on the matching variables and adjusted for age, BMI, height, alcohol intake, smoking status, marital status, education status, racial/ethnic group, and diabetes status.

Abbreviations: BMI=body mass index; CI=confidence interval; IGF=insulin-like growth factor; IGFBP=insulin-like growth factor binding protein; OR=odds ratio; SD=standard deviation; SHBG=sex hormone binding globulin.

**Supplementary Table S10: Risks of aggressive* prostate cancer in relation to biomarkers, stratified by other study-specific median biomarker concentrations in EHNBPCCG studies**

|  |  | IGF-I | | | IGF-II | | | IGFBP-1 | | | IGFBP-2 | | | IGFBP-3 | | |
| --- | --- | --- | --- | --- | --- | --- | --- | --- | --- | --- | --- | --- | --- | --- | --- | --- |
|  |  | Cases/ controls | OR (95 % CI) | *P*_het_ | Cases/ controls | OR (95 % CI) | *P*_het_ | Cases/ controls | OR (95 % CI) | *P*_het_ | Cases/ controls | OR (95 % CI) | *P*_het_ | Cases/ controls | OR (95 % CI) | *P*_het_ |
| IGF-I | <Median |  |  |  | 310/347 | 0.93 (0.77, 1.13) |  | 401/458 | 0.99 (0.88, 1.11) |  | 275/318 | 0.97 (0.83, 1.15) |  | 733/1047 | 1.06 (0.94, 1.20) |  |
|  | Median+ |  |  |  | 365/405 | 0.85 (0.71, 1.00) | 0.22 | 420/492 | 0.90 (0.76, 1.07) | 0.83 | 316/336 | 0.90 (0.74, 1.08) | 0.54 | 742/981 | 0.96 (0.85, 1.08) | 0.1 |
| IGF-II | <Median | 351/369 | 1.07 (0.91, 1.26) |  |  |  |  | 318/310 | 1.02 (0.89, 1.17) |  | 299/308 | 0.93 (0.78, 1.11) |  | 302/322 | 0.88 (0.69, 1.12) |  |
|  | Median+ | 324/383 | 1.01 (0.86, 1.19) | 0.31 |  |  |  | 292/324 | 0.85 (0.70, 1.03) | 0.73 | 289/315 | 0.89 (0.73, 1.07) | 0.55 | 302/353 | 1.11 (0.91, 1.35) | 0.63 |
| IGFBP-1 | <Median | 361/434 | 0.94 (0.79, 1.11) |  | 263/280 | 0.86 (0.70, 1.04) |  |  |  |  | 235/246 | 0.88 (0.68, 1.15) |  | 323/400 | 0.93 (0.78, 1.09) |  |
|  | Median+ | 460/516 | 1.15 (0.99, 1.33) | 0.03 | 347/354 | 0.92 (0.76, 1.10) | 0.73 |  |  |  | 317/332 | 0.87 (0.74, 1.02) | 0.96 | 435/488 | 1.01 (0.87, 1.18) | 0.52 |
| IGFBP-2 | <Median | 280/314 | 1.07 (0.90, 1.27) |  | 277/293 | 0.94 (0.78, 1.13) |  | 259/260 | 0.84 (0.61, 1.18) |  |  |  |  | 257/295 | 1.01 (0.84, 1.21) |  |
|  | Median+ | 311/340 | 1.14 (0.94, 1.38) | 0.54 | 311/330 | 0.96 (0.78, 1.18) | 0.99 | 293/318 | 0.96 (0.83, 1.10) | 0.93 |  |  |  | 271/296 | 1.01 (0.83, 1.23) | 0.98 |
| IGFBP-3 | <Median | 774/1050 | 1.09 (0.95, 1.24) |  | 328/362 | 0.80 (0.64, 1.00) |  | 414/467 | 0.91 (0.79, 1.04) |  | 275/305 | 0.93 (0.79, 1.11) |  |  |  |  |
|  | Median+ | 701/978 | 1.10 (0.98, 1.23) | 0.66 | 276/313 | 0.99 (0.79, 1.23) | 0.55 | 344/421 | 0.98 (0.82, 1.17) | 0.38 | 253/286 | 1.03 (0.83, 1.28) | 0.49 |  |  |  |
| Free testosterone | <Median | 727/1352 | 1.09 (0.98, 1.21) |  | 268/277 | 0.84 (0.69, 1.03) |  | 277/335 | 0.94 (0.79, 1.13) |  | 221/242 | 1.00 (0.82, 1.22) |  | 557/779 | 0.97 (0.85, 1.10) |  |
|  | Median+ | 622/1255 | 1.10 (0.99, 1.22) | 0.59 | 256/269 | 0.90 (0.73, 1.09) | 0.9 | 254/291 | 0.98 (0.79, 1.21) | 1.00 | 221/200 | 0.88 (0.70, 1.11) | 0.39 | 478/665 | 1.05 (0.91, 1.20) | 0.53 |
| Total testosterone | <Median | 759/1388 | 1.03 (0.94, 1.14) |  | 269/275 | 0.90 (0.74, 1.08) |  | 276/324 | 1.13 (0.92, 1.39) |  | 225/231 | 1.27 (1.00, 1.62) |  | 567/768 | 0.96 (0.85, 1.08) |  |
|  | Median+ | 625/1324 | 1.16 (1.05, 1.30) | 0.08 | 256/273 | 0.85 (0.68, 1.04) | 0.51 | 256/304 | 0.85 (0.70, 1.03) | 0.13 | 218/212 | 0.75 (0.60, 0.93) | <0.01 | 481/690 | 1.08 (0.94, 1.24) | 0.03 |
| SHBG | <Median | 708/1323 | 1.06 (0.95, 1.17) |  | 266/289 | 0.93 (0.78, 1.12) |  | 264/324 | 1.07 (0.85, 1.34) |  | 223/231 | 1.07 (0.82, 1.39) |  | 534/738 | 1.02 (0.90, 1.16) |  |
|  | Median+ | 680/1335 | 1.12 (1.01, 1.25) | 0.68 | 275/274 | 0.80 (0.64, 1.00) | 0.2 | 284/321 | 0.85 (0.72, 1.01) | 0.34 | 236/228 | 0.87 (0.72, 1.06) | 0.27 | 541/751 | 1.00 (0.88, 1.15) | 0.98 |

Estimates are from logistic regression conditioned on the matching variables and adjusted for age, BMI, height, alcohol intake, smoking status, marital status, education status, racial/ethnic group, and diabetes status.

*Aggressive cancer defined as Gleason grade 8+, or prostate cancer death, or metastases or PSA>100 ng/mL.

Abbreviations: BMI=body mass index; CI=confidence interval; IGF=insulin-like growth factor; IGFBP=insulin-like growth factor binding protein; OR=odds ratio; SD=standard deviation; SHBG=sex hormone binding globulin.

**Supplementary Table S11: Association of IGF-I SNPs used in 2-sample Mendelian randomization analyses with prostate cancer**

|  |  |  |  |  |  |  | Association parameters with IGF-I | | | Association parameters with overall prostate cancer | | | Association parameters with aggressive prostate cancer | | | Association parameters with early-onset prostate cancer | | |
| --- | --- | --- | --- | --- | --- | --- | --- | --- | --- | --- | --- | --- | --- | --- | --- | --- | --- | --- |
| SNP | Chr | Position | Consequence | Nearest gene | Effect allele | Other allele | Effect | SE | *P*-value | Effect | SE | *P*-value | Effect | SE | *P*-value | Effect | SE | *P*-value |
| rs36086195 | 1 | 16510894 | intergenic | ARHGEF19-AS1 | T | C | -0.024 | 0.004 | 7.13E-12 | -0.0041 | 0.0077 | 0.5951 | -0.002 | 0.014 | 0.8862 | 0.0167 | 0.0204 | 0.4143 |
| rs114165349 | 1 | 27021913 | intron | ARID1A | C | G | -0.123 | 0.012 | 1.72E-26 | -0.016 | 0.0262 | 0.5406 | -0.0925 | 0.049 | 0.05735 | 0.0756 | 0.0695 | 0.277 |
| rs142840389 | 1 | 29036445 | intron | GMEB1 | A | G | 0.0298 | 0.005 | 1.91E-08 | 0.0011 | 0.012 | 0.9237 | 9.00E-04 | 0.022 | 0.9686 | 0.0423 | 0.0318 | 0.1835 |
| rs12035012 | 1 | 41750648 | downstream | RP11-399E6.1 | A | C | -0.026 | 0.004 | 6.88E-10 | -0.013 | 0.009 | 0.1473 | 0.0139 | 0.016 | 0.3949 | 0.0024 | 0.0237 | 0.9194 |
| rs9787076 | 1 | 44141149 | intron | KDM4A | C | A | 0.0246 | 0.004 | 2.82E-11 | 0.0218 | 0.0079 | 0.005621 | 0.01 | 0.014 | 0.485 | 0.035 | 0.0207 | 0.09104 |
| rs56958645 | 1 | 65223626 | intron | RAVER2 | T | C | 0.038 | 0.006 | 2.59E-11 | 0.0089 | 0.0124 | 0.4708 | -0.0086 | 0.023 | 0.7049 | 0.0178 | 0.0333 | 0.5922 |
| rs469864 | 1 | 91542517 | intergenic | RPL5P6 | C | T | 0.081 | 0.004 | 7.18E-80 | 0.0178 | 0.0094 | 0.05887 | 0.0308 | 0.017 | 0.075 | 0.0372 | 0.0253 | 0.1421 |
| rs6603976 | 1 | 92971622 | downstream | EVI5 | T | C | -0.031 | 0.004 | 9.36E-18 | 1.00E-04 | 0.0077 | 0.9941 | 0.0086 | 0.014 | 0.5453 | -0.035 | 0.0204 | 0.08986 |
| rs599839 | 1 | 109822166 | downstream | CELSR2 | A | G | -0.033 | 0.004 | 2.47E-15 | 0.0043 | 0.0092 | 0.6426 | -0.0176 | 0.017 | 0.2894 | -0.035 | 0.024 | 0.1501 |
| rs1127313 | 1 | 154556425 | 3_prime_UTR | ADAR | A | G | -0.023 | 0.003 | 8.58E-11 | 0.0143 | 0.0078 | 0.06491 | 0.0082 | 0.014 | 0.5667 | -0.004 | 0.0208 | 0.8665 |
| rs12749024 | 1 | 176522365 | intron | PAPPA2 | T | C | 0.0691 | 0.005 | 3.43E-45 | -0.0125 | 0.0111 | 0.2601 | -0.0117 | 0.02 | 0.5617 | 0.0238 | 0.029 | 0.413 |
| rs823094 | 1 | 205689807 | intron | NUCKS1 | G | T | -0.02 | 0.004 | 2.35E-08 | -0.0424 | 0.0075 | 1.74E-08 | -0.0451 | 0.014 | 0.001 | -0.083 | 0.02 | 3.21E-05 |
| rs1204706 | 1 | 208021060 | intron | C1orf132 | T | G | 0.0228 | 0.004 | 1.56E-10 | 0.0034 | 0.0078 | 0.6667 | 0.0156 | 0.014 | 0.2754 | 0.0244 | 0.0206 | 0.2366 |
| rs55843942 | 1 | 214219331 | downstream | PROX1 | G | C | -0.029 | 0.005 | 2.71E-08 | -0.0101 | 0.0132 | 0.4468 | -0.0434 | 0.025 | 0.08317 | -0.046 | 0.0354 | 0.1956 |
| rs17597619 | 1 | 221047861 | upstream | HLX | T | C | -0.049 | 0.004 | 2.93E-34 | -0.0112 | 0.0092 | 0.2245 | -0.0167 | 0.017 | 0.3247 | -0.027 | 0.0248 | 0.2731 |
| rs12048930 | 1 | 243940076 | intron | AKT3 | T | C | -0.038 | 0.004 | 2.06E-17 | -0.0176 | 0.0095 | 0.06422 | 0.0088 | 0.017 | 0.6087 | 0.0414 | 0.0249 | 0.09634 |
| rs57769086 | 2 | 16124792 | intergenic | AC010145.4 | A | C | -0.041 | 0.006 | 1.64E-10 | -0.0055 | 0.0147 | 0.7101 | -0.0574 | 0.027 | 0.03283 | -0.01 | 0.04 | 0.8116 |
| rs72799666 | 2 | 25992902 | intron | ASXL2 | T | C | 0.0532 | 0.009 | 9.36E-09 | 0.0094 | 0.0251 | 0.709 | 0.0731 | 0.046 | 0.1145 | -0.011 | 0.0671 | 0.8742 |
| rs1260326 | 2 | 27730940 | missense | GCKR | C | T | 0.0525 | 0.004 | 1.11E-49 | -0.0237 | 0.0077 | 0.00203 | -0.0191 | 0.014 | 0.1713 | -0.022 | 0.0202 | 0.2688 |
| rs7594734 | 2 | 42705282 | intron | KCNG3 | G | C | 0.032 | 0.005 | 4.85E-10 | 0.0034 | 0.011 | 0.7558 | 0.0024 | 0.02 | 0.9053 | 0.0181 | 0.0293 | 0.5366 |
| rs62135536 | 2 | 44326028 | intergenic | AC019129.1 | T | C | 0.054 | 0.01 | 3.43E-08 | -0.0083 | 0.0233 | 0.7213 | -0.1407 | 0.044 | 0.00126 | -0.026 | 0.062 | 0.6771 |
| rs35624252 | 2 | 70282710 | intron | PCBP1-AS1 | G | T | -0.072 | 0.012 | 2.91E-09 | -0.0116 | 0.0311 | 0.7103 | 0.0066 | 0.057 | 0.9079 | -0.092 | 0.0828 | 0.2656 |
| rs6706968 | 2 | 121310269 | downstream | AC073257.1 | C | A | 0.0302 | 0.004 | 1.20E-17 | 0.002 | 0.0078 | 0.8002 | 0.0087 | 0.014 | 0.54 | 0.0364 | 0.0206 | 0.07817 |
| rs17400325 | 2 | 178565913 | missense | PDE11A | C | T | 0.0515 | 0.009 | 3.61E-09 | 0.0325 | 0.0194 | 0.09479 | 0.0678 | 0.036 | 0.0563 | 0.0164 | 0.0501 | 0.7439 |
| rs1465529 | 2 | 231039037 | intron | SP110 | C | T | -0.021 | 0.004 | 1.56E-08 | 0.0037 | 0.0084 | 0.6645 | 0.0068 | 0.016 | 0.6612 | -0.045 | 0.0226 | 0.04866 |
| rs9872031 | 3 | 12496461 | intergenic | LINC00690 | A | G | 0.02 | 0.004 | 1.08E-08 | -0.0055 | 0.0077 | 0.4728 | -0.0235 | 0.014 | 0.09836 | 0.003 | 0.0205 | 0.8828 |
| rs12496983 | 3 | 23353670 | intron | UBE2E2 | C | A | -0.02 | 0.004 | 1.63E-08 | -0.0065 | 0.0081 | 0.4229 | 0.0071 | 0.015 | 0.6353 | -0.046 | 0.0217 | 0.03383 |
| rs1716975 | 3 | 41960006 | missense | ULK4 | C | T | 0.026 | 0.005 | 3.16E-08 | 0.0016 | 0.0103 | 0.8734 | 0.0116 | 0.019 | 0.5422 | 0.0316 | 0.028 | 0.259 |
| rs10433609 | 3 | 51311574 | intron | DOCK3 | A | T | -0.026 | 0.005 | 1.62E-08 | -0.0112 | 0.0102 | 0.2724 | -0.0213 | 0.019 | 0.2536 | -0.022 | 0.0272 | 0.4178 |
| rs6551280 | 3 | 88201739 | intron | C3orf38 | C | T | 0.0271 | 0.005 | 1.98E-08 | -0.023 | 0.0105 | 0.02931 | -0.0172 | 0.019 | 0.3749 | -0.044 | 0.0279 | 0.113 |
| rs13088318 | 3 | 101242751 | downstream | FAM172BP | G | A | 0.0393 | 0.004 | 1.19E-26 | 0.0226 | 0.008 | 0.004672 | 0.0294 | 0.014 | 0.04158 | 0.0231 | 0.021 | 0.2716 |
| rs895893 | 3 | 135955604 | intergenic | KRT18P35 | T | C | 0.0347 | 0.004 | 2.52E-17 | 0.0147 | 0.009 | 0.1026 | 0.0123 | 0.017 | 0.4598 | 0.023 | 0.0236 | 0.3307 |
| rs55717031 | 3 | 138848505 | intron | MRPS22 | T | G | -0.046 | 0.004 | 1.79E-34 | 0.0073 | 0.0087 | 0.3962 | 5.00E-04 | 0.016 | 0.9751 | 0.0106 | 0.0234 | 0.6524 |
| rs73238159 | 3 | 142078759 | missense | XRN1 | T | C | -0.035 | 0.005 | 1.29E-11 | -0.0206 | 0.0112 | 0.06467 | -0.0038 | 0.021 | 0.8542 | -0.018 | 0.0299 | 0.5588 |
| rs13073970 | 3 | 170630520 | upstream | EIF5A2 | T | G | 0.026 | 0.004 | 1.20E-09 | -0.0168 | 0.0098 | 0.08612 | -0.0061 | 0.018 | 0.7347 | -0.014 | 0.0262 | 0.5925 |
| rs572169 | 3 | 172165727 | synonymous | GHSR | T | C | 0.0568 | 0.004 | 7.31E-53 | 0.0178 | 0.0081 | 0.02758 | 0.0299 | 0.015 | 0.04064 | 0.0199 | 0.0212 | 0.3492 |
| rs4689088 | 4 | 7222253 | intron | SORCS2 | A | G | 0.0305 | 0.004 | 1.24E-17 | -0.002 | 0.0079 | 0.8053 | 0.0172 | 0.014 | 0.2312 | 0.0056 | 0.0207 | 0.7866 |
| rs1055582 | 4 | 39700173 | intron | UBE2K | T | C | -0.031 | 0.003 | 1.98E-19 | -0.0245 | 0.0078 | 0.001595 | -0.0308 | 0.014 | 0.03165 | -0.022 | 0.0208 | 0.2873 |
| rs7662792 | 4 | 45121873 | intergenic | NMU | T | A | -0.039 | 0.004 | 1.36E-26 | 0.0086 | 0.0083 | 0.2989 | 0.0154 | 0.015 | 0.3106 | 0.0277 | 0.0221 | 0.2105 |
| rs9884390 | 4 | 69373407 | downstream | UGT2B29P | C | T | -0.051 | 0.004 | 4.29E-35 | -0.0032 | 0.0102 | 0.7543 | 0.0199 | 0.019 | 0.2824 | 0.0014 | 0.0273 | 0.9585 |
| rs139916529 | 4 | 69564066 | intergenic | AC112518.3 | T | G | 0.04 | 0.007 | 4.62E-09 | -0.0262 | 0.0165 | 0.113 | -0.0379 | 0.031 | 0.2132 | -0.031 | 0.0442 | 0.4896 |
| rs8582 | 4 | 90035967 | 3_prime_UTR | TIGD2 | A | T | 0.0298 | 0.005 | 4.65E-11 | 0.0349 | 0.0098 | 0.000355 | 0.0404 | 0.018 | 0.02501 | 0.0329 | 0.0261 | 0.2079 |
| rs1229984 | 4 | 100239319 | missense | ADH1B | C | T | -0.15 | 0.012 | 1.04E-37 | 0.0452 | 0.0207 | 0.02885 | 0.067 | 0.04 | 0.09676 | 0.0573 | 0.0574 | 0.3185 |
| rs2358184 | 4 | 148985906 | intron | ARHGAP10 | G | A | 0.0323 | 0.004 | 1.33E-15 | 0.014 | 0.0091 | 0.1217 | -0.0035 | 0.017 | 0.8317 | -0.003 | 0.0242 | 0.8983 |
| rs55681913 | 5 | 42687629 | intron | GHR | C | T | 0.0599 | 0.006 | 4.82E-26 | 0.001 | 0.013 | 0.9365 | 0.0225 | 0.024 | 0.349 | -0.042 | 0.0363 | 0.2436 |
| rs315262 | 5 | 42888022 | upstream | SELENOP | T | C | 0.0277 | 0.004 | 1.27E-12 | 0.0181 | 0.0089 | 0.0411 | 0.0209 | 0.016 | 0.195 | -0.015 | 0.0237 | 0.5364 |
| rs7719168 | 5 | 53292390 | intron | ARL15 | C | A | 0.0324 | 0.006 | 3.96E-09 | -0.0011 | 0.0119 | 0.9291 | 0.02 | 0.022 | 0.3565 | 0.0116 | 0.0314 | 0.7124 |
| rs298068 | 5 | 58978163 | intron | PDE4D | A | T | 0.0343 | 0.004 | 2.03E-20 | -0.0088 | 0.008 | 0.2677 | -0.0225 | 0.015 | 0.122 | -0.036 | 0.0209 | 0.08605 |
| rs7721818 | 5 | 88394669 | intron | MEF2C-AS1 | A | G | -0.032 | 0.004 | 2.12E-19 | 0.0056 | 0.0079 | 0.4824 | 0.0067 | 0.015 | 0.6491 | 0.0076 | 0.0212 | 0.721 |
| rs11242236 | 5 | 134586980 | intron | C5orf66 | A | G | -0.031 | 0.003 | 1.50E-18 | 0.0024 | 0.0076 | 0.7483 | 0.0216 | 0.014 | 0.1234 | 0.0075 | 0.0203 | 0.713 |
| rs11334 | 5 | 137772296 | 3_prime_UTR | KDM3B | C | G | 0.0377 | 0.004 | 2.81E-18 | 0.025 | 0.0092 | 0.006478 | 0.03 | 0.017 | 0.07841 | 0.0543 | 0.0247 | 0.02806 |
| rs2974433 | 5 | 168268387 | intron | SLIT3 | C | T | -0.049 | 0.004 | 2.75E-33 | 0.0125 | 0.0092 | 0.1715 | -0.0258 | 0.017 | 0.1244 | 0.0041 | 0.0244 | 0.8671 |
| rs9379822 | 6 | 26070672 | intergenic | ZFP57 | G | T | -0.04 | 0.004 | 8.04E-28 | -0.0113 | 0.0078 | 0.1472 | -0.0141 | 0.014 | 0.3247 | -0.046 | 0.0207 | 0.02682 |
| rs9470825 | 6 | 38162310 | intron | BTBD9 | G | A | 0.0279 | 0.004 | 1.71E-12 | -0.0038 | 0.0085 | 0.6584 | 0.0042 | 0.016 | 0.7865 | 0.0144 | 0.0229 | 0.5297 |
| rs3734187 | 6 | 87969737 | synonymous | ZNF292 | T | C | -0.032 | 0.003 | 1.44E-20 | 0.0231 | 0.0075 | 0.002117 | 0.0075 | 0.014 | 0.5858 | -0.004 | 0.0198 | 0.8489 |
| rs395962 | 6 | 105397418 | intron | LIN28B | G | T | 0.0403 | 0.004 | 1.43E-27 | 0.0039 | 0.008 | 0.6283 | -0.0206 | 0.015 | 0.1543 | 0.0215 | 0.021 | 0.3042 |
| rs4946936 | 6 | 109003321 | 3_prime_UTR | FOXO3 | C | T | 0.0515 | 0.004 | 2.56E-40 | 0.0142 | 0.0084 | 0.09228 | 0.0334 | 0.016 | 0.03181 | 0.0183 | 0.0224 | 0.4139 |
| rs9398805 | 6 | 126726295 | intergenic | RP11-394G3.2 | T | C | 0.0744 | 0.003 | 1.31E-101 | 0.0085 | 0.0075 | 0.2563 | 0.0018 | 0.014 | 0.8981 | -0.042 | 0.0201 | 0.037 |
| rs790513 | 6 | 154420368 | intron | OPRM1 | C | A | 0.0353 | 0.004 | 8.47E-19 | -0.0072 | 0.0088 | 0.4111 | -1.00E-04 | 0.016 | 0.9944 | 0.0072 | 0.0233 | 0.7587 |
| rs668871 | 6 | 160769811 | synonymous | SLC22A3 | T | C | -0.036 | 0.003 | 1.59E-24 | -0.0823 | 0.0074 | 1.50E-28 | -0.0717 | 0.014 | 1.11E-07 | -0.131 | 0.0196 | 1.85E-11 |
| rs7381453 | 6 | 166313164 | upstream | SDIM1 | G | A | 0.0456 | 0.004 | 2.72E-37 | 0.0206 | 0.008 | 0.01039 | 0.0219 | 0.015 | 0.137 | 0.0267 | 0.0216 | 0.2154 |
| rs7802508 | 7 | 1191689 | downstream | ZFAND2A | A | G | 0.0266 | 0.004 | 4.19E-14 | -0.0023 | 0.0078 | 0.7684 | -0.0011 | 0.014 | 0.9355 | 0.001 | 0.0203 | 0.9593 |
| rs75165920 | 7 | 6743332 | intron | ZNF12 | A | T | -0.055 | 0.005 | 5.69E-28 | 0.0021 | 0.011 | 0.8483 | -0.0103 | 0.02 | 0.6078 | 0.0079 | 0.0291 | 0.7857 |
| rs112293610 | 7 | 14226261 | intron | DGKB | C | A | -0.027 | 0.004 | 1.95E-12 | 0.0164 | 0.0086 | 0.05578 | 0.0162 | 0.016 | 0.3003 | 0.0417 | 0.0229 | 0.06876 |
| rs4988501 | 7 | 31011485 | intron | GHRHR | G | A | -0.032 | 0.004 | 2.02E-15 | 0.0122 | 0.0098 | 0.2121 | -0.0059 | 0.018 | 0.744 | -0.019 | 0.0268 | 0.4853 |
| rs62460538 | 7 | 44940256 | intergenic | RP4-673M15.1 | C | T | -0.044 | 0.007 | 5.36E-11 | -0.0227 | 0.016 | 0.1569 | 0.0118 | 0.03 | 0.6888 | -0.1 | 0.0424 | 0.01891 |
| rs10243669 | 7 | 45915907 | intergenic | AC096582.1 | C | T | 0.0398 | 0.005 | 5.14E-16 | -0.0023 | 0.0103 | 0.8218 | -0.0085 | 0.019 | 0.6562 | 0.0098 | 0.028 | 0.7254 |
| rs700750 | 7 | 46753491 | intron | AC011294.3 | A | C | 0.1145 | 0.004 | 1.00E-200 | -0.0085 | 0.0079 | 0.2811 | -0.0282 | 0.014 | 0.05018 | 0.003 | 0.0207 | 0.8856 |
| rs4727799 | 7 | 114110568 | intron | FOXP2 | T | C | -0.024 | 0.004 | 6.77E-11 | -0.0052 | 0.008 | 0.516 | -0.0041 | 0.015 | 0.7802 | -0.006 | 0.0211 | 0.7693 |
| rs125124 | 7 | 130584684 | intron | AC058791.1 | G | C | 0.0612 | 0.004 | 7.81E-59 | -0.0079 | 0.0087 | 0.3631 | 0.0046 | 0.016 | 0.7703 | 0.02 | 0.0231 | 0.3859 |
| rs273963 | 7 | 137594938 | intron | CREB3L2 | C | G | -0.022 | 0.004 | 1.26E-09 | 0.0025 | 0.0081 | 0.7604 | 0.0125 | 0.015 | 0.3993 | 0.0143 | 0.0217 | 0.5092 |
| rs41341748 | 8 | 16012594 | stop_lost | MSR1 | A | G | -0.106 | 0.016 | 8.59E-11 | -0.0128 | 0.0386 | 0.7407 | -0.0053 | 0.072 | 0.9411 | -0.135 | 0.1036 | 0.1942 |
| rs4738684 | 8 | 59393273 | intergenic | CYP7A1 | G | A | -0.033 | 0.004 | 1.61E-19 | 0.0019 | 0.008 | 0.8077 | -0.0159 | 0.015 | 0.2765 | -0.021 | 0.0212 | 0.334 |
| rs13282738 | 8 | 77122534 | intergenic | RNU2-54P | G | A | 0.0244 | 0.004 | 5.04E-12 | -0.0086 | 0.0076 | 0.2604 | 0.0145 | 0.014 | 0.3004 | -0.009 | 0.0202 | 0.6534 |
| rs2293889 | 8 | 116599199 | intron | TRPS1 | G | T | -0.029 | 0.004 | 3.55E-16 | 0.0122 | 0.0076 | 0.1102 | 0.0027 | 0.014 | 0.8461 | 0.0278 | 0.02 | 0.1644 |
| rs295263 | 9 | 4840063 | intron | RCL1 | T | C | 0.0409 | 0.005 | 2.26E-14 | 0.0248 | 0.0121 | 0.04022 | 0.0217 | 0.022 | 0.3326 | 0.0328 | 0.0325 | 0.3141 |
| rs41303235 | 9 | 4985388 | 5_prime_UTR | JAK2 | T | C | 0.06 | 0.008 | 1.46E-12 | -0.0226 | 0.0202 | 0.2639 | 0.0435 | 0.037 | 0.2349 | 0.0284 | 0.0521 | 0.5863 |
| rs62560861 | 9 | 34078582 | intergenic | RP11-537H15.3 | A | C | 0.0261 | 0.004 | 2.67E-09 | -0.0302 | 0.0097 | 0.001789 | -0.054 | 0.018 | 0.00241 | -0.043 | 0.0256 | 0.09043 |
| rs10869022 | 9 | 74057313 | intron | TRPM3 | C | T | 0.0332 | 0.004 | 2.79E-14 | 0.0178 | 0.0097 | 0.06657 | 0.0372 | 0.018 | 0.03739 | 0.0123 | 0.026 | 0.6379 |
| rs10156602 | 9 | 96345328 | intron | PHF2 | G | A | -0.027 | 0.004 | 9.52E-14 | -0.01 | 0.0079 | 0.2035 | 0.0103 | 0.015 | 0.476 | -0.023 | 0.021 | 0.2838 |
| rs925813 | 9 | 97525627 | 3_prime_UTR | C9orf3 | C | T | -0.052 | 0.007 | 3.11E-13 | -0.0279 | 0.0155 | 0.0727 | 0.008 | 0.028 | 0.7728 | -0.078 | 0.042 | 0.0648 |
| rs78509281 | 9 | 109566543 | intergenic | RNA5SP292 | T | C | 0.054 | 0.008 | 1.54E-11 | -0.035 | 0.0181 | 0.05241 | -0.0537 | 0.033 | 0.1081 | -0.069 | 0.048 | 0.1499 |
| rs12553882 | 9 | 128195044 | downstream | MAPKAP1 | A | G | -0.024 | 0.004 | 3.38E-11 | 0.0159 | 0.0081 | 0.05095 | 0.0265 | 0.015 | 0.0751 | 0.0281 | 0.0217 | 0.1952 |
| rs1832007 | 10 | 5254847 | intron | AKR1C4 | G | A | 0.0366 | 0.005 | 2.45E-14 | -0.0069 | 0.0103 | 0.5037 | -0.0263 | 0.019 | 0.1627 | -0.004 | 0.027 | 0.8747 |
| rs12780653 | 10 | 21693629 | intergenic | RNMTL1P1 | A | G | -0.026 | 0.005 | 3.15E-08 | 0.009 | 0.0105 | 0.3922 | 0.021 | 0.019 | 0.2721 | 0.0091 | 0.0277 | 0.7432 |
| rs10821719 | 10 | 62076964 | intron | ANK3 | C | T | 0.0203 | 0.004 | 1.10E-08 | -0.0047 | 0.0077 | 0.5395 | 0.0141 | 0.014 | 0.32 | -0.016 | 0.0204 | 0.4243 |
| rs4418728 | 10 | 94839724 | downstream | CYP26A1 | T | G | -0.027 | 0.003 | 1.35E-14 | -0.0162 | 0.0075 | 0.03141 | -0.0054 | 0.014 | 0.6951 | -0.03 | 0.02 | 0.1363 |
| rs9630085 | 10 | 95333063 | intron | FFAR4 | G | A | 0.0241 | 0.004 | 2.81E-08 | 0.0076 | 0.0096 | 0.4304 | -0.0049 | 0.018 | 0.785 | 0.0414 | 0.0255 | 0.1049 |
| rs35391272 | 10 | 125058393 | intergenic | RP11-338O1.3 | T | G | 0.0274 | 0.005 | 1.90E-08 | 0.0179 | 0.0106 | 0.09079 | 0.0138 | 0.019 | 0.4757 | 0.0189 | 0.0283 | 0.5036 |
| rs3842763 | 11 | 2179204 | intron | INS-IGF2 | T | G | -0.078 | 0.004 | 1.01E-78 | -0.0067 | 0.0089 | 0.4524 | -0.0261 | 0.016 | 0.1036 | -0.007 | 0.0231 | 0.7583 |
| rs1037169 | 11 | 13361005 | intron | ARNTL | C | T | 0.0224 | 0.004 | 2.54E-09 | 0.0134 | 0.0081 | 0.09782 | 0.0242 | 0.015 | 0.1018 | -0.007 | 0.0214 | 0.7616 |
| rs2271997 | 11 | 18303404 | intron | HPS5 | T | C | -0.024 | 0.004 | 1.81E-11 | 0.0152 | 0.0077 | 0.04793 | 0.0201 | 0.014 | 0.1552 | 0.0261 | 0.0204 | 0.2015 |
| rs1039481 | 11 | 48182237 | intron | PTPRJ | A | G | -0.041 | 0.004 | 4.24E-25 | 0.0075 | 0.0088 | 0.3939 | -0.0159 | 0.016 | 0.33 | 0.0095 | 0.0234 | 0.6859 |
| rs174546 | 11 | 61569830 | 3_prime_UTR | FADS1 | T | C | -0.033 | 0.004 | 6.09E-20 | -0.005 | 0.008 | 0.531 | 0.0199 | 0.015 | 0.172 | -0.042 | 0.0212 | 0.04754 |
| rs117104648 | 11 | 65543736 | 3_prime_UTR | AP5B1 | C | T | 0.0434 | 0.007 | 2.23E-09 | -0.0235 | 0.0162 | 0.1469 | -0.0368 | 0.03 | 0.2151 | -0.025 | 0.0452 | 0.5879 |
| rs291217 | 11 | 86033229 | intron | HIKESHI | A | G | 0.0214 | 0.003 | 7.62E-10 | 0.0183 | 0.0075 | 0.01476 | 0.0162 | 0.014 | 0.2346 | 0.0455 | 0.0196 | 0.0205 |
| rs71477696 | 11 | 94133404 | intron | GPR83 | G | T | -0.078 | 0.01 | 7.70E-16 | -0.0178 | 0.0248 | 0.4742 | 0.0115 | 0.045 | 0.7992 | 0.003 | 0.0652 | 0.9636 |
| rs2856321 | 12 | 11855773 | intron | ETV6 | A | G | -0.021 | 0.004 | 6.11E-09 | -0.0114 | 0.0079 | 0.1484 | -0.022 | 0.014 | 0.1244 | -0.009 | 0.0209 | 0.6553 |
| rs9738365 | 12 | 31997635 | intergenic | RP11-428G5.4 | A | C | 0.0668 | 0.004 | 9.33E-65 | -0.0012 | 0.0085 | 0.8841 | 0.0011 | 0.016 | 0.9441 | -0.006 | 0.0225 | 0.7882 |
| rs9669278 | 12 | 66374587 | intergenic | HMGA2 | C | T | 0.0209 | 0.003 | 2.35E-09 | 1.00E-04 | 0.0076 | 0.9899 | 0.0061 | 0.014 | 0.6628 | 0.0017 | 0.0202 | 0.9344 |
| rs7953987 | 12 | 98167068 | intergenic | RP11-1016B18.1 | A | G | 0.0276 | 0.004 | 1.25E-14 | -0.0015 | 0.0078 | 0.8491 | -0.0184 | 0.014 | 0.1969 | -0.034 | 0.0207 | 0.09963 |
| rs5742653 | 12 | 102835859 | intron | IGF1 | T | C | -0.072 | 0.004 | 6.95E-75 | -0.0269 | 0.0084 | 0.001282 | -0.0267 | 0.015 | 0.08093 | -0.054 | 0.022 | 0.01444 |
| rs1800574 | 12 | 121416864 | missense | HNF1A | T | C | 0.1682 | 0.01 | 5.48E-60 | -0.0225 | 0.0233 | 0.3324 | 0.0024 | 0.042 | 0.9539 | -0.069 | 0.0639 | 0.2795 |
| rs4617691 | 13 | 21356650 | 3_prime_UTR | XPO4 | A | T | -0.021 | 0.004 | 1.75E-08 | -0.004 | 0.008 | 0.6171 | 0.0011 | 0.015 | 0.9429 | 0.0119 | 0.0213 | 0.5755 |
| rs10507482 | 13 | 40755641 | intron | LINC00598 | G | A | 0.0484 | 0.004 | 5.70E-27 | -0.0035 | 0.0098 | 0.7248 | -0.005 | 0.018 | 0.7809 | 0.0031 | 0.0259 | 0.9057 |
| rs6561323 | 13 | 47185601 | intron | LRCH1 | G | A | 0.0254 | 0.004 | 3.09E-10 | 0.0068 | 0.0091 | 0.4548 | 0.0169 | 0.016 | 0.304 | 0.0456 | 0.024 | 0.05777 |
| rs61957204 | 13 | 74084684 | intron | LINC00393 | A | G | 0.0488 | 0.006 | 2.68E-14 | -0.1071 | 0.0163 | 5.45E-11 | -0.0647 | 0.031 | 0.03368 | -0.189 | 0.0454 | 3.27E-05 |
| rs71432868 | 13 | 106559402 | intergenic | SNORA25 | C | T | 0.0372 | 0.007 | 4.99E-08 | 0.0303 | 0.0191 | 0.1138 | 0.0916 | 0.037 | 0.01355 | 0.1055 | 0.053 | 0.04675 |
| rs6602909 | 13 | 114551993 | intron | GAS6 | C | T | 0.0216 | 0.004 | 5.69E-09 | 0.0072 | 0.0089 | 0.4176 | 0.0172 | 0.016 | 0.2845 | 0.003 | 0.0238 | 0.8985 |
| rs11620783 | 14 | 24871530 | intron | NYNRIN | T | C | -0.027 | 0.004 | 8.60E-15 | 0.0044 | 0.0082 | 0.5904 | 0.0192 | 0.015 | 0.2015 | 0.0302 | 0.0219 | 0.1682 |
| rs6573307 | 14 | 60798009 | intergenic | CTD-2568P8.1 | G | T | 0.025 | 0.004 | 3.77E-12 | -0.0094 | 0.0078 | 0.2255 | 0.0201 | 0.014 | 0.1564 | -0.053 | 0.0206 | 0.009693 |
| rs10145740 | 14 | 74234294 | intron | ELMSAN1 | T | C | -0.028 | 0.004 | 2.84E-12 | -0.0023 | 0.0092 | 0.7991 | -0.0189 | 0.017 | 0.26 | 0.0645 | 0.0239 | 0.007031 |
| rs28929474 | 14 | 94844947 | missense | SERPINA1 | T | C | -0.122 | 0.012 | 3.15E-23 | -0.146 | 0.0285 | 3.04E-07 | 0.0014 | 0.049 | 0.978 | -0.183 | 0.0752 | 0.01467 |
| rs876375 | 14 | 101204319 | 3_prime_UTR | DLK1 | G | A | -0.028 | 0.003 | 1.23E-15 | 0.0041 | 0.0076 | 0.5902 | 0.0072 | 0.014 | 0.6045 | 0.0177 | 0.0201 | 0.3789 |
| rs190543502 | 15 | 43757184 | intron | TP53BP1 | C | T | -0.141 | 0.012 | 3.52E-34 | -0.0245 | 0.0282 | 0.3856 | -0.0542 | 0.054 | 0.3116 | -0.066 | 0.0766 | 0.3867 |
| rs12592402 | 15 | 62349020 | intron | VPS13C | G | A | -0.036 | 0.003 | 2.51E-24 | 0.0057 | 0.0078 | 0.4714 | 0.0178 | 0.014 | 0.2104 | 0.0184 | 0.0209 | 0.3789 |
| rs62011346 | 15 | 63850067 | intron | USP3 | G | A | -0.028 | 0.005 | 5.43E-09 | 0.0032 | 0.0101 | 0.7504 | 0.0093 | 0.019 | 0.6164 | -0.02 | 0.0274 | 0.4741 |
| rs5742915 | 15 | 74336633 | missense | PML | C | T | 0.0307 | 0.003 | 1.06E-18 | -0.002 | 0.0077 | 0.7913 | 0.0082 | 0.014 | 0.5551 | -0.03 | 0.0201 | 0.1394 |
| rs76819935 | 16 | 1110581 | upstream | RP11-161M6.5 | C | T | -0.152 | 0.008 | 1.39E-86 | -0.0082 | 0.0242 | 0.7337 | -0.0851 | 0.044 | 0.05509 | 0.0711 | 0.0621 | 0.252 |
| rs8048693 | 16 | 1811565 | intron | MAPK8IP3 | G | A | -0.045 | 0.004 | 8.18E-36 | -0.0258 | 0.0082 | 0.001698 | -0.0081 | 0.015 | 0.5842 | -0.045 | 0.0217 | 0.0368 |
| rs1369924 | 16 | 5924291 | intron | RP11-420N3.3 | C | A | -0.037 | 0.004 | 1.25E-16 | 0.017 | 0.01 | 0.08973 | 0.0198 | 0.019 | 0.2833 | 0.0251 | 0.0269 | 0.3523 |
| rs12935465 | 16 | 17476853 | intron | XYLT1 | C | T | -0.02 | 0.003 | 1.08E-08 | -0.0059 | 0.0078 | 0.4453 | 0.0103 | 0.014 | 0.4679 | 0.0341 | 0.0204 | 0.09548 |
| rs73530203 | 16 | 31099859 | intron | PRSS53 | A | G | -0.035 | 0.004 | 1.12E-22 | -0.0034 | 0.0077 | 0.6606 | -0.0052 | 0.014 | 0.7133 | 0.0158 | 0.0205 | 0.4405 |
| rs8062941 | 16 | 69578482 | intergenic | NFAT5 | G | A | 0.0342 | 0.005 | 1.24E-12 | -0.0149 | 0.0109 | 0.1704 | -0.0229 | 0.02 | 0.253 | 0.0022 | 0.0288 | 0.9379 |
| rs8059803 | 16 | 81603001 | intron | CMIP | A | G | 0.0395 | 0.004 | 1.79E-25 | 0.0012 | 0.0089 | 0.8915 | 0.0177 | 0.016 | 0.2782 | 0.0546 | 0.0237 | 0.02159 |
| rs858519 | 17 | 7531965 | intron | SHBG | C | T | 0.0236 | 0.003 | 1.70E-11 | -0.0035 | 0.0077 | 0.6533 | -0.0101 | 0.014 | 0.4752 | 0.0175 | 0.0203 | 0.3874 |
| rs2779211 | 17 | 15875935 | intron | ADORA2B | G | A | -0.021 | 0.003 | 8.82E-10 | 0.0096 | 0.0077 | 0.2132 | -0.0251 | 0.014 | 0.07616 | 0.0131 | 0.0206 | 0.5228 |
| rs8075153 | 17 | 17622666 | intron | RAI1 | T | C | -0.023 | 0.003 | 5.92E-11 | -0.0277 | 0.0076 | 0.000246 | -0.0306 | 0.014 | 0.02544 | -0.054 | 0.0198 | 0.006001 |
| rs6503533 | 17 | 38184580 | intron | MED24 | T | C | 0.0244 | 0.004 | 1.07E-11 | 0.0149 | 0.0077 | 0.05246 | 0.0212 | 0.014 | 0.1319 | 0.016 | 0.0204 | 0.4336 |
| rs1991556 | 17 | 44083402 | intron | MAPT | A | G | 0.0343 | 0.004 | 1.84E-16 | -0.0208 | 0.0092 | 0.02462 | -0.027 | 0.017 | 0.1149 | -0.072 | 0.024 | 0.002936 |
| rs8070132 | 17 | 57790206 | intron | VMP1 | C | T | -0.029 | 0.005 | 2.50E-09 | -0.0057 | 0.0106 | 0.5927 | -0.0361 | 0.02 | 0.06359 | -0.037 | 0.028 | 0.1883 |
| rs2005172 | 17 | 61996255 | upstream | GH1 | C | A | 0.0363 | 0.004 | 3.27E-23 | -0.0183 | 0.0081 | 0.02283 | -0.0155 | 0.015 | 0.2858 | -0.063 | 0.0211 | 0.002944 |
| rs878371 | 17 | 73816844 | intron | UNK | A | G | 0.0264 | 0.004 | 2.15E-13 | 0.0018 | 0.0084 | 0.8258 | -0.0035 | 0.015 | 0.8199 | 0.029 | 0.0223 | 0.1927 |
| rs4075482 | 17 | 79074548 | intron | BAIAP2 | A | C | -0.023 | 0.004 | 3.01E-10 | 0.0116 | 0.0085 | 0.1749 | -2.00E-04 | 0.015 | 0.9908 | 0.0031 | 0.022 | 0.8885 |
| rs12454712 | 18 | 60845884 | intron | BCL2 | C | T | -0.023 | 0.004 | 7.84E-11 | 0.0032 | 0.0078 | 0.6816 | 0.0033 | 0.014 | 0.8196 | -0.012 | 0.0206 | 0.5636 |
| rs59360013 | 18 | 74976835 | intron | GALR1 | T | C | -0.074 | 0.009 | 5.74E-18 | 0.0054 | 0.0195 | 0.7813 | -0.0402 | 0.036 | 0.2603 | 0.0161 | 0.0537 | 0.765 |
| rs8105174 | 19 | 10347032 | intergenic | DNMT1 | T | C | -0.052 | 0.004 | 1.93E-31 | 0.0059 | 0.0104 | 0.5711 | 0.0286 | 0.019 | 0.1239 | -0.016 | 0.0267 | 0.5531 |
| rs34536443 | 19 | 10463118 | missense | TYK2 | C | G | -0.052 | 0.009 | 1.26E-09 | 0.0323 | 0.0211 | 0.126 | 0.0779 | 0.038 | 0.0413 | 0.0804 | 0.0541 | 0.1374 |
| rs33428 | 19 | 30937843 | intron | ZNF536 | G | A | -0.021 | 0.004 | 1.21E-08 | -0.0085 | 0.0086 | 0.3211 | -0.0138 | 0.016 | 0.3825 | -0.06 | 0.023 | 0.009395 |
| rs6510177 | 19 | 31211647 | intergenic | ZNF536 | C | T | -0.033 | 0.004 | 6.00E-14 | 0.0179 | 0.0118 | 0.1295 | 1.00E-04 | 0.021 | 0.9971 | 0.006 | 0.0317 | 0.8508 |
| rs12975366 | 19 | 54759361 | missense | LILRB5 | C | T | -0.022 | 0.004 | 6.74E-10 | -0.0117 | 0.0083 | 0.1587 | -0.0133 | 0.015 | 0.3846 | 0.012 | 0.0222 | 0.5894 |
| rs73125628 | 20 | 20066701 | intron | CFAP61 | T | C | -0.045 | 0.004 | 1.56E-30 | 0.0116 | 0.0085 | 0.1738 | 0.0167 | 0.016 | 0.2815 | 0.0199 | 0.0228 | 0.3827 |
| rs6035811 | 20 | 21203946 | intron | KIZ | C | G | -0.061 | 0.004 | 1.68E-61 | -0.0119 | 0.0082 | 0.1464 | -0.0124 | 0.015 | 0.4061 | -0.007 | 0.0215 | 0.7471 |
| rs2268879 | 20 | 25177805 | 5_prime_UTR | ENTPD6 | C | T | -0.02 | 0.004 | 2.48E-08 | -0.0208 | 0.0079 | 0.008221 | -0.0113 | 0.014 | 0.429 | -0.014 | 0.0209 | 0.49 |
| rs1883711 | 20 | 39179822 | intergenic | LINC01728 | C | G | -0.082 | 0.01 | 9.33E-16 | 0.0466 | 0.0231 | 0.04327 | 0.0941 | 0.042 | 0.02459 | -0.034 | 0.0666 | 0.6083 |
| rs17265513 | 20 | 39832628 | missense | ZHX3 | C | T | -0.035 | 0.004 | 1.10E-15 | -0.0139 | 0.0097 | 0.1548 | -0.0291 | 0.018 | 0.1046 | -0.012 | 0.0265 | 0.6414 |
| rs78676352 | 20 | 49191939 | intron | PTPN1 | T | A | -0.048 | 0.006 | 9.00E-14 | 0.0174 | 0.0151 | 0.2487 | 0.0155 | 0.028 | 0.5764 | -0.051 | 0.041 | 0.2175 |
| rs932792 | 20 | 54851746 | intergenic | RP13-379L11.3 | A | C | -0.028 | 0.004 | 2.92E-14 | -0.0069 | 0.0079 | 0.3839 | -0.0156 | 0.014 | 0.2785 | 0.0147 | 0.021 | 0.4822 |
| rs185799410 | 20 | 57466093 | intron | GNAS | T | G | -0.068 | 0.011 | 7.84E-10 | -0.016 | 0.0274 | 0.5596 | -0.0066 | 0.05 | 0.8954 | -0.061 | 0.0736 | 0.4094 |
| rs17274750 | 21 | 16353809 | intron | NRIP1 | C | A | -0.038 | 0.006 | 5.68E-11 | 0.002 | 0.0134 | 0.8836 | 0.011 | 0.025 | 0.6551 | 0.05 | 0.0358 | 0.163 |
| rs7280982 | 21 | 37468223 | intron | AP000688.14 | A | G | -0.053 | 0.004 | 3.39E-36 | -0.0299 | 0.009 | 0.000932 | -0.0309 | 0.017 | 0.06068 | -0.045 | 0.0238 | 0.05907 |
| rs117217365 | 22 | 32220804 | intron | DEPDC5 | T | A | 0.0585 | 0.01 | 4.55E-09 | -0.0028 | 0.0202 | 0.8896 | 0.0356 | 0.037 | 0.3287 | -0.108 | 0.0572 | 0.05889 |
| rs6519133 | 22 | 39096602 | 5_prime_UTR | JOSD1 | C | T | -0.026 | 0.004 | 2.69E-13 | 5.00E-04 | 0.0079 | 0.9461 | 0.0183 | 0.015 | 0.2094 | 0.0414 | 0.0211 | 0.0494 |
| rs4823173 | 22 | 44328730 | intron | PNPLA3 | A | G | -0.034 | 0.005 | 4.75E-13 | -0.0335 | 0.0102 | 0.001003 | -0.0205 | 0.019 | 0.2701 | 0.0032 | 0.0272 | 0.905 |

*Aggressive cancer defined as Gleason grade 8+, or prostate cancer death, or metastases or PSA>100. Early-onset defined as diagnosed ≤ 55 years.

Abbreviations: Chr=chromosome; PSA=prostate-specific antigen; SE=standard error; SNP=single nucleotide polymorphism UTR=untranslated region.

**Supplementary Table S12: Outlier SNPs identified by MR-PRESSO***

|  | Prostate cancer | | |
| --- | --- | --- | --- |
|  | Overall | Aggressive | Early-onset |
| Outlier SNPs | rs28929474 | rs668871 | rs61957204 |
|  | rs61957204 | | rs668871 |
|  | rs668871 |  | rs823094 |
|  | rs823094 |  |  |
|  | rs1260326 |  |  |

*Aggressive cancer defined as Gleason grade 8+, or prostate cancer death, or metastases or PSA>100. Early-onset defined as diagnosed ≤55 years.

Abbreviations: MR=Mendelian randomization; PRESSO=pleiotropy residual sum and outlier; PSA= prostate-specific antigen; SNP= single nucleotide polymorphism.

|  |  | Overall prostate cancer  (85,554 cases, 91,972 controls) | | Aggressive prostate cancer*  (15,167 cases, 58,308 controls) | | Early-onset prostate cancer†  (6,988 cases, 44,256 controls) | |
| --- | --- | --- | --- | --- | --- | --- | --- |
|  |  | OR per 1 SD increment (95% CI) | *P*-value | OR per 1 SD increment (95% CI) | *P*-value | OR per 1 SD increment (95% CI) | *P*-value |
| IGF-I (SD=5.4 nmol/L) |  |  |  |  |  |  |  |
| Inverse-variance weighted |  | 1.07 (1.00, 1.14) | 0.06 | 1.09 (0.96, 1.24) | 0.20 | 1.09 (0.96, 1.24) | 0.20 |
| Weighted median |  | 1.01 (0.95, 1.08) | 0.73 | 1.06 (0.89, 1.27) | 0.48 | 1.07 (0.89, 1.27) | 0.48 |
| MR-Egger |  | 1.02 (0.87, 1.19) | 0.81 | 1.01 (0.76, 1.36) | 0.90 | 1.02 (0.76, 1.36) | 0.90 |
| MR-Egger intercept |  |  | 0.50 |  | 0.38 |  | 0.62 |
| MR-RAPS |  | 1.04 (0.97, 1.11) | 0.23 | 1.11 (1.00, 1.22) | 0.04 | 1.09 (0.95, 1.26) | 0.20 |
| MR-PRESSO |  | 1.06 (1.00, 1.12) | 0.05 | 1.08 (0.99, 1.18) | 0.08 | 1.10 (0.97, 1.25) | 0.13 |
| Contamination mixture |  | 0.98 (0.90, 1.06) | 0.73 | 1.32 (1.17, 1.45) | 0.0005 | 1.13 (0.96, 1.45) | 0.15 |

**Supplementary Table S13:** **Mendelian randomization estimates for the associations of genetically predicted circulating IGF-I concentrations risks of overall, aggressive and early-onset prostate cancer after Steiger filtering**

1 SD estimates based on UK Biobank males.

^*^Aggressive disease was defined as Gleason Score 8+, death from prostate cancer, metastatic disease, or PSA>100 ng/mL.

†Early-onset defined as diagnosed aged ≤ 55 years.

Abbreviations: CI=confidence interval; IGF-I=insulin-like growth factor-I; MR=Mendelian randomization; OR=odds ratio; PRESSO=pleiotropy residual sum and outlier; PSA=prostate-specific antigen; RAPS=robust adjusted profile score; SD=standard deviation.


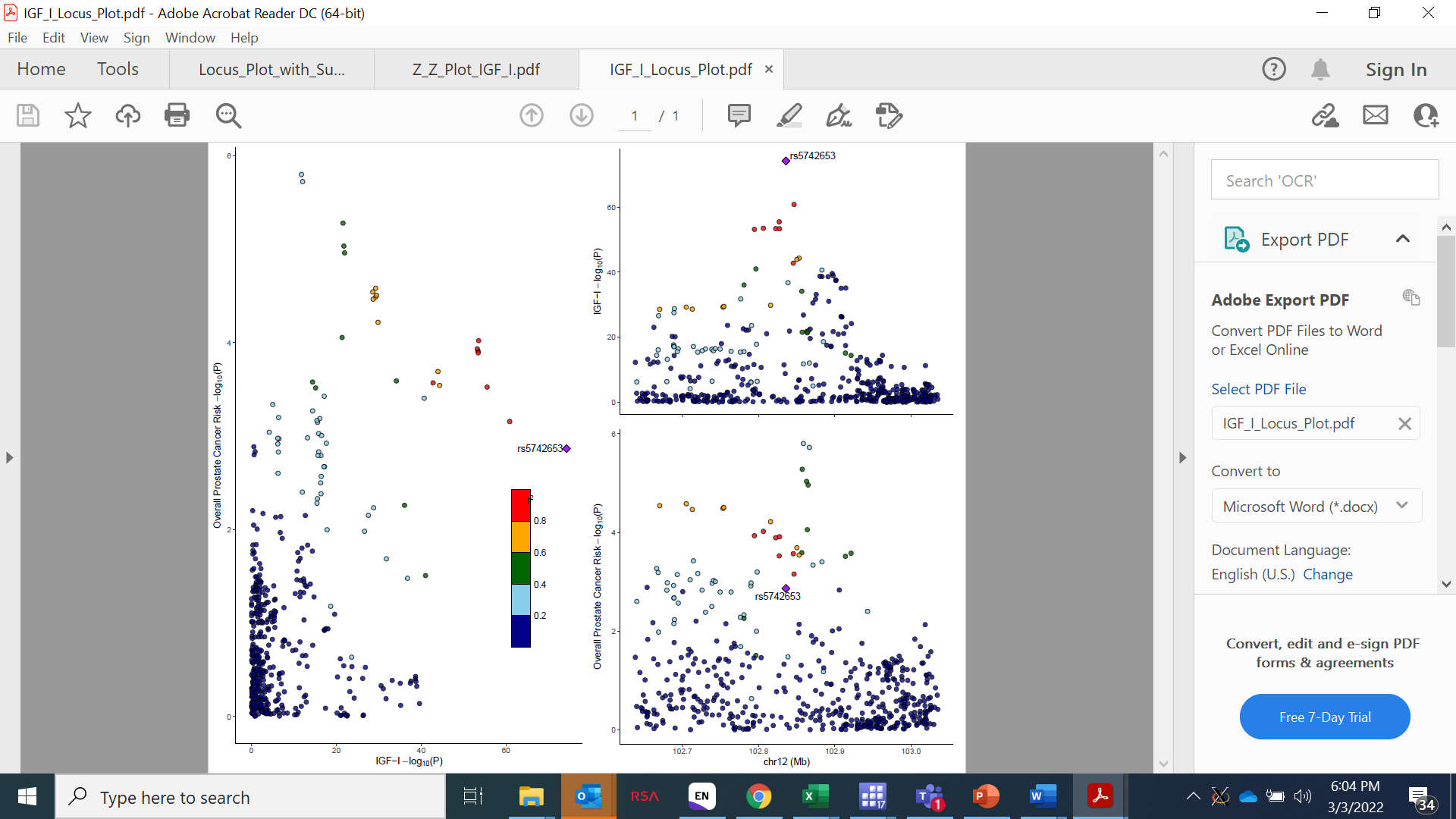


**A**

**B**


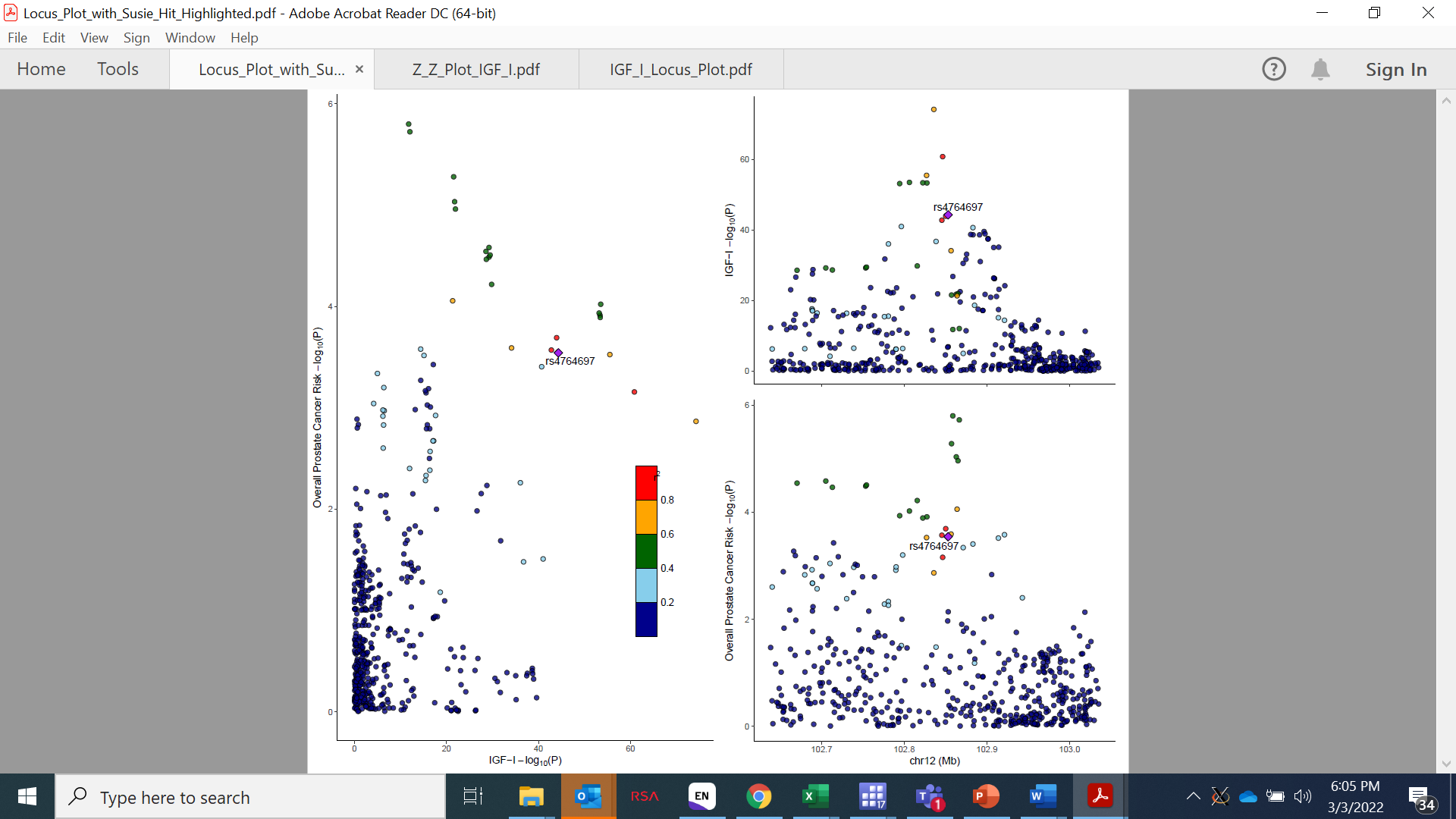


**Supplementary Figure S1: Colocalization of circulating IGF-I concentrations and overall prostate cancer risk**

Plots show the strength of the genetic associations with IGF-I, centered on the lead IGF-I *cis-*SNP (rs5742653) with overall prostate cancer risk. Each data point represents a SNP, and the colours indicate linkage disequilibrium (r^2^) with this lead SNP (represented by the purple diamond).

1. Highlighted lead *cis-* SNP (rs5742653)
2. Highlighted shared variant identified by SuSiE (rs4764697)

Abbreviations: IGF-I=insulin-like growth factor-I, SNP=single nucleotide polymorphism, SuSiE=Sum of Single Effects


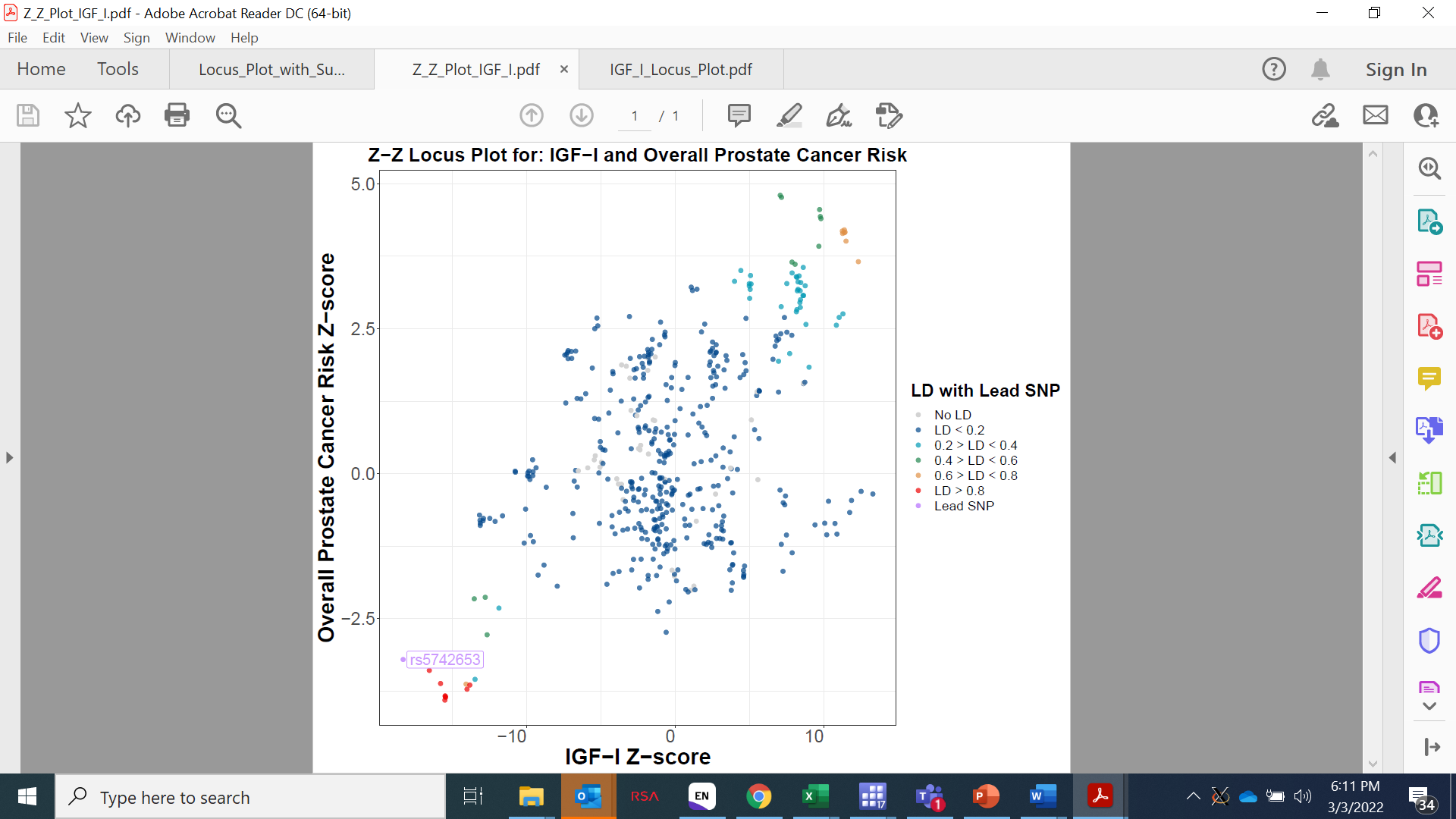


**Supplementary Figure S2: Z-Z plot of IGF-I and overall prostate cancer risk**

Abbreviations: IGF-I=insulin-like growth factor-I, SNP=single nucleotide polymorphism

**Supplementary Figure S3:** Odds ratio (95% CIs) for overall prostate cancer per study-specific 1 SD increment of IGF-II concentration by subgroup

Estimates are from logistic regression conditioned on the matching variables and adjusted for age, BMI, height, alcohol intake, smoking status, marital status, education status, racial/ethnic group, and diabetes status. The position of each square indicates the magnitude of the odds ratio, and the area of the square is proportional to the amount of statistical information available (inverse of the variance of the logarithm of the relative risk). The length of the horizontal line through the square indicates the 95% confidence interval. Tests for heterogeneity for case-defined factors were obtained by fitting separate models for each subgroup and assuming independence of the ORs using a method analogous to a meta-analysis. Tests for heterogeneity for non–case-defined factors were assessed with a χ2 test of interaction between subgroup and the binary variable

*Aggressive cancer defined as Gleason grade 8+, or prostate cancer death, or metastases or PSA >100 ng/mL

†Localized defined as TNM stage <T2 with no reported lymph node involvement or metastases or stage I; other localized stage if TNM stage T2 with no reported lymph node involvement or metastases, stage II, or equivalent; advanced stage if they were TNM stage T3 or T4 and/or N1+ and/or M1, stage III–IV, or equivalent.

‡ Low grade defined as Gleason score was <7 or equivalent (i.e. extent of differentiation good, moderate); medium grade if Gleason score was 7 (i.e. poorly differentiated); high grade if the Gleason score was ≥8 or equivalent (i.e. undifferentiated).

Abbreviations: BMI=body mass index; CI=confidence interval; IGF-II=insulin-like growth factor-II; OR=odds ratio; PSA=prostate-specific antigen; SD=standard deviation; TNM=tumor, node, metastases.

**Supplementary Figure S4:** Odds ratio (95% CIs) for aggressive* prostate cancer per study-specific 1 SD increment of IGF-II concentration by subgroup

Estimates are from logistic regression conditioned on the matching variables and adjusted for age, BMI, height, alcohol intake, smoking status, marital status, education status, racial/ethnic group, and diabetes status. The position of each square indicates the magnitude of the odds ratio, and the area of the square is proportional to the amount of statistical information available (inverse of the variance of the logarithm of the relative risk). The length of the horizontal line through the square indicates the 95% confidence interval. Tests for heterogeneity for case-defined factors were obtained by fitting separate models for each subgroup and assuming independence of the ORs using a method analogous to a meta-analysis. Tests for heterogeneity for non–case-defined factors were assessed with a χ2 test of interaction between subgroup and the binary variable

*Aggressive cancer defined as Gleason grade 8+, or prostate cancer death, or metastases or PSA >100 ng/mL

†Localized defined as TNM stage <T2 with no reported lymph node involvement or metastases or stage I, or TNM stage T2 with no reported lymph node involvement or metastases, stage II, or equivalent; advanced stage if they were TNM stage T3 or T4 and/or N1+ and/or M1, stage III–IV, or equivalent. Men with localized aggressive disease will have had high-grade prostate cancer, PSA > 100 ng/mL or died from prostate cancer.

‡ Low grade defined as Gleason score was <8 or equivalent (i.e., extent of differentiation good, moderate, poor); high grade if the Gleason score was ≥8 or equivalent (i.e., undifferentiated). Men with low-grade aggressive disease will have had advanced prostate cancer, PSA > 100 ng/mL or died from prostate cancer.

Abbreviations: BMI=body mass index; CI=confidence interval; IGF-II=insulin-like growth factor-II; OR=odds ratio; PSA=prostate-specific antigen; SD=standard deviation; TNM=tumor, node, metastases.

**Supplementary Figure S5:** Odds ratio (95% CIs) for overall prostate cancer per study-specific 1 SD increment of IGFBP-1 concentration by subgroup

Estimates are from logistic regression conditioned on the matching variables and adjusted for age, BMI, height, alcohol intake, smoking status, marital status, education status, racial/ethnic group, and diabetes status. The position of each square indicates the magnitude of the odds ratio, and the area of the square is proportional to the amount of statistical information available (inverse of the variance of the logarithm of the relative risk). The length of the horizontal line through the square indicates the 95% confidence interval. Tests for heterogeneity for case-defined factors were obtained by fitting separate models for each subgroup and assuming independence of the ORs using a method analogous to a meta-analysis. Tests for heterogeneity for non–case-defined factors were assessed with a χ2 test of interaction between subgroup and the binary variable

*Aggressive cancer defined as Gleason grade 8+, or prostate cancer death, or metastases or PSA >100 ng/mL

†Localized defined as TNM stage <T2 with no reported lymph node involvement or metastases or stage I; other localized stage if TNM stage T2 with no reported lymph node involvement or metastases, stage II, or equivalent; advanced stage if they were TNM stage T3 or T4 and/or N1+ and/or M1, stage III–IV, or equivalent.

‡ Low grade defined as Gleason score was <7 or equivalent (i.e. extent of differentiation good, moderate); medium grade if Gleason score was 7 (i.e. poorly differentiated); high grade if the Gleason score was ≥8 or equivalent (i.e. undifferentiated).

Abbreviations: BMI=body mass index; CI=confidence interval; IGFBP-1=insulin-like growth factor binding protein-1; OR=odds ratio; PSA=prostate-specific antigen; SD=standard deviation; TNM=tumor, node, metastases.

**Supplementary Figure S6:** Odds ratio (95% CIs) for aggressive* prostate cancer per study-specific 1 SD increment of IGFBP-1 concentration by subgroup

Estimates are from logistic regression conditioned on the matching variables and adjusted for age, BMI, height, alcohol intake, smoking status, marital status, education status, racial/ethnic group, and diabetes status. The position of each square indicates the magnitude of the odds ratio, and the area of the square is proportional to the amount of statistical information available (inverse of the variance of the logarithm of the relative risk). The length of the horizontal line through the square indicates the 95% confidence interval. Tests for heterogeneity for case-defined factors were obtained by fitting separate models for each subgroup and assuming independence of the ORs using a method analogous to a meta-analysis. Tests for heterogeneity for non–case-defined factors were assessed with a χ2 test of interaction between subgroup and the binary variable

*Aggressive cancer defined as Gleason grade 8+, or prostate cancer death, or metastases or PSA >100 ng/mL

†Localized defined as TNM stage <T2 with no reported lymph node involvement or metastases or stage I, or TNM stage T2 with no reported lymph node involvement or metastases, stage II, or equivalent; advanced stage if they were TNM stage T3 or T4 and/or N1+ and/or M1, stage III–IV, or equivalent. Men with localized aggressive disease will have had high-grade prostate cancer, PSA > 100 ng/mL or died from prostate cancer.

‡ Low grade defined as Gleason score was <8 or equivalent (i.e., extent of differentiation good, moderate, poor); high grade if the Gleason score was ≥8 or equivalent (i.e., undifferentiated). Men with low-grade aggressive disease will have had advanced prostate cancer, PSA > 100 ng/mL or died from prostate cancer.

Abbreviations: BMI=body mass index; CI=confidence interval; IGFBP-1=insulin-like growth factor binding protein-1; OR=odds ratio; PSA=prostate-specific antigen; SD=standard deviation; TNM=tumor, node, metastases.

**Supplementary Figure S7:** Odds ratio (95% CIs) for overall prostate cancer per study-specific 1 SD increment of IGFBP-2 concentration by subgroup

Estimates are from logistic regression conditioned on the matching variables and adjusted for age, BMI, height, alcohol intake, smoking status, marital status, education status, racial/ethnic group, and diabetes status. The position of each square indicates the magnitude of the odds ratio, and the area of the square is proportional to the amount of statistical information available (inverse of the variance of the logarithm of the relative risk). The length of the horizontal line through the square indicates the 95% confidence interval. Tests for heterogeneity for case-defined factors were obtained by fitting separate models for each subgroup and assuming independence of the ORs using a method analogous to a meta-analysis. Tests for heterogeneity for non–case-defined factors were assessed with a χ2 test of interaction between subgroup and the binary variable.

*Aggressive cancer defined as Gleason grade 8+, or prostate cancer death, or metastases or PSA >100 ng/mL

†Localized defined as TNM stage <T2 with no reported lymph node involvement or metastases or stage I; other localized stage if TNM stage T2 with no reported lymph node involvement or metastases, stage II, or equivalent; advanced stage if they were TNM stage T3 or T4 and/or N1+ and/or M1, stage III–IV, or equivalent.

‡ Low grade defined as Gleason score was <7 or equivalent (i.e. extent of differentiation good, moderate); medium grade if Gleason score was 7 (i.e. poorly differentiated); high grade if the Gleason score was ≥8 or equivalent (i.e. undifferentiated).

Abbreviations: BMI=body mass index; CI=confidence interval; IGFBP-2=insulin-like growth factor binding protein-2; OR=odds ratio; PSA=prostate-specific antigen; SD=standard deviation; TNM=tumor, node, metastases.

**Supplementary Figure S8:** Odds ratio (95% CIs) for aggressive prostate cancer* per study-specific 1 SD increment of IGFBP-2 concentration by subgroup

Estimates are from logistic regression conditioned on the matching variables and adjusted for age, BMI, height, alcohol intake, smoking status, marital status, education status, racial/ethnic group, and diabetes status. The position of each square indicates the magnitude of the odds ratio, and the area of the square is proportional to the amount of statistical information available (inverse of the variance of the logarithm of the relative risk). The length of the horizontal line through the square indicates the 95% confidence interval. Tests for heterogeneity for case-defined factors were obtained by fitting separate models for each subgroup and assuming independence of the ORs using a method analogous to a meta-analysis. Tests for heterogeneity for non–case-defined factors were assessed with a χ2 test of interaction between subgroup and the binary variable

*Aggressive cancer defined as Gleason grade 8+, or prostate cancer death, or metastases or PSA >100 ng/mL

†Localized defined as TNM stage <T2 with no reported lymph node involvement or metastases or stage I, or TNM stage T2 with no reported lymph node involvement or metastases, stage II, or equivalent; advanced stage if they were TNM stage T3 or T4 and/or N1+ and/or M1, stage III–IV, or equivalent. Men with localized aggressive disease will have had high-grade prostate cancer, PSA > 100 ng/mL or died from prostate cancer.

‡ Low grade defined as Gleason score was <8 or equivalent (i.e., extent of differentiation good, moderate, poor); high grade if the Gleason score was ≥8 or equivalent (i.e., undifferentiated). Men with low-grade aggressive disease will have had advanced prostate cancer, PSA > 100 ng/mL or died from prostate cancer.

Abbreviations: BMI=body mass index; CI=confidence interval; IGFBP-2=insulin-like growth factor binding protein-2; OR=odds ratio; PSA=prostate-specific antigen; SD=standard deviation.

**Supplementary Figure S9:** Odds ratio (95% CIs) for overall prostate cancer per study-specific 1 SD increment of IGFBP-3 concentration by subgroup

Estimates are from logistic regression conditioned on the matching variables and adjusted for age, BMI, height, alcohol intake, smoking status, marital status, education status, racial/ethnic group, and diabetes status. The position of each square indicates the magnitude of the odds ratio, and the area of the square is proportional to the amount of statistical information available (inverse of the variance of the logarithm of the relative risk). The length of the horizontal line through the square indicates the 95% confidence interval. Tests for heterogeneity for case-defined factors were obtained by fitting separate models for each subgroup and assuming independence of the ORs using a method analogous to a meta-analysis. Tests for heterogeneity for non–case-defined factors were assessed with a χ2 test of interaction between subgroup and the binary variable

*Aggressive cancer defined as Gleason grade 8+, or prostate cancer death, or metastases or PSA >100 ng/mL

†Localized defined as TNM stage <T2 with no reported lymph node involvement or metastases or stage I; other localized stage if TNM stage T2 with no reported lymph node involvement or metastases, stage II, or equivalent; advanced stage if they were TNM stage T3 or T4 and/or N1+ and/or M1, stage III–IV, or equivalent.

‡ Low grade defined as Gleason score was <7 or equivalent (i.e. extent of differentiation good, moderate); medium grade if Gleason score was 7 (i.e. poorly differentiated); high grade if the Gleason score was ≥8 or equivalent (i.e. undifferentiated).

Abbreviations: BMI=body mass index; CI=confidence interval; IGFBP-3=insulin-like growth factor binding protein-2; OR=odds ratio; PSA=prostate-specific antigen; SD=standard deviation.

**Supplementary Figure S10:** Odds ratio (95% CIs) for aggressive* prostate cancer per study-specific 1 SD increment of IGFBP-3 concentration by subgroup

Estimates are from logistic regression conditioned on the matching variables and adjusted for age, BMI, height, alcohol intake, smoking status, marital status, education status, racial/ethnic group, and diabetes status. The position of each square indicates the magnitude of the odds ratio, and the area of the square is proportional to the amount of statistical information available (inverse of the variance of the logarithm of the relative risk). The length of the horizontal line through the square indicates the 95% confidence interval. Tests for heterogeneity for case-defined factors were obtained by fitting separate models for each subgroup and assuming independence of the ORs using a method analogous to a meta-analysis. Tests for heterogeneity for non–case-defined factors were assessed with a χ2 test of interaction between subgroup and the binary variable

*Aggressive cancer defined as Gleason grade 8+, or prostate cancer death, or metastases or PSA >100 ng/mL

†Localized defined as TNM stage <T2 with no reported lymph node involvement or metastases or stage I, or TNM stage T2 with no reported lymph node involvement or metastases, stage II, or equivalent; advanced stage if they were TNM stage T3 or T4 and/or N1+ and/or M1, stage III–IV, or equivalent. Men with localized aggressive disease will have had high-grade prostate cancer, PSA > 100 ng/mL or died from prostate cancer.

‡ Low grade defined as Gleason score was <8 or equivalent (i.e., extent of differentiation good, moderate, poor); high grade if the Gleason score was ≥8 or equivalent (i.e., undifferentiated). Men with low-grade aggressive disease will have had advanced prostate cancer, PSA > 100 ng/mL or died from prostate cancer.

Abbreviations: BMI=body mass index; CI=confidence interval; IGFBP-3=insulin-like growth factor binding protein-3; OR=odds ratio; PSA=prostate-specific antigen; SD=standard deviation.

**Supplementary Figure S11:** Odds ratios (95% confidence intervals) for prostate cancer associated with a 1 SD increment in IGF-I by study

Estimates are from logistic regression conditioned on the matching variables and adjusted for age, BMI, height, alcohol intake, smoking status, marital status, education status, racial/ethnic group, and diabetes status. Heterogeneity in linear trends between studies was tested by comparing the $\chi^{2}$ values for models with and without a (studies) × (linear trend) interaction term.

Test of significance (overall): p < 0.0001.

Test of heterogeneity (overall): $\chi_{24}^{2}$=29; p=0.23.

Test of significance (studies without organised screening): p < 0.001.

Test of heterogeneity between studies without organised screening: $\chi_{21}^{2}$= 24; p = 0.27.

Test of significance (studies with organised screening): p = 0.46.

Test of heterogeneity between studies with organised screening: $\chi_{2}^{2}$ = 0.57; p = 0.75.

Test of heterogeneity between studies with and without organised screening: $\chi_{1}^{2}$ = 3.9; p = 0.05.

Abbreviations: ATBC=The Alpha-Tocopherol, Beta-Carotene Cancer Prevention Study; BLSA=The Baltimore Longitudinal Study of Aging; BUPA=British United Provident Association; CHDS=Child Health and Development Studies; CHS=Cardiovascular Health Study; CI=confidence interval; CLUE=Give Us a Clue to Cancer and Heart Disease; EPIC=European Prospective Investigation into Cancer and Nutrition; ERSPC=European Randomized study of Screening for Prostate Cancer; HIMS=Health In Men Study; HPFS= Health Professionals Follow-up Study; IGF-I=insulin-like growth factor-I; KPMCP=Kaiser Permanente Medical Care Program; JACC=Japan Collaborative Cohort Study; MCCS=Melbourne Collaborative Cohort Study; MEC= Multiethnic Cohort Study of Diet and Cancer; NSHDC=Northern Sweden Health and Disease Cohort; OR=odds ratio; PCPT= Prostate Cancer Prevention Trial; PHS=Physicians' Health Study; PLCO= Prostate, Lung, Colorectal and Ovarian Cancer Screening Trial; SD=standard deviation; SU.VI.MAX=Supplémentation en Vitamines et Minéraux Antioxydants.

**Supplementary Figure S12:** Odds ratios (95% confidence intervals) for aggressive* prostate cancer associated with a 1 SD increment in IGF-I by study

Estimates are from logistic regression conditioned on the matching variables and adjusted for age, BMI, height, alcohol intake, smoking status, marital status, education status, racial/ethnic group, and diabetes status. Heterogeneity in linear trends between studies was tested by comparing the $\chi^{2}$ values for models with and without a (studies) × (linear trend) interaction term.

Test of significance (overall): p=0.005.

Test of heterogeneity (overall): $\chi_{24}^{2}$=41; p=0.02.

Test of significance (studies without organised screening): p=0.01.

Test of heterogeneity between studies without organised screening: $\chi_{21}^{2}$= 31; p = 0.08.

Test of significance (studies with organised screening): p = 0.26.

Test of heterogeneity between studies with organised screening: $\chi_{2}^{2}$ = 13; p = 0.002.

Test of heterogeneity between studies with and without organised screening: $\chi_{1}^{2}$ = 0.52; p = 0.47.

*Aggressive cancer defined as Gleason grade 8+, or prostate cancer death, or metastases or PSA>100 ng/mL

Abbreviations: ATBC=The Alpha-Tocopherol, Beta-Carotene Cancer Prevention Study; BLSA=The Baltimore Longitudinal Study of Aging; BUPA=British United Provident Association; CHDS=Child Health and Development Studies; CHS=Cardiovascular Health Study; CI=confidence interval; CLUE=Give Us a Clue to Cancer and Heart Disease; EPIC=European Prospective Investigation into Cancer and Nutrition; ERSPC=European Randomized study of Screening for Prostate Cancer; HIMS=Health In Men Study; HPFS= Health Professionals Follow-up Study; IGF-I=insulin-like growth factor-I; KPMCP=Kaiser Permanente Medical Care Program; JACC=Japan Collaborative Cohort Study; MCCS=Melbourne Collaborative Cohort Study; MEC= Multiethnic Cohort Study of Diet and Cancer; NSHDC=Northern Sweden Health and Disease Cohort; OR=odds ratio; PCPT= Prostate Cancer Prevention Trial; PHS=Physicians' Health Study; PLCO= Prostate, Lung, Colorectal and Ovarian Cancer Screening Trial; SD=standard deviation; SU.VI.MAX=Supplémentation en Vitamines et Minéraux Antioxydants.

**Supplementary Figure S13:** Odds ratios (95% confidence intervals) for overall prostate cancer associated with a 1 SD increment in IGF-II by study

Estimates are from logistic regression conditioned on the matching variables and adjusted for age, BMI, height, alcohol intake, smoking status, marital status, education status, racial/ethnic group, and diabetes status. Heterogeneity in linear trends between studies was tested by comparing the $\chi^{2}$ values for models with and without a (studies) × (linear trend) interaction term.

Test of significance (overall): p = 0.008.

Test of heterogeneity (overall): $\chi_{10}^{2}$=35; p=0.0001.

Test of significance (studies without organised screening): p = 0.004.

Test of heterogeneity between studies without organised screening: $\chi_{9}^{2}$= 30; p = 0.0004.

Test of significance (studies with organised screening): p = 0.68.

Test of heterogeneity between studies with and without organised screening: $\chi_{1}^{2}$ = 0.77; p = 0.38.

Abbreviations: BLSA= The Baltimore Longitudinal Study of Aging; BUPA=British United Provident Association; CI=confidence interval; CLUE=Give Us a Clue to Cancer and Heart Disease; EPIC=European Prospective Investigation into Cancer and Nutrition; IGF-II=insulin-like growth factor-II; JACC=Japan Collaborative Cohort Study; MEC=Multiethnic Cohort Study of Diet and Cancer; OR=odds ratio; PCPT= Prostate Cancer Prevention Trial; PHS=Physicians' Health Study; SD=standard deviation; SU.VI.MAX=Supplémentation en Vitamines et Minéraux Antioxydants.

**Supplementary Figure S14:** Odds ratios (95% confidence intervals) for aggressive* prostate cancer associated with a 1 SD increment in IGF-II by study

Estimates are from logistic regression conditioned on the matching variables and adjusted for age, BMI, height, alcohol intake, smoking status, marital status, education status, racial/ethnic group, and diabetes status. Heterogeneity in linear trends between studies was tested by comparing the $\chi^{2}$ values for models with and without a (studies) × (linear trend) interaction term.

Test of significance (overall): p = 0.16.

Test of heterogeneity (overall): $\chi_{10}^{2}$=6.8; p=0.75.

Test of significance (studies without organised screening): p = 0.16.

Test of heterogeneity between studies without organised screening: $\chi_{9}^{2}$= 7.2; p = 0.62.

Test of significance (studies with organised screening): p = 0.19.

Test of heterogeneity between studies with and without organised screening: $\chi_{1}^{2}$ = 0.03; p = 0.86.

*Aggressive cancer defined as Gleason grade 8+, or prostate cancer death, or metastases or PSA>100 ng/mL

Abbreviations: BLSA= The Baltimore Longitudinal Study of Aging; BUPA=British United Provident Association; CI=confidence interval; CLUE=Give Us a Clue to Cancer and Heart Disease; EPIC=European Prospective Investigation into Cancer and Nutrition; IGF-II=insulin-like growth factor-II; JACC=Japan Collaborative Cohort Study; MEC=Multiethnic Cohort Study of Diet and Cancer; OR=odds ratio; PCPT= Prostate Cancer Prevention Trial; PHS=Physicians' Health Study; SD=standard deviation; SU.VI.MAX=Supplémentation en Vitamines et Minéraux Antioxydants.

**Supplementary Figure S15:** Odds ratios (95% confidence intervals) for overall prostate cancer associated with a 1 SD increment in IGFBP-1 by study

Estimates are from logistic regression conditioned on the matching variables and adjusted for age, BMI, height, alcohol intake, smoking status, marital status, education status, racial/ethnic group, and diabetes status. Heterogeneity in linear trends between studies was tested by comparing the $\chi^{2}$ values for models with and without a (studies) × (linear trend) interaction term.

Test of significance (overall): p = 0.03.

Test of heterogeneity (overall): $\chi_{7}^{2}$=2.8; p=0.90.

Abbreviations: CI=confidence interval; CLUE=Give Us a Clue to Cancer and Heart Disease; EPIC=European Prospective Investigation into Cancer and Nutrition; HIMS=Health in Men Study; HPFS=Health Professional’s Follow-up Study; IGFBP-1=insulin-like growth factor binding protein-1; MEC=Multiethnic Cohort Study of Diet and Cancer; OR=odds ratio; PHS=Physicians' Health Study; SD=standard deviation.

**Supplementary Figure S16:** Odds ratios (95% confidence intervals) for aggressive* prostate cancer associated with a 1 SD increment in IGFBP-1 by study

Estimates are from logistic regression conditioned on the matching variables and adjusted for age, BMI, height, alcohol intake, smoking status, marital status, education status, racial/ethnic group, and diabetes status. Heterogeneity in linear trends between studies was tested by comparing the $\chi^{2}$ values for models with and without a (studies) × (linear trend) interaction term.

Test of significance (overall): p = 0.43.

Test of heterogeneity (overall): $\chi_{7}^{2}$=11; p=0.14.

*Aggressive cancer defined as Gleason grade 8+, or prostate cancer death, or metastases or PSA>100 ng/mL

Abbreviations: CI=confidence interval; CLUE=Give Us a Clue to Cancer and Heart Disease; EPIC=European Prospective Investigation into Cancer and Nutrition; HIMS=Health in Men Study; HPFS=Health Professional’s Follow-up Study; IGFBP-1=insulin-like growth factor binding protein-1; MEC=Multiethnic Cohort Study of Diet and Cancer; OR=odds ratio; PHS=Physicians' Health Study; SD=standard deviation.

**Supplementary Figure S17:** Odds ratios (95% confidence intervals) for overall prostate cancer associated with a 1 SD increment in IGFBP-2 by study

Estimates are from logistic regression conditioned on the matching variables and adjusted for age, BMI, height, alcohol intake, smoking status, marital status, education status, racial/ethnic group, and diabetes status. Heterogeneity in linear trends between studies was tested by comparing the $\chi^{2}$ values for models with and without a (studies) × (linear trend) interaction term.

Test of significance (overall): p = 0.46.

Test of heterogeneity (overall): $\chi_{4}^{2}$=12; p=0.02.

Test of significance (studies without organised screening): p = 0.04.

Test of heterogeneity between studies without organised screening: $\chi_{3}^{2}$= 0.03; p = 1.00.

Test of significance (studies with organised screening): p = 0.04.

Test of heterogeneity between studies with and without organised screening: $\chi_{1}^{2}$ = 12; p = 0.0007.

Abbreviations: CI=confidence interval; EPIC=European Prospective Investigation into Cancer and Nutrition; HIMS=Health in Men Study; HPFS=Health Professional’s Follow-up Study; IGFBP-2=insulin-like growth factor binding protein-2; NSHDC=Northern Sweden Health and Disease Cohort; OR=odds ratio; PCPT= Prostate Cancer Prevention Trial; SD=standard deviation; SU.VI.MAX=Supplémentation en Vitamines et Minéraux Antioxydants.

**Supplementary Figure S18:** Odds ratios (95% confidence intervals) for aggressive* prostate cancer associated with a 1 SD increment in IGFBP-2 by study

Estimates are from logistic regression conditioned on the matching variables and adjusted for age, BMI, height, alcohol intake, smoking status, marital status, education status, racial/ethnic group, and diabetes status. Heterogeneity in linear trends between studies was tested by comparing the $\chi^{2}$ values for models with and without a (studies) × (linear trend) interaction term.

Test of significance (overall): p = 0.21.

Test of heterogeneity (overall): $\chi_{4}^{2}$=4.4; p=0.35.

Test of significance (studies without organised screening): p = 0.14.

Test of heterogeneity between studies without organised screening: $\chi_{3}^{2}$= 4.3; p = 0.24.

Test of significance (studies with organised screening): p = 0.25.

Test of heterogeneity between studies with and without organised screening: $\chi_{1}^{2}$ = 0.55; p = 0.46.

*Aggressive cancer defined as Gleason grade 8+, or prostate cancer death, or metastases or PSA>100 ng/mL

Abbreviations: CI=confidence interval; EPIC=European Prospective Investigation into Cancer and Nutrition; HIMS=Health in Men Study; HPFS=Health Professional’s Follow-up Study; IGFBP-2=insulin-like growth factor binding protein-2; NSHDC=Northern Sweden Health and Disease Cohort; OR=odds ratio; PCPT= Prostate Cancer Prevention Trial; SD=standard deviation; SU.VI.MAX=Supplémentation en Vitamines et Minéraux Antioxydants.

**Supplementary Figure S19:** Odds ratios (95% confidence intervals) for prostate cancer associated with a 1 SD increment in IGFBP-3 by study

Estimates are from logistic regression conditioned on the matching variables and adjusted for age, BMI, height, alcohol intake, smoking status, marital status, education status, racial/ethnic group, and diabetes status. Heterogeneity in linear trends between studies was tested by comparing the $\chi^{2}$ values for models with and without a (studies) × (linear trend) interaction term.

Test of significance (overall): p<0.0001.

Test of heterogeneity (overall): $\chi_{19}^{2}$=29; p=0.07.

Test of significance (studies without organised screening): p < 0.0001.

Test of heterogeneity between studies without organised screening: $\chi_{16}^{2}$= 23; p = 0.11.

Test of significance (studies with organised screening): p = 0.99.

Test of heterogeneity between studies with organised screening: $\chi_{2}^{2}$ = 1.5; p = 0.46.

Test of heterogeneity between studies with and without organised screening: $\chi_{1}^{2}$ = 4.3; p = 0.04.

Abbreviations: ATBC=The Alpha-Tocopherol, Beta-Carotene Cancer Prevention Study; BLSA=The Baltimore Longitudinal Study of Aging; BUPA=British United Provident Association; CHS=Cardiovascular Health Study; CI=confidence interval; CLUE=Give Us a Clue to Cancer and Heart Disease; EPIC=European Prospective Investigation into Cancer and Nutrition; ERSPC=European Randomized study of Screening for Prostate Cancer; HIMS=Health In Men Study; HPFS= Health Professionals Follow-up Study; IGFBP-3=insulin-like growth factor binding protein-3; JACC=Japan Collaborative Cohort Study; MCCS=Melbourne Collaborative Cohort Study; MEC= Multiethnic Cohort Study of Diet and Cancer; NSHDC=Northern Sweden Health and Disease Cohort; OR=odds ratio; PCPT=Prostate Cancer Prevention Trial; PHS=Physicians' Health Study; PLCO= Prostate, Lung, Colorectal and Ovarian Cancer Screening Trial; SD=standard deviation; SU.VI.MAX=Supplémentation en Vitamines et Minéraux Antioxydants.

**Supplementary Figure S20:** Odds ratios (95% confidence intervals) for aggressive* prostate cancer associated with a 1 SD increment in IGFBP-3 by study

Estimates are from logistic regression conditioned on the matching variables and adjusted for age, BMI, height, alcohol intake, smoking status, marital status, education status, racial/ethnic group, and diabetes status. Heterogeneity in linear trends between studies was tested by comparing the $\chi^{2}$ values for models with and without a (studies) × (linear trend) interaction term.

Test of significance (overall): p=0.42.

Test of heterogeneity (overall): $\chi_{19}^{2}$=27; p=0.11.

Test of significance (studies without organised screening): p=0.32.

Test of heterogeneity between studies without organised screening: $\chi_{16}^{2}$= 26; p = 0.05.

Test of significance (studies with organised screening): p = 0.43.

Test of heterogeneity between studies with organised screening: $\chi_{2}^{2}$ = 0.3; p = 0.86.

Test of heterogeneity between studies with and without organised screening: $\chi_{1}^{2}$ = 0.43; p = 0.51.

*Aggressive cancer defined as Gleason grade 8+, or prostate cancer death, or metastases or PSA>100 ng/mL

Abbreviations: ATBC=The Alpha-Tocopherol, Beta-Carotene Cancer Prevention Study; BLSA=The Baltimore Longitudinal Study of Aging; BUPA=British United Provident Association; CHS=Cardiovascular Health Study; CI=confidence interval; CLUE=Give Us a Clue to Cancer and Heart Disease; EPIC=European Prospective Investigation into Cancer and Nutrition; ERSPC=European Randomized study of Screening for Prostate Cancer; HIMS=Health In Men Study; HPFS= Health Professionals Follow-up Study; IGFBP-3=insulin-like growth factor binding protein-3; JACC=Japan Collaborative Cohort Study; MCCS=Melbourne Collaborative Cohort Study; MEC= Multiethnic Cohort Study of Diet and Cancer; NSHDC=Northern Sweden Health and Disease Cohort; OR=odds ratio; PCPT=Prostate Cancer Prevention Trial; PHS=Physicians' Health Study; PLCO= Prostate, Lung, Colorectal and Ovarian Cancer Screening Trial; SD=standard deviation; SU.VI.MAX=Supplémentation en Vitamines et Minéraux Antioxydants.

**Supplementary Figure 7:** Risks of aggressive* prostate cancer by study-specific fifths of biomarker concentrations and 1 SD increment in the unadjusted model.

Estimates are from logistic regression conditioned on the matching variables and are not adjusted. The position of each square indicates the magnitude of the odds ratio, and the area of the square is proportional to the amount of statistical information available (inverse of the variance of the logarithm of the relative risk). The length of the horizontal line through the square indicates the 95% confidence interval.

*Aggressive cancer defined as Gleason grade 8+, or prostate cancer death, or metastases or PSA at diagnosis >100 ng/mL.

Abbreviations: BMI=body mass index; CI=confidence interval; IGF=insulin-like growth factor; IGFBP=insulin-like growth factor binding protein; OR=odds ratio; PSA=prostate-specific antigen; SD=standard deviation.

**Supplementary Figure S21:** Risks of overall and aggressive* prostate cancer by study-specific fifths of biomarker concentrations and 1 SD increment in the unadjusted model.

Estimates are from logistic regression conditioned on the matching variables. The position of each square indicates the magnitude of the odds ratio, and the area of the square is proportional to the amount of statistical information available (inverse of the variance of the logarithm of the relative risk). The length of the horizontal line through the square indicates the 95% confidence interval.

*Aggressive cancer defined as Gleason grade 8+, or prostate cancer death, or metastases or PSA>100 ng/mL.

†Early-onset defined as diagnosed ≤55 years.

Abbreviations: BMI=body mass index; CI=confidence interval; IGF=insulin-like growth factor; IGFBP=insulin-like growth factor binding protein; OR=odds ratio; PSA=prostate-specific antigen; SD=standard deviation.

**Supplementary Figure 8:** Risks of aggressive* prostate cancer by study-specific tenths of biomarker concentrations and 90%tile increment.

Estimates are from logistic regression conditioned on the matching variables and adjusted for age, BMI, height, alcohol intake, smoking status, marital status, education status, racial/ethnic group, and diabetes status. The categorical variables representing the study-specific tenths of the biomarker concentrations was replaced with a continuous variable that was scored as 0, 0.11, 0.22, 0.33, 0.44, 0.56 0.67, 0.78, 0.89 and 1; because the mid-points of the lowest and highest tenths are the 10^th^ and 90^th^ percentiles of the study-specific biomarker concentrations, a unit increase in this variable can be taken to represent an 90 percentile increase in the biomarker study-specific concentration. The position of each square indicates the magnitude of the odds ratio, and the area of the square is proportional to the amount of statistical information available (inverse of the variance of the logarithm of the relative risk). The length of the horizontal line through the square indicates the 95% confidence interval. *Aggressive cancer defined as Gleason grade 8+, or prostate cancer death, or metastases or PSA at diagnosis >100 ng/mL.

Abbreviations: BMI=body mass index; CI=confidence interval; IGF=insulin-like growth factor; IGFBP=insulin-like growth factor binding protein; OR=odds ratio; SHBG=sex hormone–binding globulin.

**Supplementary Figure S22:** Risks of aggressive* prostate cancer by study-specific tenths of biomarker concentrations and 90%tile increment.

Estimates are from logistic regression conditioned on the matching variables and adjusted for age, BMI, height, alcohol intake, smoking status, marital status, education status, racial/ethnic group, and diabetes status. The categorical variables representing the study-specific tenths of the biomarker concentrations was replaced with a continuous variable that was scored as 0, 0.11, 0.22, 0.33, 0.44, 0.56 0.67, 0.78, 0.89 and 1; because the mid-points of the lowest and highest tenths are the 10^th^ and 90^th^ percentiles of the study-specific biomarker concentrations, a unit increase in this variable can be taken to represent a 90 percentile increase in the biomarker study-specific concentration. The position of each square indicates the magnitude of the odds ratio, and the area of the square is proportional to the amount of statistical information available (inverse of the variance of the logarithm of the relative risk). The length of the horizontal line through the square indicates the 95% confidence interval.

*Aggressive cancer defined as Gleason grade 8+, or prostate cancer death, or metastases or PSA >100 ng/mL.

Abbreviations: BMI=body mass index; CI=confidence interval; IGF=insulin-like growth factor; IGFBP=insulin-like growth factor binding protein; OR=odds ratio; PSA=prostate specific antigen.


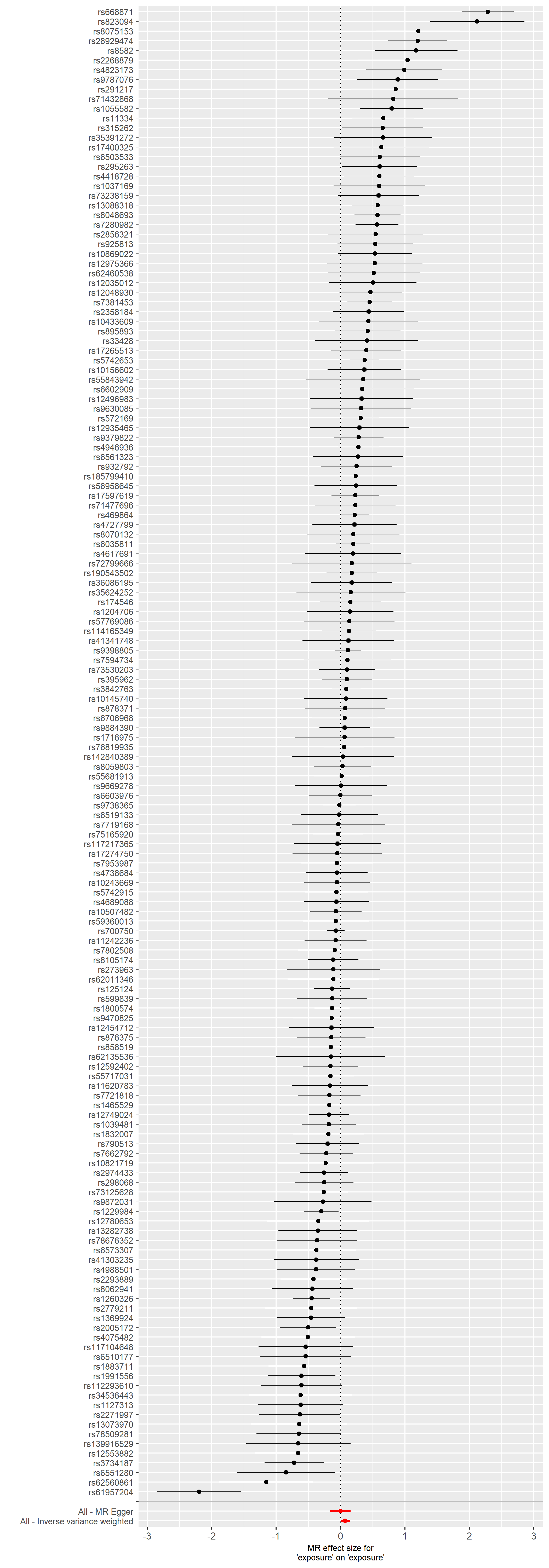


**Supplementary Figure S23: Forest plot of single SNP associations with overall prostate cancer**

Point estimates represent MR effect size, error bars represent 95% confidence intervals.

Abbreviations: IGF-I=insulin-like growth factor-1; MR=Mendelian randomization; SNP=single nucleotide polymorphism.


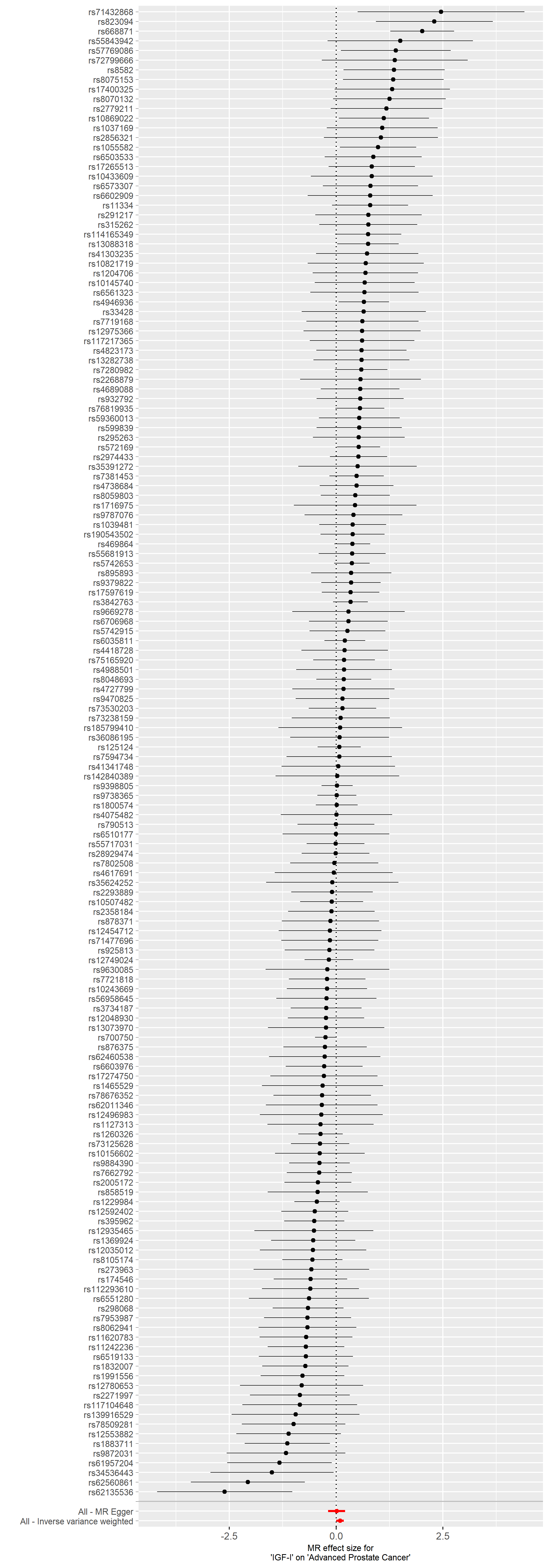


**Supplementary Figure S24: Forest plot of single SNP associations with aggressive* prostate cancer**

Point estimates represent MR effect size, error bars represent 95% confidence intervals.

*Aggressive cancer defined as Gleason grade 8+, or prostate cancer death, or metastases or PSA>100 ng/mL.

Abbreviations: IGF-I=insulin-like growth factor-1; MR=Mendelian randomization; PSA=prostate-specific antigen; SNP=single nucleotide polymorphism.


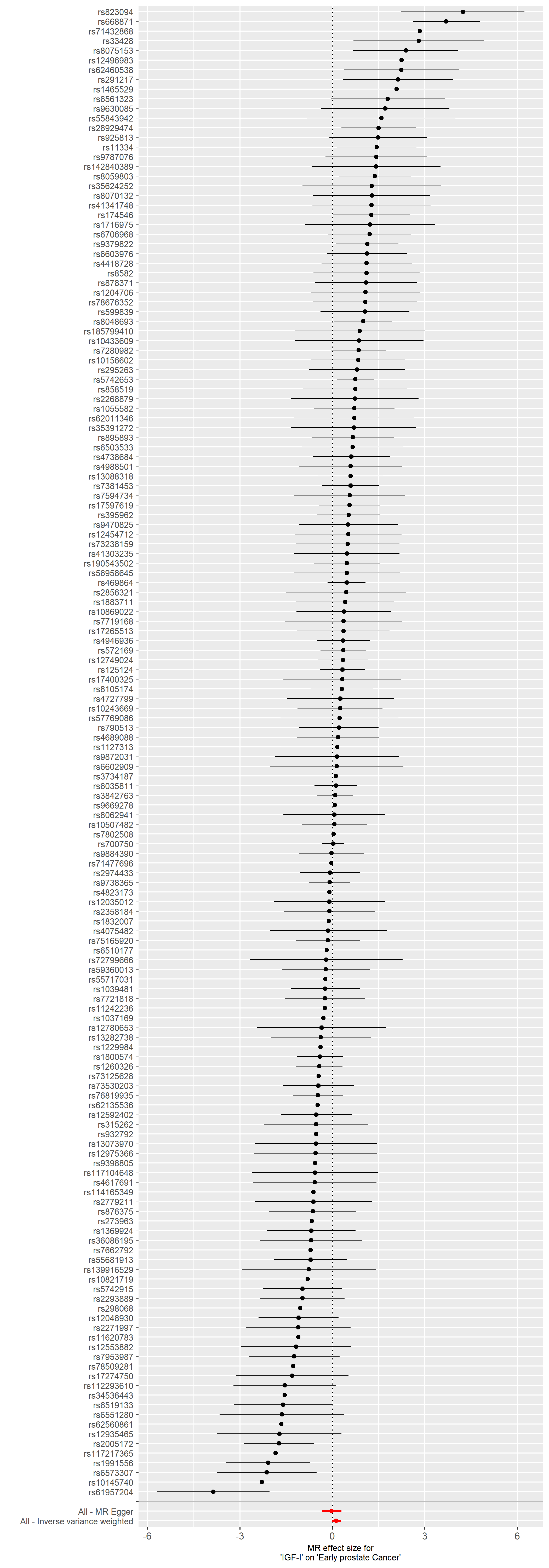


**Supplementary Figure S25: Forest plot of single SNP associations with early-onset* prostate cancer**

Point estimates represent MR effect size, error bars represent 95% confidence intervals.

*Early-onset defined as diagnosed ≤55 years.

Abbreviations: IGF-I=insulin-like growth factor-1; MR=Mendelian randomization; SNP=single nucleotide polymorphism.


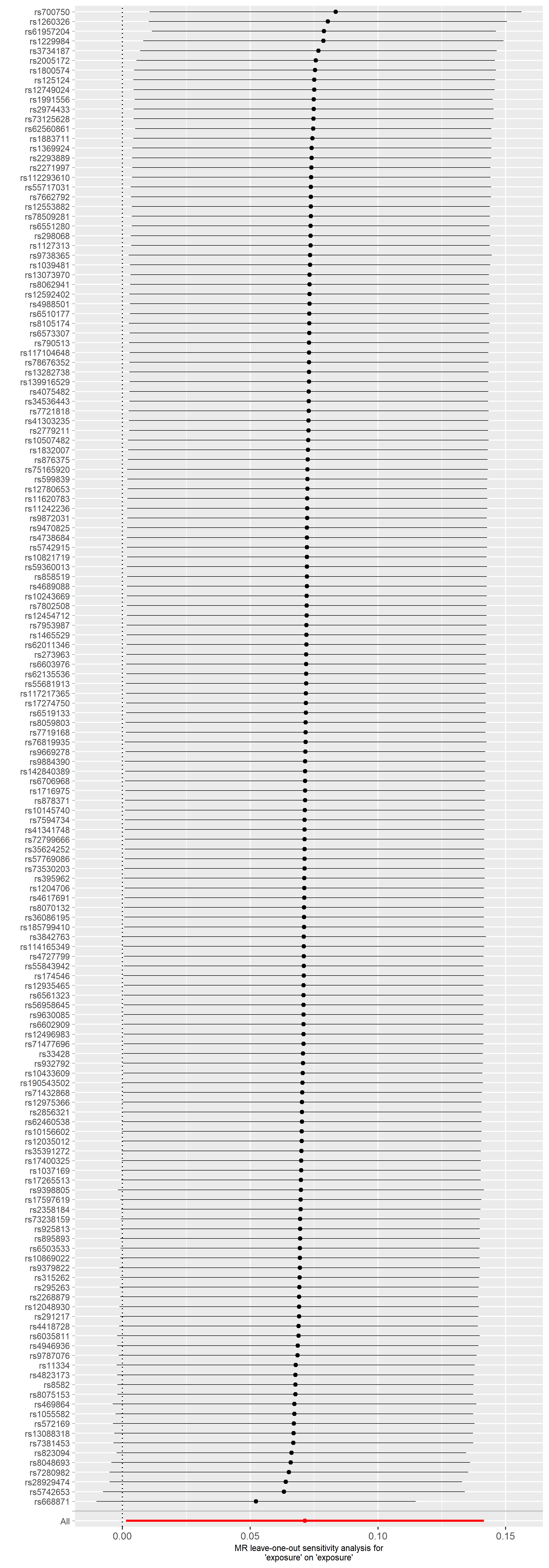


**Supplementary Figure S26: Leave-one-out plot of genetic associations with overall prostate cancer**

Point estimates represent MR effect size, error bars represent 95% confidence intervals.

Abbreviations: IGF-I=insulin-like growth factor-1; MR=Mendelian randomization.


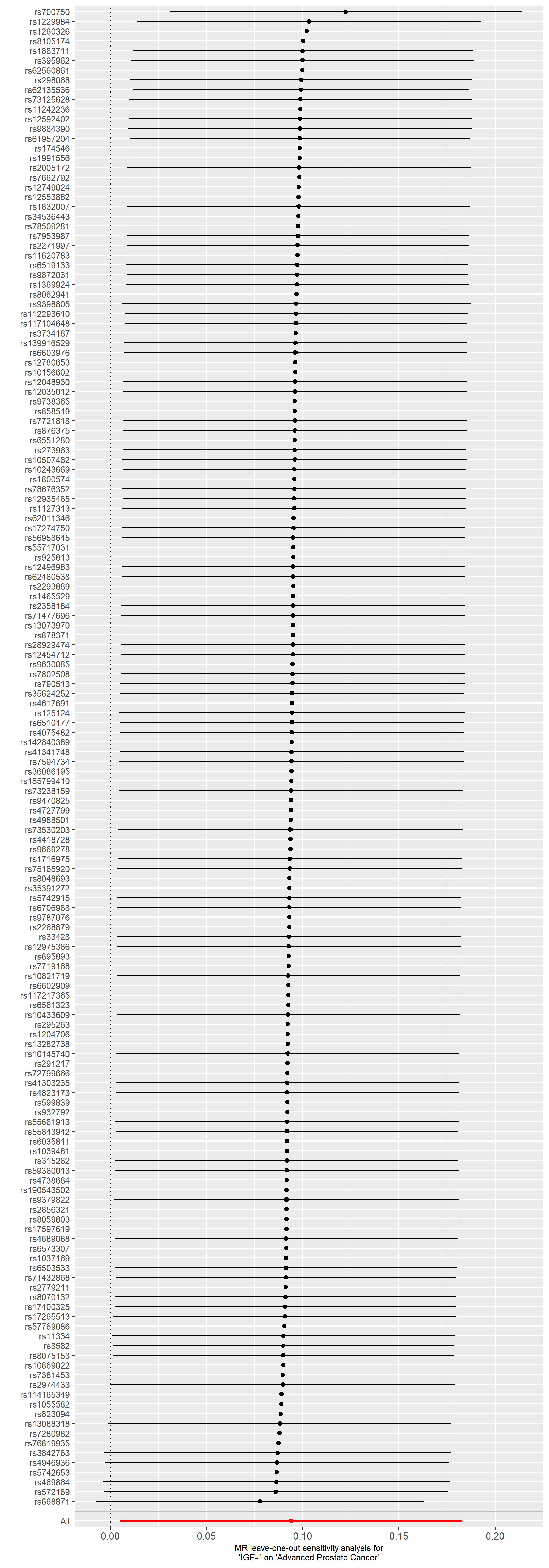


**Supplementary Figure S27: Leave-one-out plot of genetic associations with aggressive* prostate cancer**

Point estimates represent MR effect size, error bars represent 95% confidence intervals.

*Aggressive cancer defined as Gleason grade 8+, or prostate cancer death, or metastases or PSA>100 ng/mL.

Abbreviations: IGF-I=insulin-like growth factor-1; MR=Mendelian randomization; PSA=prostate-specific antigen; SNP=single nucleotide polymorphism.


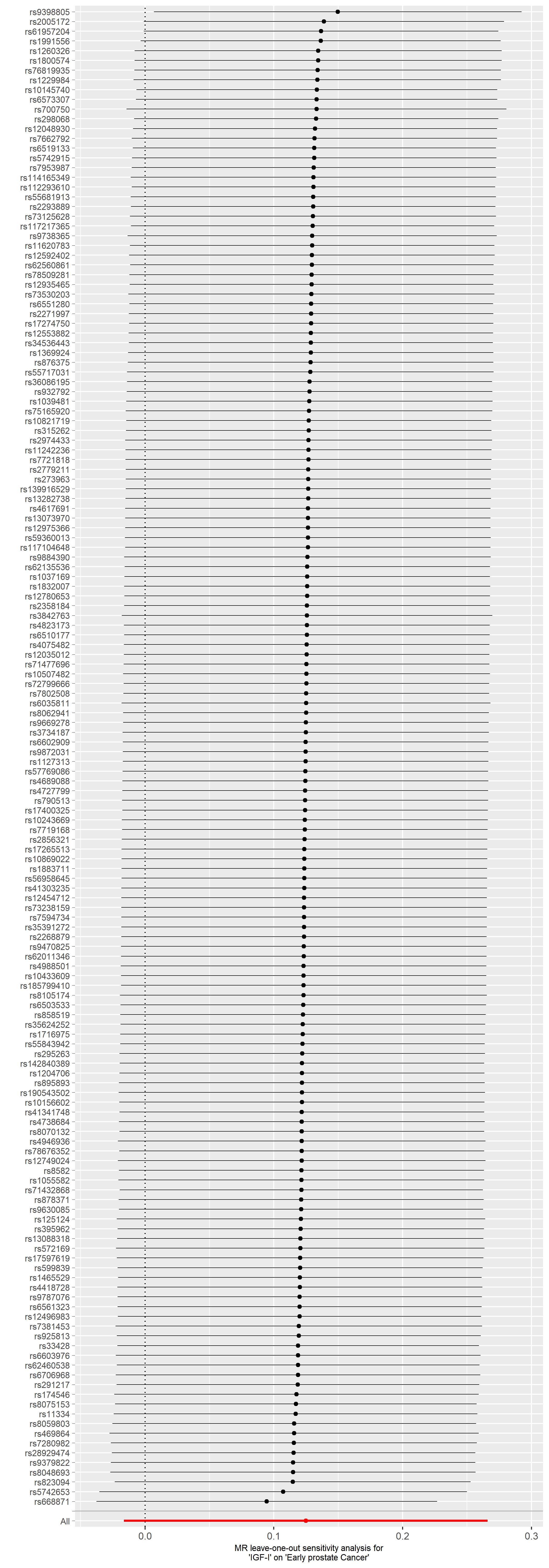


**Supplementary Figure S28: Leave-one-out plot of genetic associations with early-onset* prostate cancer**

Point estimates represent MR effect size, error bars represent 95% confidence intervals.

*Early-onset defined as diagnosed ≤55 years.

Abbreviations: IGF-I=insulin-like growth factor-1; MR=Mendelian randomization.


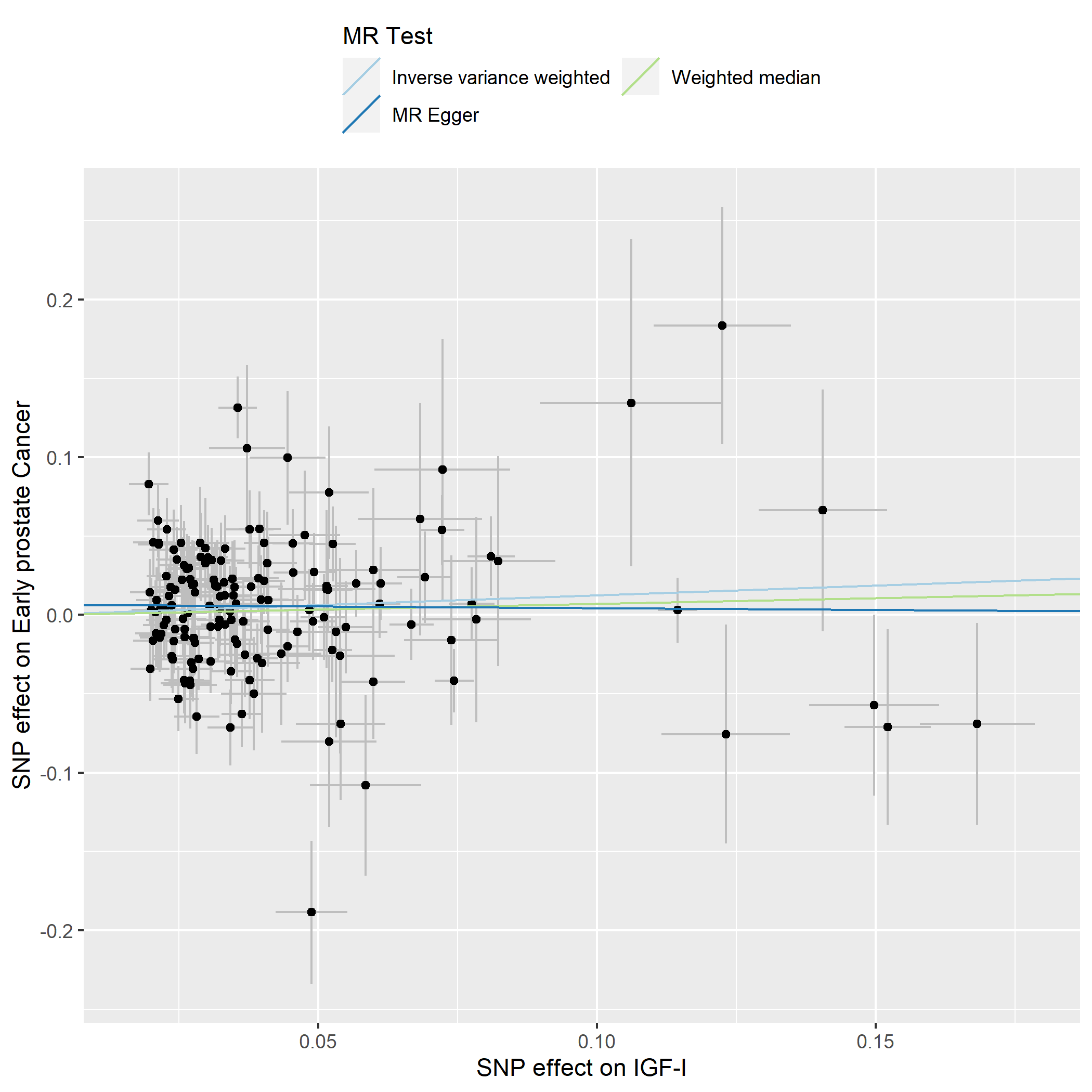

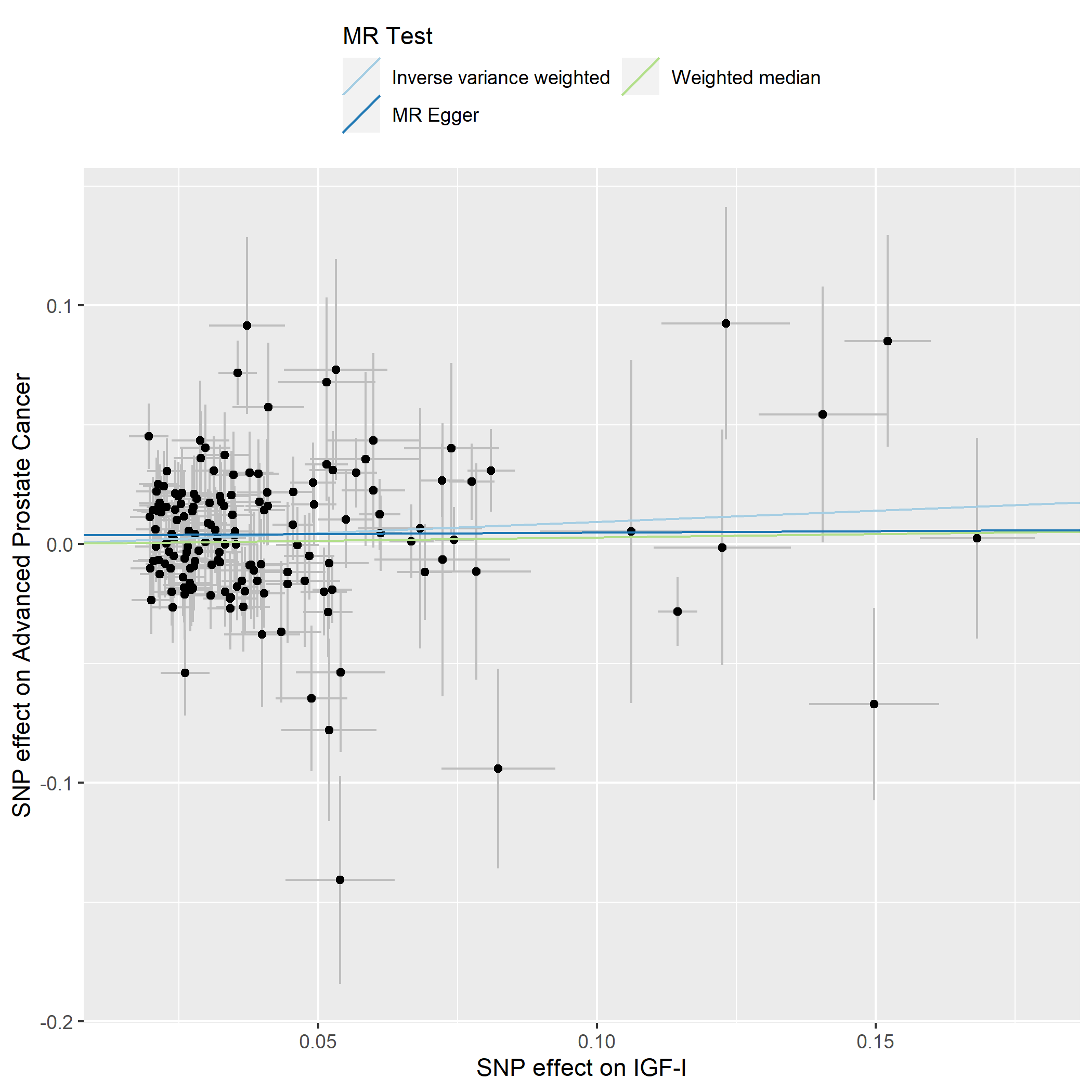

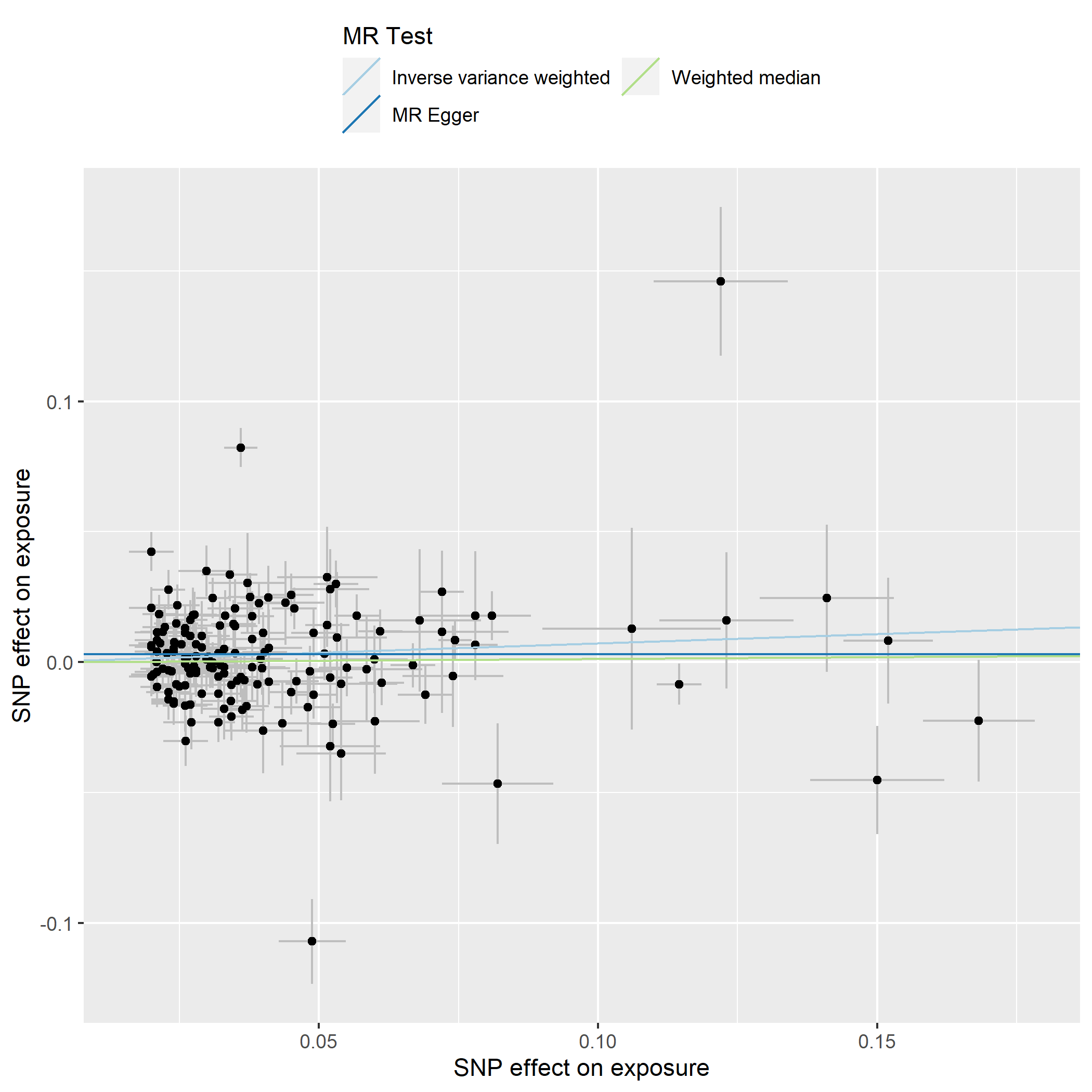


SNP effect on Aggressive Prostate Cancer

**SNP effect on IGF-I**

SNP effect on Overall Prostate Cancer

**B**

# Supplementary Figure S29: Scatterplot of genetic associations with IGF-I against genetic associations with:

# A) Overall prostate cancer

# B) Aggressive prostate cancer

# C) Early-onset prostate cancer

Aggressive cancer defined as Gleason grade 8+, or prostate cancer death, or metastases or PSA >100 ng/mL, early-onset defined as diagnosed ≤55 years. Point estimates represent log odds ratios. Error bars represent 95% confidence intervals, *cis*-SNP is coloured red.

Abbreviations: IGF-I=insulin-like growth factor-1; PSA=prostate-specific antigen; SNP=single nucleotide polymorphism.

**C**

**A**


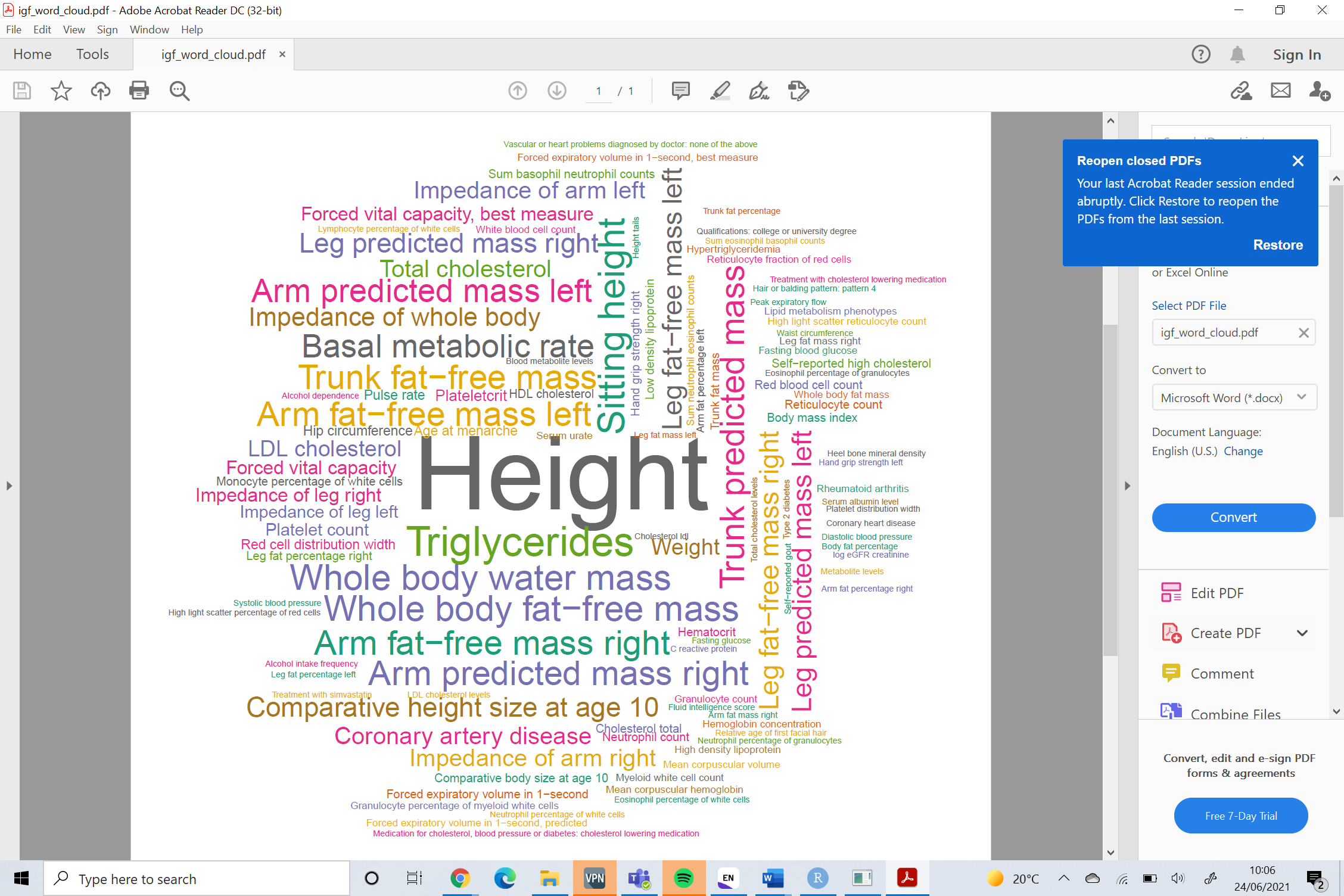


**Supplementary Figure S30:** Traits associated with genetically instrumented IGF-I

Traits were identified using PhenoScanner resource (1, 2). Larger words represent a greater frequency of the traits being associated with each SNP (P threshold=5 x 10^-8^). This figure was created using the wordcloud package in R.

Abbreviations: IGF-I=insulin-like growth factor-I; SNP=single nucleotide polymorphism.

**References**

1. Kamat MA, Blackshaw JA, Young R, Surendran P, Burgess S, Danesh J, et al. PhenoScanner V2: an expanded tool for searching human genotype-phenotype associations. Bioinformatics. 2019;35(22):4851-3.

2. Staley JR, Blackshaw J, Kamat MA, Ellis S, Surendran P, Sun BB, et al. PhenoScanner: a database of human genotype-phenotype associations. Bioinformatics (Oxford, England). 2016;32(20):3207-9.

3. Roddam AW, Allen NE, Appleby P, Key TJ. Endogenous sex hormones and prostate cancer: a collaborative analysis of 18 prospective studies. Journal of the National Cancer Institute. 2008;100(3):170-83.

4. Woodson K, Tangrea JA, Pollak M, Copeland TD, Taylor PR, Virtamo J, et al. Serum insulin-like growth factor I: tumor marker or etiologic factor? A prospective study of prostate cancer among Finnish men. Cancer Res. 2003;63(14):3991-4.

5. Harman SM, Metter EJ, Blackman MR, Landis PK, Carter HB. Serum Levels of Insulin-Like Growth Factor I (IGF-I), IGF-II, IGF-Binding Protein-3, and Prostate-Specific Antigen as Predictors of Clinical Prostate Cancer. The Journal of Clinical Endocrinology & Metabolism. 2000;85(11):4258-65.

6. Morris JK, George LM, Wu T, Wald NJ. Insulin-like growth factors and cancer: no role in screening. Evidence from the BUPA study and meta-analysis of prospective epidemiological studies. Br J Cancer. 2006;95(1):112-7.

7. van den Berg BJ, Christianson RE, Oechsli FW. The California Child Health and Development Studies of the School of Public Health, University of California at Berkeley. Paediatr Perinat Epidemiol. 1988;2(3):265-82.

8. Chen C, Lewis SK, Voigt L, Fitzpatrick A, Plymate SR, Weiss NS. Prostate carcinoma incidence in relation to prediagnostic circulating levels of insulin-like growth factor I, insulin-like growth factor binding protein 3, and insulin. Cancer. 2005;103(1):76-84.

9. Lacey JV, Jr., Hsing AW, Fillmore CM, Hoffman S, Helzlsouer KJ, Comstock GW. Null association between insulin-like growth factors, insulin-like growth factor-binding proteins, and prostate cancer in a prospective study. Cancer epidemiology, biomarkers & prevention : a publication of the American Association for Cancer Research, cosponsored by the American Society of Preventive Oncology. 2001;10(10):1101-2.

10. Allen NE, Key TJ, Appleby PN, Travis RC, Roddam AW, Rinaldi S, et al. Serum insulin-like growth factor (IGF)-I and IGF-binding protein-3 concentrations and prostate cancer risk: results from the European Prospective Investigation into Cancer and Nutrition. Cancer Epidemiol Biomarkers Prev. 2007;16(6):1121-7.

11. Price AJ, Allen NE, Appleby PN, Crowe FL, Travis RC, Tipper SJ, et al. Insulin-like growth factor-I concentration and risk of prostate cancer: results from the European Prospective Investigation into Cancer and Nutrition. Cancer Epidemiol Biomarkers Prev. 2012;21(9):1531-41.

12. Janssen JA, Wildhagen MF, Ito K, Blijenberg BG, Van Schaik RH, Roobol MJ, et al. Circulating free insulin-like growth factor (IGF)-I, total IGF-I, and IGF binding protein-3 levels do not predict the future risk to develop prostate cancer: results of a case-control study involving 201 patients within a population-based screening with a 4-year interval. The Journal of clinical endocrinology and metabolism. 2004;89(9):4391-6.

13. Norman PE, Flicker L, Almeida OP, Hankey GJ, Hyde Z, Jamrozik K. Cohort Profile: The Health In Men Study (HIMS). Int J Epidemiol. 2009;38(1):48-52.

14. Yeap BB, Chubb SAP, Ho KKY, Setoh JWS, McCaul KA, Norman PE, et al. IGF1 and its binding proteins 3 and 1 are differentially associated with metabolic syndrome in older men. European Journal of Endocrinology. 2010;162(2):249-57.

15. Nimptsch K, Platz EA, Pollak MN, Kenfield SA, Stampfer MJ, Willett WC, et al. Plasma insulin-like growth factor 1 is positively associated with low-grade prostate cancer in the Health Professionals Follow-up Study 1993-2004. Int J Cancer. 2011;128(3):660-7.

16. Platz EA, Pollak MN, Leitzmann MF, Stampfer MJ, Willett WC, Giovannucci E. Plasma insulin-like growth factor-1 and binding protein-3 and subsequent risk of prostate cancer in the PSA era. Cancer Causes Control. 2005;16(3):255-62.

17. Cao Y, Nimptsch K, Shui IM, Platz EA, Wu K, Pollak MN, et al. Prediagnostic Plasma IGFBP-1, IGF-1 and Risk of Prostate Cancer. Int J Cancer. 2015;136(10):2418-26.

18. Pham TM, Fujino Y, Nakachi K, Suzuki K, Ito Y, Watanabe Y, et al. Relationship between serum levels of insulin-like growth factors and subsequent risk of cancer mortality: findings from a nested case-control study within the Japan Collaborative Cohort Study. Cancer Epidemiol. 2010;34(3):279-84.

19. Schaefer C, Friedman GD, Quesenberry CP, Orentreich N, Vogelman JH. IGF-I and Prostate Cancer. Science. 1998;282(5387):199-.

20. Severi G, Morris HA, MacInnis RJ, English DR, Tilley WD, Hopper JL, et al. Circulating insulin-like growth factor-I and binding protein-3 and risk of prostate cancer. Cancer Epidemiol Biomarkers Prev. 2006;15(6):1137-41.

21. Gill JK, Wilkens LR, Pollak MN, Stanczyk FZ, Kolonel LN. Androgens, growth factors and risk of prostate cancer: the Multiethnic Cohort. Prostate. 2010;70(8):906-15.

22. Stattin P, Lumme S, Tenkanen L, Alfthan H, Jellum E, Hallmans G, et al. High levels of circulating testosterone are not associated with increased prostate cancer risk: a pooled prospective study. International journal of cancer. 2004;108(3):418-24.

23. Stattin P, Bylund A, Rinaldi S, Biessy C, Dechaud H, Stenman UH, et al. Plasma insulin-like growth factor-I, insulin-like growth factor-binding proteins, and prostate cancer risk: a prospective study. J Natl Cancer Inst. 2000;92(23):1910-7.

24. Stattin P, Rinaldi S, Biessy C, Stenman UH, Hallmans G, Kaaks R. High levels of circulating insulin-like growth factor-I increase prostate cancer risk: a prospective study in a population-based nonscreened cohort. J Clin Oncol. 2004;22(15):3104-12.

25. Neuhouser ML, Platz EA, Till C, Tangen CM, Goodman PJ, Kristal A, et al. Insulin-like growth factors and insulin-like growth factor-binding proteins and prostate cancer risk: results from the prostate cancer prevention trial. Cancer Prev Res (Phila). 2013;6(2):91-9.

26. Chan JM, Stampfer MJ, Giovannucci E, Gann PH, Ma J, Wilkinson P, et al. Plasma insulin-like growth factor-I and prostate cancer risk: a prospective study. Science. 1998;279(5350):563-6.

27. Chan JM, Stampfer MJ, Ma J, Gann P, Gaziano JM, Pollak M, et al. Insulin-like growth factor-I (IGF-I) and IGF binding protein-3 as predictors of advanced-stage prostate cancer. Journal of the National Cancer Institute. 2002;94(14):1099-106.

28. Mucci LA, Stark JR, Pollak MN, Li H, Kurth T, Stampfer MJ, et al. Plasma levels of acid-labile subunit, free insulin-like growth factor-I, and prostate cancer risk: a prospective study. Cancer Epidemiol Biomarkers Prev. 2010;19(2):484-91.

29. Andriole GL, Crawford ED, Grubb RL, 3rd, Buys SS, Chia D, Church TR, et al. Prostate cancer screening in the randomized Prostate, Lung, Colorectal, and Ovarian Cancer Screening Trial: mortality results after 13 years of follow-up. J Natl Cancer Inst. 2012;104(2):125-32.

30. Hayes RB, Reding D, Kopp W, Subar AF, Bhat N, Rothman N, et al. Etiologic and early marker studies in the prostate, lung, colorectal and ovarian (PLCO) cancer screening trial. Control Clin Trials. 2000;21(6 Suppl):349s-55s.

31. Weiss JM, Huang WY, Rinaldi S, Fears TR, Chatterjee N, Chia D, et al. IGF-1 and IGFBP-3: Risk of prostate cancer among men in the Prostate, Lung, Colorectal and Ovarian Cancer Screening Trial. Int J Cancer. 2007;121(10):2267-73.

32. Meyer F, Galan P, Douville P, Bairati I, Kegle P, Bertrais S, et al. A prospective study of the insulin-like growth factor axis in relation with prostate cancer in the SU.VI.MAX trial. Cancer Epidemiol Biomarkers Prev. 2005;14(9):2269-72.

33. Bycroft C, Freeman C, Petkova D, Band G, Elliott LT, Sharp K, et al. The UK Biobank resource with deep phenotyping and genomic data. Nature. 2018;562(7726):203-9.

34. Watts EL, Fensom GK, Smith Byrne K, Perez-Cornago A, Allen NE, Knuppel A, et al. Circulating insulin-like growth factor-I, total and free testosterone concentrations and prostate cancer risk in 200 000 men in UK Biobank. Int J Cancer. 2020.

35. UK Biobank. Biomarker assay quality procedures: approaches used to minimise systematic and random errors (and the wider epidemiological implications) 2019 [Available from: <http://biobank.ctsu.ox.ac.uk/showcase/docs/biomarker_issues.pdf>.

36. Thompson IM, Goodman PJ, Tangen CM, Lucia MS, Miller GJ, Ford LG, et al. The influence of finasteride on the development of prostate cancer. N Engl J Med. 2003;349(3):215-24.

37. Schumacher FR, Al Olama AA, Berndt SI, Benlloch S, Ahmed M, Saunders EJ, et al. Association analyses of more than 140,000 men identify 63 new prostate cancer susceptibility loci. Nature Genetics. 2018;50(7):928-36.

38. Wu L, Wang J, Cai Q, Cavazos TB, Emami NC, Long J, et al. Identification of novel susceptibility loci and genes for prostate cancer risk: a transcriptome-wide association study in over 140,000 European descendants. Cancer research. 2019;79(13):3192-204.

39. Key TJ, Appleby PN, Allen NE, Reeves GK. Pooling biomarker data from different studies of disease risk, with a focus on endogenous hormones. Cancer epidemiology, biomarkers & prevention : a publication of the American Association for Cancer Research, cosponsored by the American Society of Preventive Oncology. 2010;19(4):960-5.

40. Watts EL, Appleby PN, Perez-Cornago A, Bueno-de-Mesquita HB, Chan JM, Chen C, et al. Low free testosterone and prostate cancer risk: a collaborative analysis of 20 prospective studies. Eur Urol. 2018;74(5):585-94.

41. Travis RC, Appleby PN, Martin RM, Holly JM, Albanes D, Black A, et al. A meta-analysis of individual participant data reveals an association between circulating levels of IGF-I and prostate cancer risk. Cancer research. 2016;76(8):2288-300.

42. NealeLab;. UK Biobank GWAS Results 2019 [Available from: <http://www.nealelab.is/uk-biobank>.

43. Bycroft Cea. Genome-wide genetic data on ~500,000 UK Biobank participants. 2017.

44. Conti DV, Darst BF, Moss LC, Saunders EJ, Sheng X, Chou A, et al. Trans-ancestry genome-wide association meta-analysis of prostate cancer identifies new susceptibility loci and informs genetic risk prediction. Nature genetics. 2021;53(1):65-75.

45. Hemani G, Zheng J, Elsworth B, Wade KH, Haberland V, Baird D, et al. The MR-Base platform supports systematic causal inference across the human phenome. eLife. 2018;7:e34408.

46. Burgess S, Bowden J. Integrating summarized data from multiple genetic variants in Mendelian randomization: bias and coverage properties of inverse-variance weighted methods. arXiv preprint arXiv:151204486. 2015.

47. Bowden J, Del Greco M F, Minelli C, Davey Smith G, Sheehan NA, Thompson JR. Assessing the suitability of summary data for two-sample Mendelian randomization analyses using MR-Egger regression: the role of the I2 statistic. International Journal of Epidemiology. 2016;45(6):1961-74.

48. Palmer TM, Sterne JA, Harbord RM, Lawlor DA, Sheehan NA, Meng S, et al. Instrumental variable estimation of causal risk ratios and causal odds ratios in Mendelian randomization analyses. American journal of epidemiology. 2011;173(12):1392-403.

49. Burgess S, Thompson SG. Avoiding bias from weak instruments in Mendelian randomization studies. Int J Epidemiol. 2011;40(3):755-64.

50. Burgess S, Bowden J, Fall T, Ingelsson E, Thompson SG. Sensitivity Analyses for Robust Causal Inference from Mendelian Randomization Analyses with Multiple Genetic Variants. Epidemiology. 2017;28(1):30-42.

51. Verbanck M, Chen C-Y, Neale B, Do R. Detection of widespread horizontal pleiotropy in causal relationships inferred from Mendelian randomization between complex traits and diseases. Nature Genetics. 2018;50(5):693-8.

52. Burgess S, Thompson SG. Interpreting findings from Mendelian randomization using the MR-Egger method. Eur J Epidemiol. 2017;32(5):377-89.

53. Bowden J, Davey Smith G, Haycock PC, Burgess S. Consistent Estimation in Mendelian Randomization with Some Invalid Instruments Using a Weighted Median Estimator. Genet Epidemiol. 2016;40(4):304-14.

54. Hemani G, Tilling K, Davey Smith G. Orienting the causal relationship between imprecisely measured traits using GWAS summary data. PLoS Genet. 2017;13(11):e1007081.

55. Burgess S, Foley CN, Allara E, Staley JR, Howson JMM. A robust and efficient method for Mendelian randomization with hundreds of genetic variants. Nature Communications. 2020;11(1):376.

56. Holmes MV, Richardson TG, Ference BA, Davies NM, Davey Smith G. Integrating genomics with biomarkers and therapeutic targets to invigorate cardiovascular drug development. Nat Rev Cardiol. 2021.

57. Giambartolomei C, Vukcevic D, Schadt EE, Franke L, Hingorani AD, Wallace C, et al. Bayesian test for colocalisation between pairs of genetic association studies using summary statistics. PLoS Genet. 2014;10(5):e1004383.

58. Wallace C. A more accurate method for colocalisation analysis allowing for multiple causal variants. PLOS Genetics. 2021;17(9):e1009440.

59. Deng Y, Pan W. A powerful and versatile colocalization test. PLoS Comput Biol. 2020;16(4):e1007778-e.

60. Zheng J, Haberland V, Baird D, Walker V, Haycock PC, Hurle MR, et al. Phenome-wide Mendelian randomization mapping the influence of the plasma proteome on complex diseases. Nature genetics. 2020;52(10):1122-31.

61. Liu B, Gloudemans MJ, Rao AS, Ingelsson E, Montgomery SB. Abundant associations with gene expression complicate GWAS follow-up. Nat Genet. 2019;51(5):768-9.

62. McKay JD, Hung RJ, Han Y, Zong X, Carreras-Torres R, Christiani DC, et al. Large-scale association analysis identifies new lung cancer susceptibility loci and heterogeneity in genetic susceptibility across histological subtypes. Nature genetics. 2017;49(7):1126-32.
